# Activin A activation of Smad3 mitigates innate inflammation in mouse models of psoriasis and sepsis

**DOI:** 10.1172/JCI187063

**Published:** 2025-03-11

**Authors:** Thierry Gauthier, Yun-Ji Lim, Wenwen Jin, Na Liu, Liliana C. Patiño, Weiwei Chen, James Warren, Daniel Martin, Robert J. Morell, Gabriela Dveksler, Gloria H. Su, WanJun Chen

**Affiliations:** 1 Mucosal Immunology Section, National Institute of Dental and Craniofacial Research, National Institutes of Health, Bethesda, Maryland, USA.; 2 Department of Pathology, Uniformed Services University, Bethesda, Maryland, USA.; 3 Genomics and Computational Biology Core, National Institute on Deafness and Other Communication Disorders, National Institutes of Health, Bethesda, Maryland, USA.; 4 Department of Pathology and Cell Biology, Columbia University Irving Medical Center, New York, New York, USA.

**Keywords:** Immunology, Inflammation, Cytokines, Innate immunity, Macrophages

## Abstract

Phosphorylation of Smad3 is a critical mediator of TGF-β signaling, which plays an important role in regulating innate immune responses. However, whether Smad3 activation can be regulated in innate immune cells in TGF-β–independent contexts remains poorly understood. Here, we show that Smad3 is activated through the phosphorylation of its C-terminal residues (pSmad3C) in murine and human macrophages in response to bacterial and viral ligands, and this activation is mediated by activin A in a TGF-β–independent manner. Specifically, infectious ligands, such as LPS, induced secretion of activin A through the transcription factor STAT5 in macrophages, and activin A signaling in turn activated pSmad3C. This activin A/Smad3 axis controlled mitochondrial ATP production and ATP conversion into adenosine by CD73 in macrophages, enforcing an antiinflammatory mechanism. Consequently, mice with a deletion of activin A receptor 1b specifically in macrophages (*Acvr1b^fl/fl^-*Lyz2cre) succumbed more to sepsis as a result of uncontrolled inflammation and exhibited exacerbated skin disease in a mouse model of imiquimod-induced psoriasis. Thus, we have revealed a previously unrecognized natural brake to inflammation in macrophages that occurs through the activation of Smad3 in an activin A–dependent manner.

## Abstract 2

We uncovered here that activation of the transcription factor Smad3 by Activin A in macrophages is a natural brake to innate inflammation. This axis is notably active during sepsis and Covid-19 infections, as well as during psoriasis. As this pathway is conserved in humans, it may be targeted to harness new therapeutic strategies during infections and autoimmunity.

## Graphical abstract

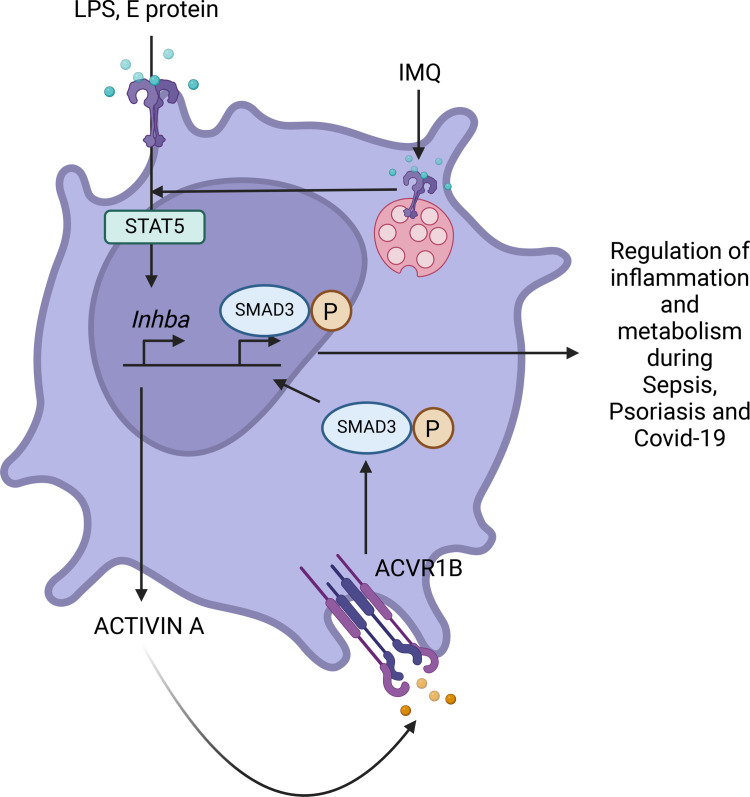

## Introduction

Macrophages are crucial for the mounting of a proper immune response. Their activation and polarization toward a proinflammatory phenotype enable them to clear pathogens and protect our bodies during infections (1, 2). However, they need intrinsic brakes to avoid overt inflammation and tissue damage and promote resolution and the return to homeostasis (3–6). A way for innate immune cells to avoid overt inflammation is to produce antiinflammatory cytokines such as interleukin-10 (IL-10) and transforming growth factor-β1 (TGF-β1) (7, 8). TGF-β1 belongs to the TGF-β superfamily of proteins, which is composed of TGF-βs, activins, Nodal, growth differentiation factors (GDFs), and bone morphogenetic proteins (BMPs). TGF-β, after binding to its receptors, signals through a canonical pathway involving the Smad proteins. TGF-β promotes the phosphorylation of Smad3 in its C-terminal domain (serine 423 and 425; hereafter called pSmad3C), which induces the translocation of Smad3 to the nucleus and its binding onto the promoter of its target genes (9–11). This TGF-β/Smad3 axis has been described to be active in macrophages and to regulate inflammation (12, 13). It has also been described that lipopolysaccharide (LPS) could transactivate TGF-β receptor I via Toll-like receptor 4 (TLR4), suggesting that the TGF-β signaling could be regulated by inflammatory stimuli (14). However, whether Smad3 can be activated by stimuli other than TGF-β, especially in the context of inflammation-mediated environments, remains poorly understood.

Here, we discovered that bacterial and viral ligands such as LPS can induce pSmad3C in an activin A–dependent but TGF-β–independent manner. LPS activated a pathway linked to MEK/ERK kinases and STAT5 to promote the expression of the gene encoding activin A (*Inhba*) and its receptors. Activin A induced pSmad3C in vitro and in vivo in inflammatory models of sepsis and psoriasis and, importantly, also in human macrophages. We determined that this activin A/Smad3 axis was a natural brake to inflammation, which prevented the overt activation of macrophages in response to inflammatory stimuli and suppressed inflammation in experimental sepsis and psoriasis. Thus, our findings shed light on a previously unrecognized natural brake to inflammation in macrophages that occurs by the activation of Smad3 in an activin A–dependent manner.

## Results

### LPS induces pSmad3C in a TGF-β–independent manner in vitro.

**Figure 1:**
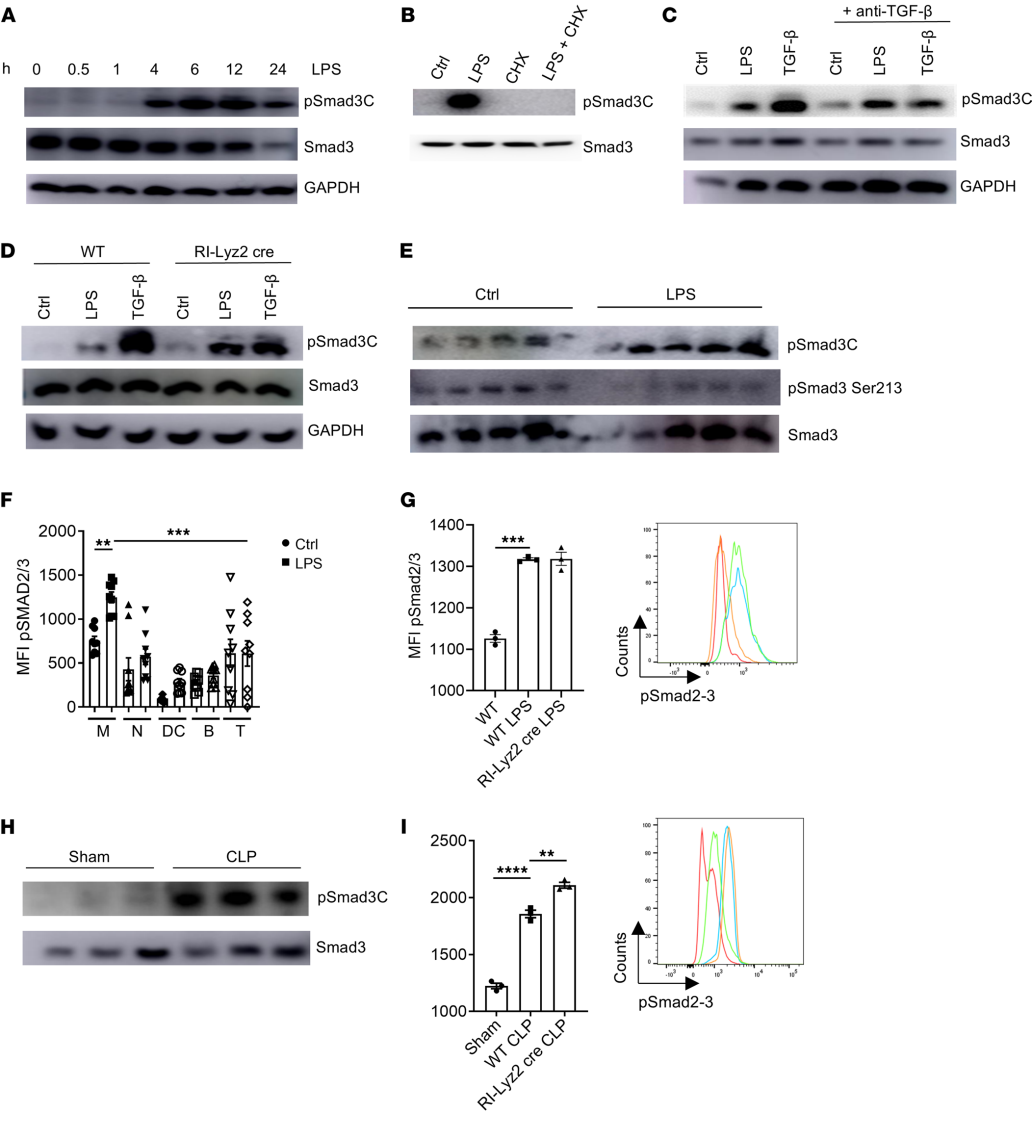
LPS activates Smad3 in a TGF-β–independent manner. (**A**) Abundance of the indicated proteins in macrophages treated with 10 ng/mL of LPS for the indicated times. (**B**) Abundance of the indicated proteins in macrophages pretreated with cycloheximide for 1 hour followed by LPS stimulation for 6 hours. (**C**) Abundance of the indicated proteins in macrophages pretreated with an anti–TGF-β blocking antibody for 1 hour followed by LPS or TGF-β (5 ng/mL) stimulation for 6 hours. (**D**) Abundance of the indicated proteins in macrophages isolated from WT or RI-Lyz2cre mice and stimulated by LPS or TGF-β for 6 hours. (**E**) Abundance of the indicated proteins in peritoneal cells of mice injected i.p. with LPS and harvested 6 hours after injection. Each band represents a mouse. (**F**) Flow cytometry analysis of phosphorylated Smad2/3 (pSmad2/3) levels in peritoneal cells from mice injected i.p. with LPS and harvested 6 hours after injection. MFI, mean fluorescence intensity; M, macrophages; N, neutrophils; DC, dendritic cells; B, B cells; T, T cells. (*n* = 9.) (**G**) Flow cytometry analysis of pSmad2/3 levels in macrophages from WT or RI-Lyz2cre mice injected i.p. with LPS and harvested 6 hours after injection. Red, isotype control; orange, WT; blue, WT LPS; green, RI-Lyz2cre LPS. (**H**) Abundance of the indicated proteins in peritoneal cells of mice subjected to CLP surgery (or sham surgery) and harvested 6 hours after injection. Each band represents a mouse. (**I**) Flow cytometry analysis of pSmad2/3 levels in macrophages from WT or RI-Lyz2cre mice subjected to CLP surgery (or sham surgery) and harvested 6 hours after injection. Red, isotype control; green, WT; blue, WT LPS; orange, RI-Lyz2cre LPS. Representative or pooled from at least 2 independent experiments. **P < 0.01, ***P < 0.005, ****P < 0.001 by 1-way ANOVA.

It is well known that TGF-β signaling activates the transcription factor Smad3 through phosphorylation in innate and adaptive immune cells (12, 15, 16). However, we unexpectedly discovered that LPS induced Smad3 phosphorylation in its C-terminal domain (serine 423/425; hereafter referred to as pSmad3C) in murine macrophages. Specifically, LPS-induced pSmad3C started 4 hours after treatment, peaked at 6 hours, and stayed stable until 24 hours in in vitro cultures (Figure 1A and Supplemental Figure 1A; Western blotting quantifications are presented in Supplemental Figures 13 and 14; supplemental material available online with this article; https://doi.org/10.1172/JCI187063DS1), whereas the levels of total SMAD3 protein were largely unaltered (except for a reduction at 24 hours, suggesting a negative retro-control mechanism) (Figure 1A). Smad3 phosphorylation can also occur in the linker region (serine 213 and tyrosine 179) in response to TGF-β and/or other factors (17); however, LPS was unable to induce Smad3 phosphorylation in these sites in the linker region (Supplemental Figure 1A and data not shown), suggesting the specificity of C-terminal phosphorylation of Smad3. The fact that Smad3 was not phosphorylated until 4 hours after LPS treatment suggested that pSmad3C was induced via the synthesis of new proteins rather than through a direct LPS-mediated signaling. Indeed, the translation inhibitor cycloheximide completely abrogated pSmad3C, confirming the requirement of new protein synthesis (Figure 1B). Since TGF-β1 is the primary inducer of pSmad3C, we initially hypothesized that LPS induced pSmad3C by promoting the production of TGF-β and/or enhancing its signaling, which in turn acted in a paracrine or autocrine manner. We first determined that the *Tgfb1* mRNA was not upregulated until 6 hours after LPS treatment, which was later than the appearance of pSmad3C at 4 hours (Supplemental Figure 1B). Importantly, we blocked the TGF-β signaling by using the specific antibody 1D11 that neutralizes TGF-β1, 2, and 3 in wild-type (WT) macrophages (Figure 1C and Supplemental Figure 1C) (18, 19) or by using macrophages from RI-Lyz2cre mice (*Tgfbr1^flox/flox^* crossed with Lyz2cre^+^ mice) (12) in which *Tgfbr1* was deleted specifically in macrophages (Figure 1D and Supplemental Figure 1D), and treated these macrophages with LPS to measure pSmad3C. In all aforementioned conditions, the pSmad3C induction by LPS was unchanged in comparison with the respective WT controls, while the TGF-β induction of pSmad3C was abrogated as expected (Figure 1, C and D). These data collectively demonstrate that LPS induces pSmad3C in a manner independent of TGF-β signaling.

### Macrophages induce pSmad3C in LPS- or cecal ligation puncture–induced sepsis in mice independently of TGF-β.

We next investigated whether Smad3 could be phosphorylated in vivo during LPS-induced endotoxin shock and cecal ligation puncture–induced (CLP-induced) sepsis in mice. In line with our in vitro data, intraperitoneal injection of LPS induced a dramatic increase in pSmad3c in peritoneal cells (Figure 1E). On the other hand, the levels of pSmad3 serine 213 in the Smad3 linker region were not upregulated, and the levels of pSmad3 tyrosine 179 were not detected in all the samples (data not shown), confirming the specific activation of pSmad3C upon LPS stimulation. Among the peritoneal cells, we observed that macrophages were the main population in which pSmad3C was significantly increased after LPS treatment (Figure 1F; see Supplemental Figure 1E for gating strategy). Importantly, the induction of pSmad3C remained unaffected in RI-Lyz2cre mice, indicating that TGF-β is indeed dispensable for Smad3 activation in response to LPS (Figure 1G). Strikingly, a similar phenomenon was observed during CLP-induced sepsis (Figure 1, H and I, and Supplemental Figure 1F). The data collectively confirm that LPS induces pSmad3C in a TGF-β–independent manner in vivo during sepsis.

### LPS activation of Smad3 requires activin A in murine and human macrophages.

**Figure 2:**
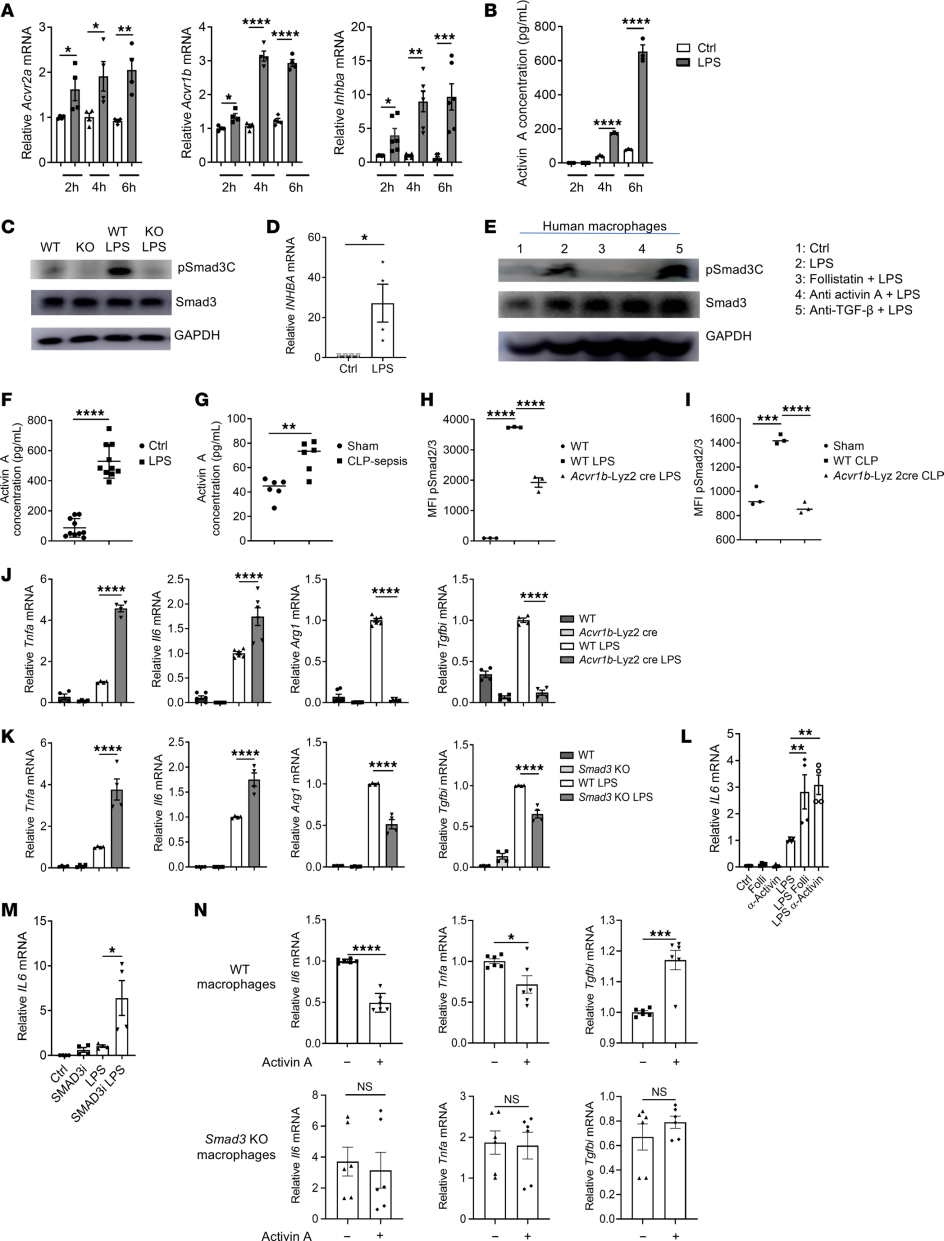
LPS phosphorylates Smad3 in an activin A–dependent manner. (**A**) RT-qPCR analysis in macrophages stimulated by LPS. (*n* = 4–6.) (**B**) Activin A levels (measured by ELISA) in the supernatant of macrophages stimulated by LPS. (**C**) Abundance of the indicated proteins in macrophages stimulated by LPS for 6 hours. (**D**) RT-qPCR analysis of *INHBA* expression in human macrophages stimulated by LPS for 6 hours. (*n* = 4.) (**E**) Abundance of the indicated proteins in human macrophages pretreated with follistatin or an anti–activin A or anti–TGF-β blocking antibody for 1 hour followed by LPS stimulation for 6 hours. (**F**–**G**) Activin A levels in serum of WT mice injected with LPS (**F**) or subjected to CLP surgery (**G**) and harvested after 6 hours. (*n* = 6–10.) (**H**–**I**) Flow cytometry analysis of pSmad2/3 levels in macrophages from WT or *Acvr1b*-Lyz2cre mice injected i.p. with LPS (**H**) or subjected to CLP surgery (**I**) and harvested after 6 hours. (**J**) RT-qPCR analysis of the indicated genes in macrophages stimulated or not by LPS for 24 hours. (*n* = 4–6.) (**K**) RT-qPCR analysis of macrophages stimulated or not by LPS for 24 hours. (*n* = 4.) (**L**) RT-qPCR analysis of *IL6* expression in human macrophages pretreated with follistatin or an anti–activin A blocking antibody for 1 hour followed by LPS stimulation for 24 hours. (*n* = 4.) (**M**) RT-qPCR analysis of *IL6* expression in human macrophages pretreated with a Smad3 inhibitor (SMAD3i) for 1 hour followed by LPS stimulation for 24 hours. (*n* = 4.) (**N**) RT-qPCR analysis in macrophages stimulated by LPS for 24 hours in the presence of activin A. (*n* = 6.) Representative or pooled from at least 2 independent experiments. *P < 0.05, **P < 0.01, ***P < 0.005, ****P < 0.001 by Student’s *t* test (**A**, **B**, **D**, **F**, **G**, and **N**) or 1-way ANOVA (**H**–**M**).

We next determined the mechanisms by which LPS induces pSmad3C. To address this, we first performed quantitative reverse transcriptase PCR (RT-qPCR) on the genes related to the TGF-β superfamily of proteins in macrophages. We observed that the levels of *Smad2*, *Smad3*, *Bambi*, and *Acvr2b* were not significantly changed after LPS treatment (Supplemental Figure 2A). The expression of the inhibitory Smads, e.g., *Smad6* and *Smad7*, was decreased after 2 hours of LPS treatment, but this effect was transient (Supplemental Figure 2A). We also did not detect any expression of *Inha* and *Inhbb* (both encoding subunits of inhibin). However, starting at 2 hours after LPS treatment and peaking at 6 hours, the levels of expression of the activin A receptors *Acvr2a* and *Acvr1b* were significantly increased. Strikingly, the mRNA of *Inhba* (encoding activin A) and the protein levels of activin A were both markedly increased with a peak at 6 hours (Figure 2, A and B). Interestingly, *Inha*, *Inhbb*, and *Acvr1c* (which are other molecules associated with activin signaling) were not expressed in macrophages (data not shown). This suggests a role of activin A in the phosphorylation of Smad3 in response to LPS. Indeed, blockade of the activin A effect using the natural activin A inhibitor follistatin or an activin A–blocking antibody completely abrogated the pSmad3C induced by LPS (Supplemental Figure 3A). We also confirmed that the blocking antibody indeed blocked the ability of activin A to induce pSmad3C (Supplemental Figure 3B). To further confirm this, we next generated *Acvr1b*-Lyz2cre mice by crossing *Acvr1b^fl/fl^* mice with Lyz2cre^+^ mice to study the effect of activin A receptor deletion on pSmad3C in macrophages (20). We first confirmed that the recombination between *Acvr1b* and Lyz2cre was efficient (Supplemental Figure 3C) and that the activin A signaling (and its ability to induce pSmad3C) was blocked in *Acvr1b*-Lyz2cre macrophages (Supplemental Figure 3D), altogether confirming the deletion of *Acvr1b*. We also showed that pSmad3C was completely abrogated in these knockout macrophages in response to LPS (Figure 2C), confirming that the activin A signaling is indeed responsible for Smad3 phosphorylation. Importantly, expression of *INHBA* (encoding human activin A) was also induced after LPS treatment in human macrophages (Figure 2D), and the induction of pSMAD3C by LPS was abolished by activin A blockade while the inhibition of TGF-β did not affect it (Figure 2E).

Having elucidated that LPS-induced pSmad3C occurs through activin A in macrophages in vitro, we next showed that intraperitoneal injection of LPS (endotoxin model) or CLP surgery (sepsis model) induced a significant increase in activin A protein in the blood of mice (Figure 2, F and G). In macrophages, we observed that the induction of pSmad3C was abrogated in *Acvr1b*-Lyz2cre mice, indicating that activin A is indeed indispensable for Smad3 activation in sepsis models induced by LPS and CLP surgery (Figure 2, H and I). Thus, the LPS induction of pSmad3C is dependent on activin A signaling in both murine and human macrophages.

### The activin A/SMAD3 axis restrains LPS-induced inflammation in macrophages.

We next investigated the function of the activin Smad3 axis in macrophages. Blockade of activin A or its downstream target Smad3 resulted in enhanced mRNA expression of proinflammatory cytokines including *Tnf*, *Il6*, *Cxcl9*, and *Il27*, but decreased the antiinflammatory genes *Tgfbi* and *Arg1* (Figure 2, J and K, and Supplemental Figure 3, E–G). Importantly, this axis is specific for some genes, since *Il1b* was not affected by *Smad3* or *Acvr1b* deletions, and *Il10* was only affected by *Acvr1b* deletion (suggesting additional effects of activin A that might be independent of Smad3) (Supplemental Figure 3, F and G). Consistently, blockade of activin A also significantly increased the protein levels of TNF-α and IL-6 in macrophages treated by LPS (Supplemental Figure 3H). Importantly, human macrophages treated by LPS in the presence of follistatin or anti–activin A antibody exhibited significantly higher levels of *Il6* expression compared with LPS-treated macrophages alone (Figure 2L). Similarly, inhibition of Smad3 function (using a Smad3 inhibitor) also increased the expression of *IL6* in human macrophages (Figure 2M).

We then further determined that Smad3 was signaling downstream of activin A to decrease inflammation. We showed that the inhibition of Smad3 with its specific inhibitor blocked the activin A–induced suppression of *Il6* gene expression in normal macrophages (Supplemental Figure 3I). In addition, *Smad3*-KO macrophages exhibited a severe defect in the suppression of *Il6* and *Tnfa* and an increase in *Tgfbi* in response to activin A treatment when compared with WT macrophages (Figure 2N). The data collectively indicate that activin A–mediated pSmad3C controls inflammatory cytokines in macrophages in response to LPS.

### LPS induces the activin A/pSmad3C axis through STAT5.

**Figure 3:**
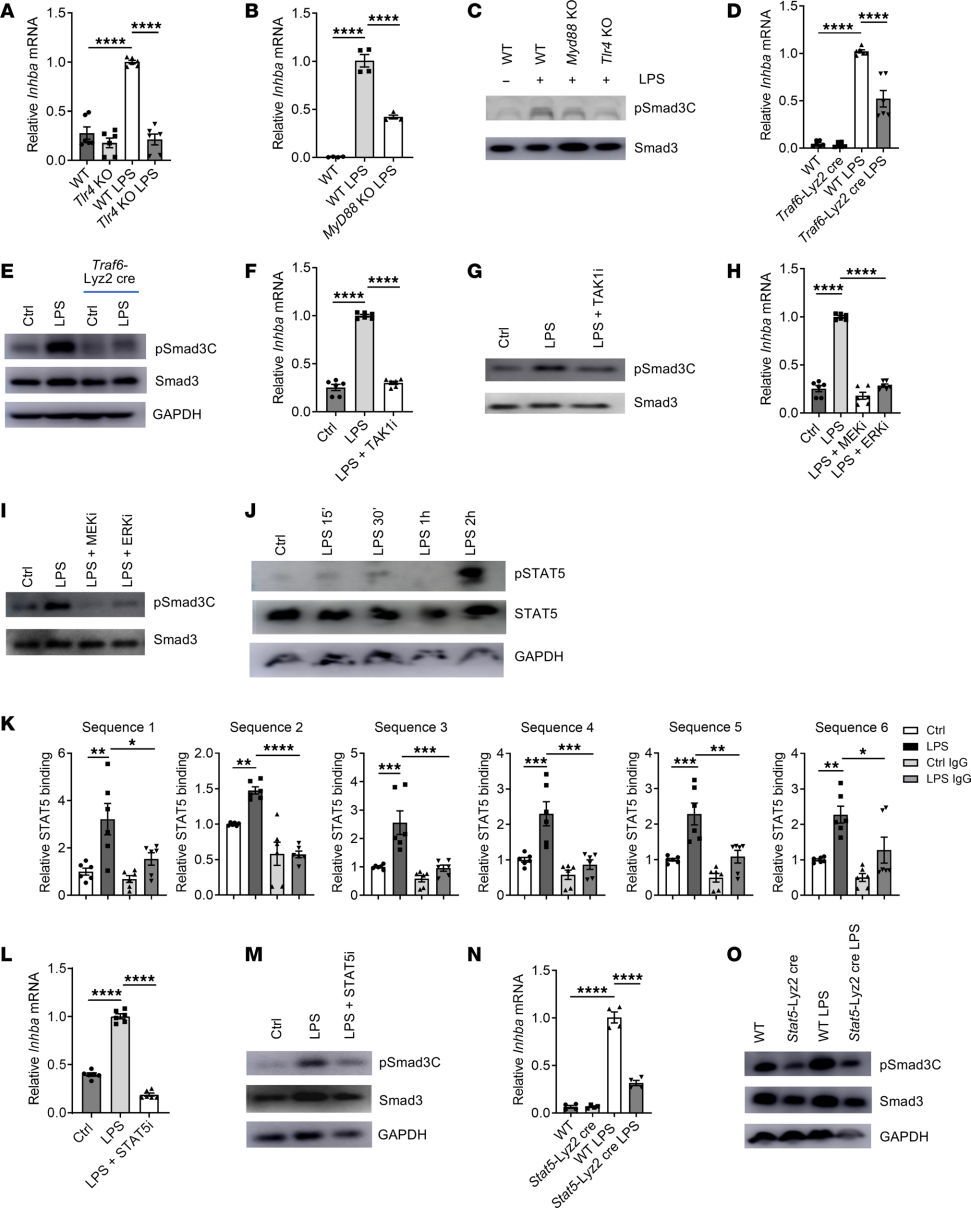
LPS induces the activin A/Smad3 axis through a *TLR4*/*MyD88*/MAPK/STAT5 pathway. (**A**) RT-qPCR analysis of *Inhba* expression in macrophages from WT or *TLR4*-KO mice stimulated 2 hours by LPS. (*n* = 6.) (**B**) RT-qPCR analysis of *Inhba* expression in macrophages from WT or *MyD88*-KO mice stimulated 2 hours by LPS. (*n* = 4.) (**C**) Protein abundance in macrophages stimulated 6 hours by LPS. (**D**) RT-qPCR analysis of *Inhba* expression in macrophages from WT or *Traf6*-Lyz2cre mice stimulated by LPS for 2 hours. (*n* = 6.) (**E**) Protein abundance in macrophages isolated stimulated 6 hours by LPS. (**F**) RT-qPCR analysis of *Inhba* expression in macrophages pretreated with a TAK1 inhibitor for 1 hour followed by LPS stimulation for 2 hours. (*n* = 6.) (**G**) Protein abundance in macrophages pretreated with a TAK1 inhibitor for 1 hour followed by LPS stimulation for 6 hours. (**H**) RT-qPCR analysis of *Inhba* expression in macrophages pretreated with MEK and ERK inhibitors for 1 hour followed by LPS stimulation for 2 hours. (*n* = 6.) (**I**) Protein abundance in macrophages pretreated 1 hour with MEK and ERK inhibitors followed by LPS stimulation for 6 hours. (**J**) Protein abundance in macrophages treated with LPS. (**K**) ChIP-coupled real-time PCR analysis of STAT5 enrichment in various sequences of the promoter region of the *Inhba* gene in macrophages treated with LPS for 2 hours. (*n* = 6.) (**L**) RT-qPCR analysis of *Inhba* expression in macrophages pretreated 1 hour with a STAT5 inhibitor followed by LPS stimulation for 2 hours. (*n* = 6.) (**M**) Protein abundance in macrophages pretreated 1 hour with a STAT5 inhibitor followed by LPS stimulation for 6 hours. (**N**) RT-qPCR analysis of *Inhba* expression in macrophages from WT or *Stat5*-Lyz2cre mice stimulated by LPS for 2 hours. (*n* = 6.) (**O**) Protein abundance in macrophages stimulated by LPS for 6 hours. Representative of at least 2 independent experiments. *P < 0.05, **P < 0.01, ***P < 0.005, ****P < 0.001 by 1-way ANOVA.

Next, we deciphered the signaling pathway leading to activin A expression and consequent Smad3C phosphorylation in response to LPS. We first observed that this axis was dependent on TLR4 and myeloid differentiation primary response 88 (MyD88), since macrophages deficient in these genes exhibited severely deficient *Inhba* expression and decreased Smad3C phosphorylation in response to LPS (Figure 3, A–C). However, macrophages with deficient TIR domain–containing adapter-inducing interferon-β (TRIF; encoded by *Ticam1*) showed normal pSmad3C upon LPS stimulation, suggesting that TRIF is dispensable to activate the activin A/Smad3 pathway by LPS (Supplemental Figure 4A). As TNF receptor–associated factor 6 (TRAF6) is a key molecule downstream of MyD88, and a crucial activator of transforming growth factor-β–activated kinase 1 (TAK1) (21), we next examined the function of TRAF6 and TAK1 in LPS-induced activin A/pSmad3C. We first showed that macrophages deficient in *Traf6* (*Traf6*-Lyz2cre) expressed markedly reduced *Inhba* mRNA and pSmad3C (Figure 3, D and E). Similarly, suppression of TAK1 activity with its specific inhibitor also significantly decreased the *Inhba* gene expression and substantially blocked pSmad3C induced by LPS (Figure 3, F and G), suggesting a critical role of TRAF6-TAK1 in this pathway (Figure 3, D–G). Finally, we determined that the MAP kinases MAP kinase kinase (MEK) and extracellular signal–regulated kinase (ERK), which have been described to be downstream molecules of TRAF6 and TAK1 in the LPS signaling cascade, were crucial for the axis (21), since inhibition of MEK and ERK activity abolished LPS-induced expression of activin A and consequently the pSmad3C induction (Figure 3, H and I).

We next searched for the transcription factor(s) that could be regulated by this TLR4-MyD88-TRAF6/TAK1-MEK/ERK pathway. We first observed that the transcription factor STAT5 possessed some sites predicted to bind into the *Inhba* promoter, suggesting that STAT5 could be responsible for the induction of *Inhba* expression (Supplemental Figure 4B). We then demonstrated that STAT5 was phosphorylated after LPS treatment in a timeline that was consistent with the induction of *Inhba* expression (less than 2 hours after LPS treatment), while the expression of total *Stat5a* and *Stat5b* mRNA remained unchanged (Figure 3J and Supplemental Figure 4C). Importantly, the phosphorylation of STAT5 was dependent on TRAF6, TAK1, MEK, and ERK, as blockade of each of these molecules substantially inhibited the STAT5 activation (Supplemental Figure 4, D and E). At the molecular level, we found that the binding of STAT5 to several sites at the *Inhba* promoter was significantly increased at 3 hours after LPS treatment (Figure 3K). Importantly, STAT5 inhibition in WT macrophages or the use of *Stat5*-Lyz2cre macrophages abrogated both the expression of *Inhba* and pSmad3C induced by LPS (Figure 3, L–O). The validation of the different proteins’ knockdown and inhibitor selectivity from Figure 3 is presented in Supplemental Figure 5. Thus, the data collectively reveal that STAT5 activation plays a key role in LPS-induced activin A production and pSmad3C activation.

### The activin A/Smad3 axis controls ATP metabolism.

To understand the mechanisms underlying this activin A/Smad3 function in response to LPS-induced inflammation, we performed RNA-Seq analysis on macrophages from WT or *Smad3*-KO mice treated with LPS for 24 hours. *Smad3-*KO macrophages exhibited a largely remodeled phenotype with 1,444 genes upregulated and 1,216 downregulated compared with WT macrophages (Supplemental Figure 6A). Analysis of the pathways up- and downregulated showed that *Smad3*-KO macrophages had a large increase in proinflammatory pathways (for example, response to virus, regulation of defense response, cell activation, etc.) while the downregulated pathways were largely enriched in metabolic pathways (metabolism of lipids, carbohydrate metabolic pathway, metabolism of carbohydrate, etc.) (Supplemental Figure 6B), suggesting that the activin A/Smad3 pathway regulates macrophage immunometabolism in response to LPS.

**Figure 4:**
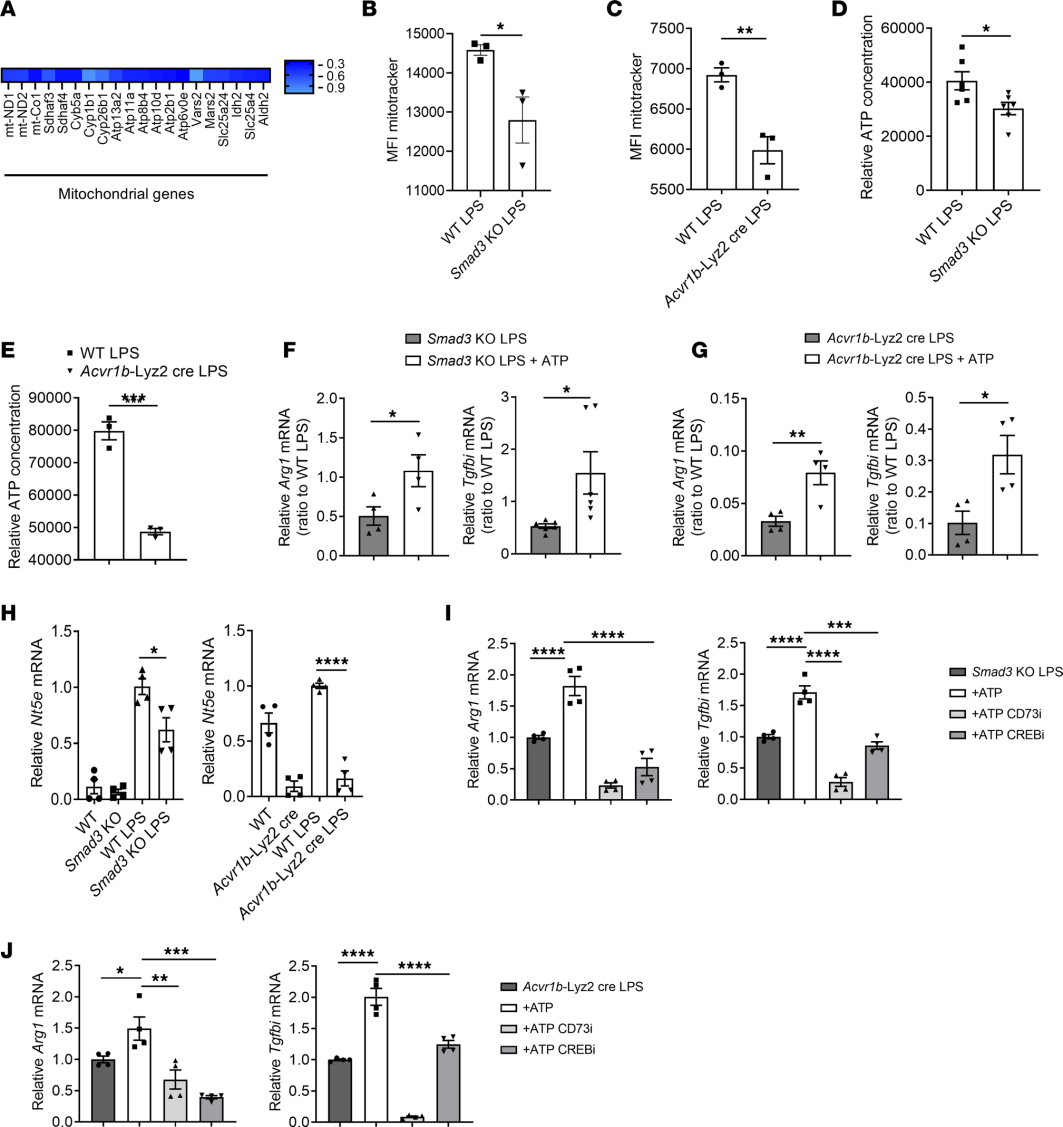
The activin A/Smad3 pathway supports ATP metabolism during inflammation. (**A**) Heatmap representing significantly downregulated genes in macrophages from *Smad3*-KO mice (compared with WT macrophages) stimulated with LPS for 24 hours. (**B** and **C**) MitoTracker staining in macrophages stimulated with LPS for 24 hours and isolated from *Smad3*-KO mice (**B**) or *Acvr1b*-Lyz2cre mice (**C**). (**D** and **E**) ATP production (intracellular) in macrophages stimulated with LPS for 24 hours and isolated from *Smad3*-KO mice (**D**) (*n* = 6) or *Acvr1b*-Lyz2cre mice (**E**). (**F** and **G**) RT-qPCR analysis of *Arg1* and *Tgfbi* expression in macrophages stimulated with LPS for 24 hours in combination (or not) with 20 μM of ATP and isolated from *Smad3*-KO mice (**F**) or *Acvr1b*-Lyz2cre mice (**G**). (**H**) RT-qPCR analysis of *Nt5e* (encoding CD73) expression in macrophages stimulated with LPS for 24 hours and isolated from *Smad3*-KO or *Acvr1b*-Lyz2cre mice. (**I** and **J**) RT-qPCR analysis of *Tgfbi* and *Arg1* expression in macrophages stimulated with LPS for 24 hours in combination (or not) with ATP and a CD73 inhibitor or a CREB inhibitor and isolated from *Smad3*-KO mice (**I**) or *Acvr1b*-Lyz2cre mice (**J**). (**F**–**J**, *n* = 4.) Pooled or representative of at least 2 independent experiments. *P < 0.05, **P < 0.01, ***P < 0.005, ****P < 0.001 by Student’s *t* test (**B**–**G**) or 1-way ANOVA (**H**–**J**).

Among the metabolic genes regulated by Smad3, several related to mitochondria metabolism and functions were downregulated (Figure 4A). Since mitochondria are a critical hub to regulate macrophage immunometabolism (22–24), we next investigated whether the activin A/pSmad3C pathway regulated mitochondrial functions in response to LPS. Interestingly, we observed that when *Smad3*-KO or *Acvr1b*-Lyz2cre macrophages treated with LPS were stained with MitoTracker Green to quantify mitochondria, these KO macrophages had decreased mitochondrial numbers compared with WT macrophages (Figure 4, B and C, and Supplemental Figure 7, A and B). However, the levels of mitochondrial membrane potential (ϕm) and mitochondrial reactive oxygen species (ROS) were similar between the *Smad3*-KO and WT macrophages (Supplemental Figure 7C). Similarly, WT macrophages treated with follistatin or anti–activin A antibody had decreased levels of mitochondria but unchanged levels of ϕm and mitochondrial ROS (Supplemental Figure 7, D and E). One of the critical functions of mitochondria is to generate energy via the production of ATP. Indeed, *Smad3*-KO and *Acvr1b*-Lyz2cre macrophages also had decreased ATP levels (Figure 4, D and E). Given the critical role of ATP in regulating cellular functions (25), we reasoned that restoring the levels of ATP by supplementing exogenous ATP (at low amounts to avoid inflammasome activation and cell death) in the culture would reverse the proinflammatory phenotype observed in *Smad3*-KO and *Acvr1b*-Lyz2cre macrophages. Indeed, we found that the levels of *Arg1* and *Tgfbi* were significantly increased after ATP treatment in these knockout macrophages (Figure 4, F and G). Similar increases in *Arg1* and *Tgfbi* were also observed in follistatin-treated WT macrophages (Supplemental Figure 7F). These results demonstrate that the disruption of the activin A/Smad3 axis dysregulates ATP production, which regulates the expression of *Arg1* and *Tgfbi* in LPS-activated macrophages. Importantly, most of these changes are a reflection of the activin A/Smad3 axis activation during LPS-induced inflammation, since they could not be observed in macrophages in the absence of LPS stimulation (Supplemental Figure 8, A–E).

A way for ATP to decrease inflammation is to be converted to adenosine by the ectonucleotidases CD39 and CD73, leading to the activation of the transcription factor CREB (26, 27). Notably, while the expression of *Entpd1* (encoding CD39) was increased in *Acvr1b*-Lyz2cre macrophages, the expression of *Nt5e* (encoding CD73) was dramatically decreased in both *Acvr1b*-Lyz2cre and *Smad3*-KO macrophages (Figure 4H and Supplemental Figure 7G). In addition, while *Acvr1b*-Lyz2cre or *Smad3*-KO macrophages treated with LPS reinforced their expression of *Arg1* and *Tgfbi* in the presence of ATP, this effect was totally abrogated when CD73 or CREB activities were inhibited (Figure 4, I and J). Furthermore, the same result was obtained in WT macrophages treated with follistatin (Supplemental Figure 7H). Finally, we observed increased expression of *Nt5e* in WT macrophages treated with a combination of LPS and activin A, which was completely abrogated in *Smad3*-KO macrophages (Supplemental Figure 7I). The data suggest that, in addition to its direct binding to the loci of several inflammatory genes, Smad3 also indirectly restricts inflammation by modulating metabolism of ATP and its degradation into adenosine by CD73, which consequently activates the transcription factor CREB (Supplemental Figure 7J).

### Activin A–mediated Smad3 activation suppresses sepsis.

**Figure 5:**
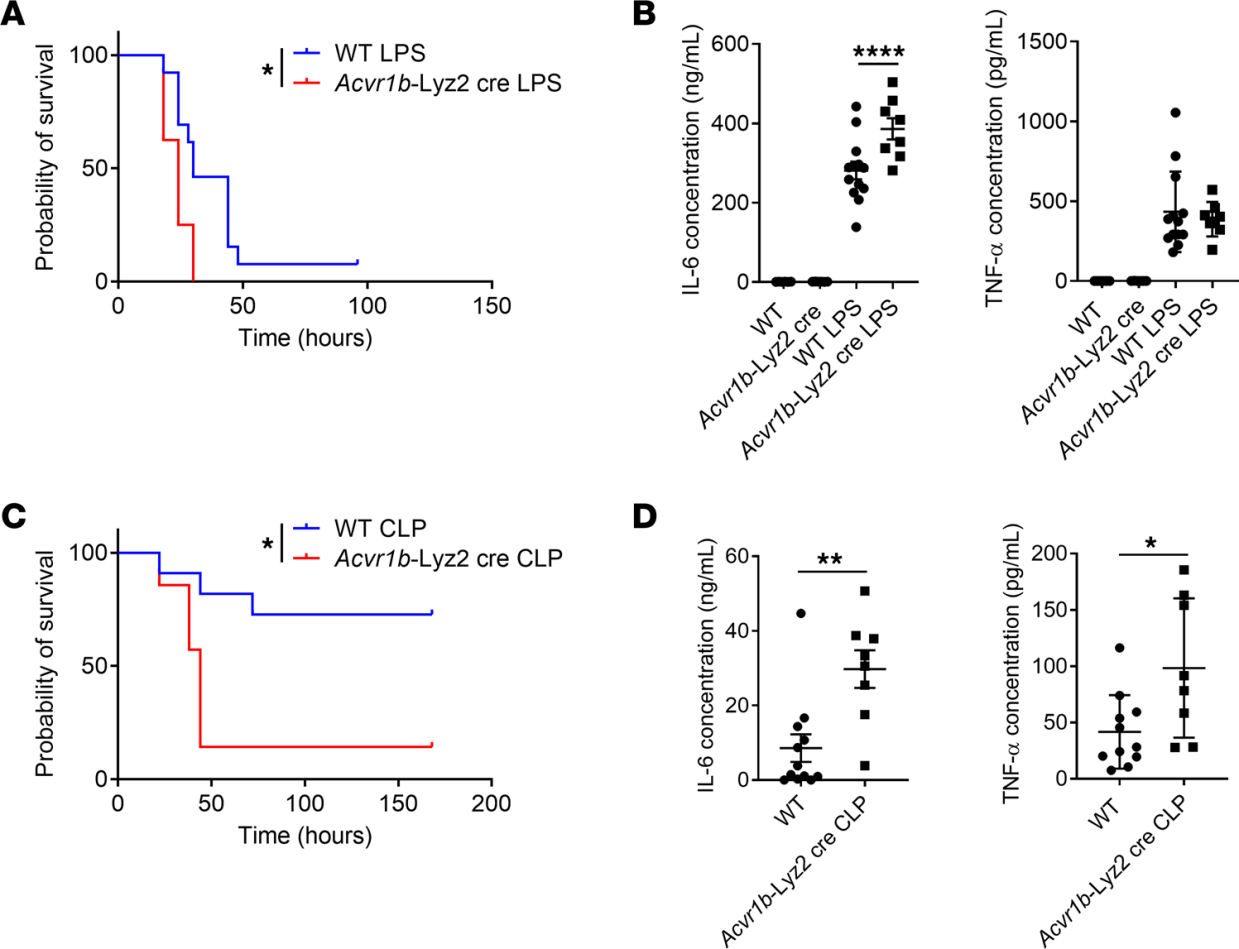
Activin A signaling in macrophages controls inflammation and survival during sepsis. (**A**) Survival of WT or *Acvr1b*-Lyz2cre mice injected i.p. with LPS. (*n* = 8–13.) (**B**) TNF-α and IL-6 levels in serum of WT or *Acvr1b*-Lyz2cre mice injected i.p. or not with LPS for 3 hours. (*n* = 6–13.) (**C**) Survival of WT or *Acvr1b*-Lyz2cre mice subjected to CLP surgery. (*n* = 7–11.) (**D**) TNF-α and IL-6 levels in serum of WT or *Acvr1b*-Lyz2cre mice subjected to CLP surgery. (*n* = 8–12.) Pooled from at least 2 independent experiments. *P < 0.05, **P < 0.01, ****P < 0.001 by log-rank (Mantel-Cox test, **A** and **C**) and Student’s *t* test (**B** and **D**).

Having elucidated that pSmad3C induced by LPS through the activin pathway acts as a negative regulator of macrophage activation in vitro and in vivo, we next investigated whether this regulated inflammation in mice. We first used LPS-induced endotoxin shock in mice. In this model, *Acvr1b*-Lyz2cre mice succumbed more and faster to the disease (Figure 5A). This was linked to a higher level of the inflammatory cytokine IL-6 (Figure 5B and Supplemental Figure 9A). The observation that IL-6 levels were already elevated early (1–3 hours) after LPS injection suggests that the heightened inflammation is unlikely to be caused by secondary activation of neutrophils (Supplemental Figure 9A). We next used a CLP-induced model of sepsis (in which real infection occurs) to confirm our findings. In this sepsis model, *Acvr1b*-Lyz2cre mice also had lower survival rates and increased levels of inflammation (Figure 5, C and D). Similarly, *Smad3*-KO mice also had lower survival rates after LPS injection or CLP surgery combined with higher levels of proinflammatory cytokines (Supplemental Figure 9, B–E), which is consistent with a previous report (28). Overall, Smad3 activation by activin A suppresses the development of sepsis in mice.

We then extended our studies to human patients to interrogate whether this activin A/pSmad3C axis was also activated in human patients with sepsis. We analyzed a cohort of sepsis patients that was already published in the Single Cell Portal from the Broad Institute (29). Interestingly, the expression of *Inhba*, *Smad3*, *Acvr1b*, *Acvr2a*, *Stat5a*, and *Stat5b* was all higher in the macrophages of patients compared with healthy volunteers (Supplemental Figure 9F), suggesting that the activin A/Smad3/STAT5 axis is also involved in human sepsis.

### SARS-CoV-2 viral E protein activates activin A/Smad3 pathway in macrophages.

Patients severely affected by SARS-CoV-2 infection develop a disease resembling sepsis in which the virus triggers activation of the innate immune system and generates inflammation (30, 31). We therefore hypothesized that the activin A/Smad3 pathway might be activated during inflammation induced by viral ligands such as SARS-CoV-2. To study this, we used the E protein from SARS-CoV-2, which has been described to be the mediator of inflammation during this viral infection in a TLR2-dependent pathway (32). We first took advantage of the fact that murine macrophages can be activated by E protein (12, 32) and stimulated macrophages in vitro with E protein to examine the expression of *Inhba* and the phosphorylation of Smad3. We found that E protein indeed induced a significant increase in activin A and the phosphorylation of Smad3C in an ACVR1B-dependent manner (Supplemental Figure 10, A and B). Importantly, human macrophages also exhibited higher levels of *INHBA* expression and increased pSMAD3C in an activin A–dependent manner in response to E protein challenge (Supplemental Figure 10, C and D). Similarly to LPS stimulation, E protein stimulation of WT macrophages in which activin A signaling was blocked or *Smad3*-KO macrophages resulted in much higher levels of inflammatory cytokines in comparison with WT macrophages (Supplemental Figure 10, E and F). These findings indicate that the activin A/Smad3 axis also restrains virus-induced inflammation and possibly sepsis, such as that occurring during COVID-19 infection.

### Activin A–mediated Smad3 activation regulates psoriatic inflammation.

**Figure 6:**
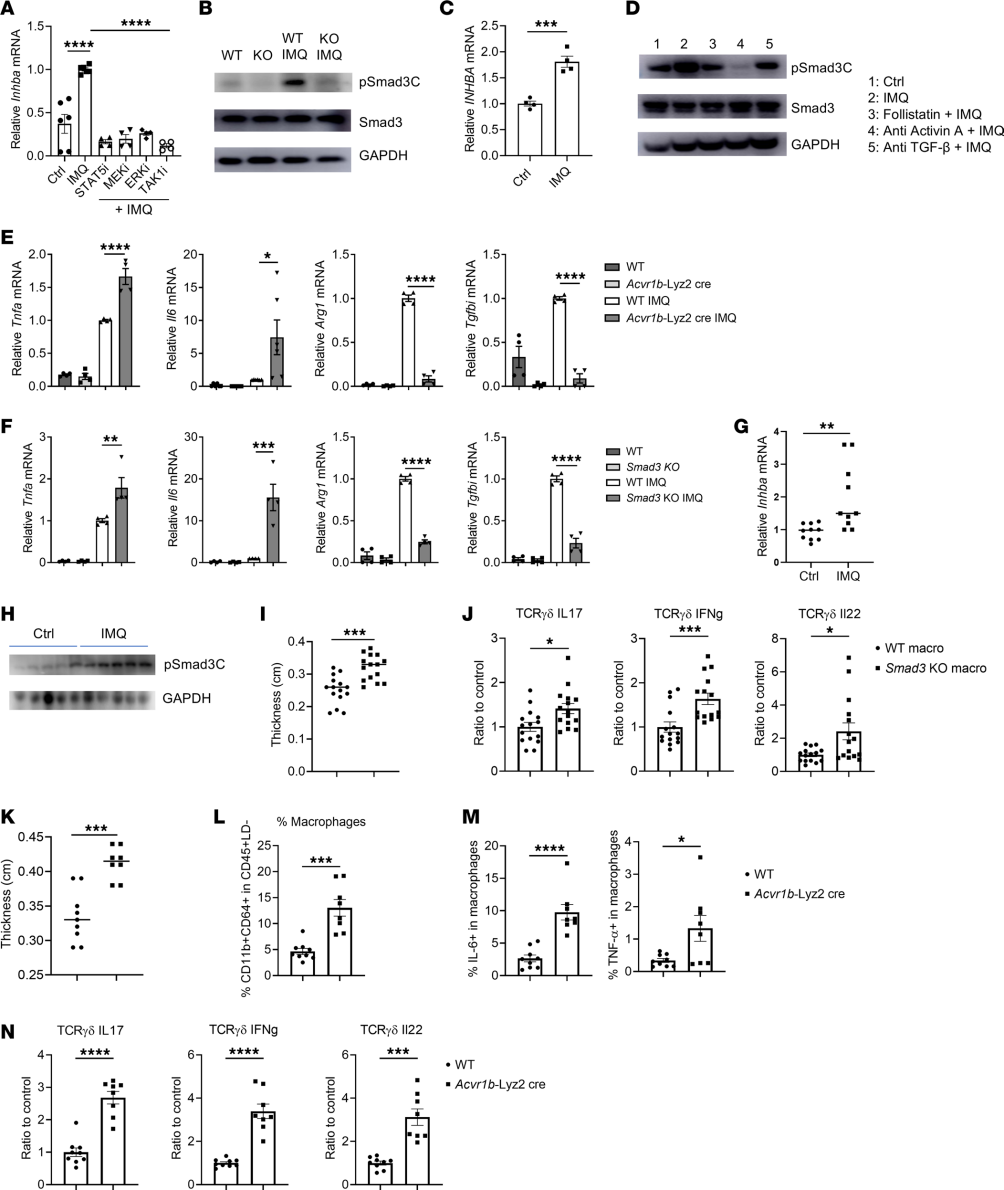
The activin A/Smad3 axis regulates inflammation during psoriasis. (**A**) RT-qPCR analysis of *Inhba* expression in macrophages pretreated for 1 hour with the indicated inhibitors and stimulated with imiquimod (IMQ) for 2 hours. (*n* = 4–6.) (**B**) Abundance of the indicated proteins in macrophages isolated from WT or *Acvr1b*-Lyz2cre mice (KO) and stimulated with IMQ for 6 hours. (**C**) RT-qPCR analysis of *INHBA* expression in human monocytes stimulated with IMQ for 2 hours. (*n* = 4.) (**D**) Abundance of the indicated proteins in human monocytes pretreated with follistatin or an anti–activin A or anti–TGF-β blocking antibody for 1 hour followed by IMQ stimulation for 6 hours. (**E**) RT-qPCR analysis of the indicated genes in macrophages from WT or *Acvr1b*-Lyz2cre mice stimulated or not with IMQ for 24 hours. (*n* = 4.) (**F**) RT-qPCR analysis of the indicated genes in macrophages from WT or *Smad3*-KO mice stimulated or not with IMQ for 24 hours. (*n* = 4.) (**G**) RT-qPCR analysis of *Inhba* expression in skin of WT mice treated with an IMQ topical application for 6 hours. (*n* = 10.) (**H**) Abundance of the indicated proteins in skin of WT mice treated with an IMQ topical application for 6 hours. Each band represents a mouse. Macrophages from WT or *Smad3*-KO macrophages were transferred intradermally in skin of CD45.1 WT mice followed by IMQ topical application for 6 consecutive days. Mice were then harvested and analyzed. (**I**) Skin thickness. (*n* = 15.) (**J**) TCRγδ cytokine production in skin. (*n* = 15.) WT or *Acvr1b*-Lyz2cre mice were treated with IMQ topical application for 6 consecutive days, then harvested and analyzed. (**K**) Skin thickness. (**L**) Macrophage frequency in skin. (**M**) Production of cytokines by macrophages. (**N**) TCRγδ cytokine production in skin. (**K**–**N**, *n* = 8–9.) Representative or pooled from at least 2 independent experiments. *P < 0.05, **P < 0.01, ***P < 0.005, ****P < 0.001 by Student’s *t* test (**C** and **G**–**N**) or 1-way ANOVA (**A**, **E**, and **F**).

We observed that pathogen-associated molecular patterns (e.g., bacterial LPS and viral E protein) that can be sensed in the extracellular environment by plasma membrane receptors (TLR4 and TLR2, respectively) trigger the production of activin A and consequent activation of Smad3 to restrain overt inflammation. However, whether TLR ligands that signal through endosomal receptors could do the same remains poorly understood. We therefore used the TLR7 ligand imiquimod (IMQ) to test whether that was the case. We observed that, similarly to LPS, IMQ promoted *Inhba* expression in a manner dependent on TAK, MEK, ERK, and STAT5 as well as TLR7 and MyD88 in macrophages in culture (Figure 6A and Supplemental Figure 11A). Similarly, IMQ induced pSmad3C in an activin A–dependent manner, since deletion of *Acvr1b* in macrophages abrogated the effect of IMQ (Figure 6B). Importantly, the same results were obtained in human monocytes (Figure 6, C and D): IMQ enhanced *INHBA* expression and pSmad3C, and blockade of activin A signaling abolished the IMQ effects. As expected, *Smad3*-KO and *Acvr1b*-Lyz2cre macrophages were hyperinflammatory upon IMQ treatment in vitro (Figure 6, E and F, and Supplemental Figure 11B).

We next used the IMQ-induced psoriasis model in mice (33) to study the role of the activin A/Smad3 pathway in macrophages in the regulation of the disease. We observed that, 6 hours after IMQ application on the skin, the levels of *Inhba* and pSmad3C were significantly increased in the skin tissue (Figure 6, G and H).

We then interrogated the role of this signaling cascade in the development of psoriasis. To avoid any potential impact on other cells expressing Smad3, we intradermally injected WT or *Smad3*-KO macrophages (CD45.2^+^) into the back skin of CD45.1 WT mice followed by IMQ application daily on the skin for 6 days. Transfer of *Smad3*-KO macrophages had a deleterious effect on the disease, marked by an increase in skin thickness and an increased production of proinflammatory cytokines by γδ T cells, which are the main drivers of the disease (Figure 6, I and J, and Supplemental Figures 11C and 12) (33). To provide further evidence that *Smad3*-deficient macrophages could be inflammatory in an endogenous context as well upon IMQ treatment, we generated bone marrow (BM) chimeras by reconstituting lethally irradiated CD45.1 mice with BM from CD45.2 WT or *Smad3*-KO mice. We observed an almost complete reconstitution of the immune system in the blood (Supplemental Figure 11D) and reconstitution of about 60% of macrophages in the skin of both WT and *Smad3*-KO BM chimeras (Supplemental Figure 11E). Interestingly, upon IMQ application, *Smad3*-KO BM chimeras had exacerbated psoriasis development as exemplified by increased skin thickness (Supplemental Figure 11, F and G). This was associated with an increased ability of the reconstituted macrophages to produce IL-6 (Supplemental Figure 11H), confirming that *Smad3*-deficient macrophages are indeed proinflammatory in the context of psoriasis.

In addition, *Acvr1b*-Lyz2cre mice also developed more severe disease compared with WT mice (Figure 6K and Supplemental Figure 11I), which was associated with an increased infiltration of macrophages in the skin, and more IL-6– and TNF-α–producing macrophages (Figure 6, L and M). γδ T cells also produced more proinflammatory cytokines (e.g., IL-17A, IL-22, and IFN-γ) in *Acvr1b*-Lyz2cre mice (Figure 6N).

We then deciphered how macrophages regulated the disease development and γδ T cell activation. Since *Acvr1b*-Lyz2cre macrophages produced more proinflammatory cytokines, we hypothesized that the activin A/Smad3 pathway in macrophages might regulate γδ T cell activation and the disease by regulation of cytokine production in macrophages. We thus blocked the proinflammatory cytokines TNF-α, IL-6, IL-1β, and IL-23A, which are known to be crucial to γδ T cell activation, in WT and *Acvr1b*-Lyz2cre mice during psoriasis. We observed that the increased skin thickness and γδ T cell activation in *Acvr1b*-Lyz2cre mice were abrogated when these proinflammatory cytokines were blocked (Supplemental Figure 11J). The data indicate that activin A–mediated Smad3 phosphorylation also restrains macrophage activation during psoriatic inflammation in the skin, notably via its ability to restrain the generation of inflammatory γδ T cells.

Finally, we tested whether other TLR ligands could also activate the activin A/pSmad3C pathway. Macrophages treated for 6 hours with the TLR9 ligand CpG, the TLR7/8 ligand R848, and the TLR2 ligand Pam3CSK4 all demonstrated induction of pSmad3C and *Inhba* expression (Supplemental Figure 11, K and L), demonstrating that the activin A/Smad3 axis can be activated by several TLR ligands.

## Discussion

In this study, we identified the activin A/pSmad3C axis as a natural brake to inflammation put in place by macrophages to prevent their overt activation. Importantly, this axis is activated by a variety of stimuli, including bacterial and viral ligands, as well as in the context of autoimmunity (Supplemental Figure 15).

TGF-β has been demonstrated to be an important molecule to control inflammation in immune cells, including macrophages (12, 34–36). It is generally believed that pSmad3C is a marker of TGF-β signaling activation. Intriguingly, we observed that LPS, E protein, and IMQ all induce pSmad3C in macrophages, which is independent of TGF-β signaling. Instead, Smad3 is phosphorylated by an autocrine activin A–dependent loop, and therefore is dependent on the activin A receptors (especially ACVR1B) in macrophages. This is of utmost importance since TGF-β and activin A, besides their effects on Smad3, might have different functions and regulations, especially in the context of diseases. For example, it has been reported in CD4^+^ T cells that activin A drives the generation of pathogenic Th17 cells in the context of neuroinflammation but that TGF-β was unable to do it (37). In the context of sepsis and COVID-19, TGF-β appears to have a deleterious role (12, 38, 39). Moreover, during psoriasis, the overexpression of TGF-β in the epidermis leads to the development of psoriasis-like skin inflammation (40), overall demonstrating a divergent function between TGF-β and activin A in these diseases. Our findings in this study have paved what we believe to be a new way to further understand how these two molecules differentially regulate innate immune responses and how to control them during diseases.

The role of activin A in modulating macrophage functions is still unclear. Monocytes and other leukocytes have been shown to produce activin A in response to LPS and in pediatric sepsis patients, which was demonstrated to suppress the expression of inflammatory cytokines when added exogenously (41, 42). Some other reports, however, have suggested that activin A has a proinflammatory effect on macrophages (43, 44). Notably, Jones et al. reported that activin A could be induced in the serum of mice after LPS treatment (43), and systemic treatment with follistatin (to block activin A) increased the survival of the mice. However, we clearly demonstrate here that genetic deletion of the activin A receptor in macrophages is detrimental during sepsis. This may indicate that activin A could have a different effect on macrophages compared with other cells. It also suggests that follistatin could have additional roles besides inhibiting activin A. Moreover, the mechanisms by which activin A production is regulated, as well as its downstream signaling and its detailed effect on macrophage phenotype and disease development, remained poorly understood. Here, by using a combination of blocking antibody, follistatin, and, most importantly, conditional deletion of the activin A receptor *Acvr1b* in macrophages, we studied in depth the role of the activin A/Smad3 axis. We demonstrated that activin A is naturally produced by macrophages, triggered by the activation of STAT5 in response to various inflammatory contexts. Activin A can therefore signal through Smad3 to pose a natural brake to innate inflammation. Mechanistically, we demonstrated that this axis is critical to regulate macrophage ATP metabolism, which helps avoid uncontrolled levels of inflammation in macrophages. Importantly, this axis protects mice against the development of overt inflammation in models of sepsis, viral infection, and psoriasis, demonstrating a generalized mechanism, and suggesting that promoting the activin A/Smad3 pathway could provide a therapeutic strategy in these diseases. Indeed, we also demonstrated that this axis was active in humans.

LPS regulates the inflammatory response by using a wide variety of mechanisms, including metabolic reprogramming. It is now well appreciated that LPS promotes the induction of glycolysis while it inhibits mitochondrial metabolism (breaking the TCA cycle and decreasing oxidative phosphorylation) (22, 23, 45). Nevertheless, the mechanisms regulating these changes and how a decreased mitochondrial metabolism regulates inflammation remain poorly understood. Here, we have revealed that the activin A/Smad3 axis supports the generation of mitochondria and the maintenance of ATP production at homeostatic levels. In parallel, ATP is also converted into adenosine by CD73, which can enforce the effect of Smad3 in regulating the expression of antiinflammatory molecules (such as *Arg1* and *Tgfbi*) in a CREB-dependent manner. This is in line with the notion that CD73 and CREB can promote an antiinflammatory phenotype in macrophages (26, 27), and deciphers a mechanism by which the regulation of ATP metabolism by LPS regulates inflammation. Although our RNA-Seq data suggest that Smad3 regulates mitochondrial biogenesis by transcriptional regulation of several genes involved in mitochondrial function/biogenesis, future studies are needed to unravel the exact mechanisms by which Smad3 regulates mitochondrial biogenesis as well as how and to what extent the CD73/CREB axis controls the inflammatory response to inflammatory stimuli.

In summary, we have demonstrated that, in proinflammatory contexts, macrophages activate pSmad3C in an activin A–dependent, TGF-β–independent manner. This axis naturally protects macrophages against overt inflammatory responses and metabolic dysfunction in several pathogenic contexts, including sepsis, COVID-19, and psoriasis. As the activin A/Smad3 axis is conserved in human macrophages, it may be targeted to harness new therapeutic strategies during infections and autoimmunity.

## Methods

### Sex as a biological variable.

Our study examined male and female animals, and similar findings are reported for both sexes. For sepsis experiments, because males are less susceptible to disease development, we only used female mice.

### Mice.

C57BL/6 mice were obtained from The Jackson laboratory. *Smad3*-KO mice and RI-Lyz2cre mice were obtained as described previously (12, 46). *Acvr1b*-Lyz2cre mice were generated by crossing of *Acvr1b^fl/fl^* mice (20) with Lyz2cre mice. *Traf6*- and *Stat5*-Lyz2cre mice were generated by crossing of *Traf6^fl/fl^* and *Stat5^fl/fl^* mice with Lyz2cre mice (both obtained from The Jackson Laboratory). *Tlr4*-, *Tlr7*-, *MyD88*-, and *Trif*-KO mice were obtained from The Jackson Laboratory. Mice were bred under specific pathogen–free conditions in the animal facility of the National Institute of Dental and Craniofacial Research (NIDCR).

### Human samples.

For the generation of human macrophages, cells from healthy donors were obtained from the NIH Blood Bank (Bethesda, Maryland, USA). Monocytes were isolated by elutriation (by the NIDCR Combined Technical Research Core facility) and were differentiated for 7 days in the presence of 10% fetal bovine serum in RPMI medium (Thermo Fisher Scientific) supplemented with antibiotics (penicillin and streptomycin), sodium pyruvate, and glutamine (all from Gibco) but without fetal bovine serum.

### Cell culture.

Mouse peritoneal macrophages and BM-derived macrophages were generated as described before (12). The cells were then cultured in RPMI medium containing antibiotics (penicillin and streptomycin), sodium pyruvate, and glutamine. The cells were treated with LPS (10 ng/mL; Sigma Aldrich), activin A (100 ng/mL; R&D Systems), TGF-β (5 ng/mL; PeproTech), E protein (1 μg/mL; ABclonal), imiquimod (1 μg/mL; InvivoGen), ATP (20 μM; Cayman Chemical), CpG ODN1826 (1 μM; InvivoGen), R848 (1 μg/mL; InvivoGen), or PamCysK (100 ng/mL; InvivoGen). Macrophages were pretreated for 1 hour before these treatments with follistatin (0.5 μg/mL; BioLegend), cycloheximide (5 μM; Cayman Chemical), anti–TGF-β antibody (50 μg/mL; Bio X Cell), anti–activin A antibody (2 μg/mL; R&D Systems), Smad3 inhibitor (SIS3, 2 μM; Cayman Chemical), TAK1 inhibitor (5 nM; Sigma-Aldrich), MEK inhibitor (10 μM; Cayman Chemical), ERK inhibitor (1 μM; Cayman Chemical), STAT5 inhibitor (100 μM; Cayman Chemical), CD73 inhibitor (10 μM; Cayman Chemical), and CREB inhibitor (1 μM; Cayman Chemical).

### Western blotting.

Tissue lysates from macrophages were prepared in NP-40 lysis buffer (1% NP-40, 20 mM Tris [pH 7.5], 150 mM NaCl, 2.5 mM EDTA, 10 mM NaF, 10 mM NaPPi [sodium pyrophosphate], 10 mM PMSF, 0.25% Na deoxycholate, 1 mM Na_3_VO_4_, and 5 μg/mL of each of the proteinase inhibitors aprotinin, leupeptin, and pepstatin A, all from Sigma-Aldrich except PMSF from Fluka Biochemika). Protein samples were separated on 10% Tris-glycine gels (Thermo Fisher Scientific) and transferred to PVDF membranes (Thermo Fisher Scientific). The membranes were soaked in blocking buffer (5% milk; Bio-Rad) for 1 hour at room temperature and subsequently incubated with the appropriate primary antibodies overnight at 4°C. The next day, the membranes were washed and incubated for 1 hour at room temperature with HRP-conjugated secondary antibodies (Cell Signaling Technology). Immunoreactivity was detected using ECL, and images were acquired with an Amersham Imager 600 (General Electric) followed by stripping of the membranes with Restore Plus Western blot stripping buffer (Thermo Fisher Scientific) and incubation with GAPDH antibody (Sigma-Aldrich) as a control. Data were quantified using ImageJ (NIH).

### RT-qPCR.

RNA from cells was extracted using an RNeasy Plus Micro kit (QIAGEN) following the manufacturer’s recommendations. cDNA was synthesized using a High Capacity cDNA Reverse Transcription Kit (Thermo Fisher Scientific). qPCR was performed using TaqMan Master Mix (Thermo Fisher Scientific). The primers used are listed in Supplemental Table 1. Total transcript values were normalized using mouse or human *Hprt*. Results were calculated using the comparative ΔΔCt method (47). Results are shown as fold change in comparison with control.

### ELISA.

The levels of TNF-α, IL-6 (BioLegend), activin A (R&D Systems), and ATP (Cayman Chemical) were measured in the supernatant and the serum by ELISA according to the manufacturers’ recommendations.

### Detection of TGF-β signaling with MFB-F11 reporter cells.

Detection of TGF-β signaling with MFB-F11 reporter cells was performed as previously described (18, 19). Briefly, MFB-F11 cells (fibroblast cell line isolated from mouse *Tgfb1^–/–^* embryos [MFB] stably transfected with the SBE-SEAP reporter) were seeded at a density of 30,000 cells per well in 96-well flat-bottom tissue culture plates. After an overnight incubation, the cells were washed with PBS followed by addition of 50 μL of serum-free DMEM supplemented with penicillin and streptomycin (DMEM/P/S) and 1× B27 supplement (test medium) for 2 hours. The individual samples, which included treatments with recombinant TGF-β1 in the presence of the neutralizing anti–TGF-β antibody 1D11 or an isotype control, were then prepared in a final volume of 50 μL of test medium and added to the cells for a final volume of 100 μL. The cells were incubated for 18–24 hours, after which the supernatants were collected and stored at –20°C. The induction of secreted alkaline phosphatase (SEAP) was measured in the collected supernatants with the Great EscAPe SEAP Chemiluminescence Kit (Promega) as previously described (18, 19).

### CHIP assay.

The iDeal ChIP-qPCR kit (Diagenode) was used according to the manufacturer’s instructions to perform ChIP experiments. Four million cultured macrophages with or without LPS during 2 hours were used per condition. An equal amount of processed chromatin was used as an input control or was incubated with an anti–c-STAT5 antibody (Abcam) or its isotype-matched control antibody (rabbit IgG, Abcam). Immunoprecipitated DNA and total input DNA were analyzed with a SYBR Green Supermix kit (Thermo Fisher Scientific). Results after immunoprecipitation were normalized with the input and IgG. The sequence of primers is provided in Supplemental Table 2.

### FACS and immunofluorescence staining.

Cells were stained with the Zombie Yellow Fixable Viability Kit (BioLegend) for 10 minutes at 4°C followed by surface staining with anti-mouse antibodies (CD45 for immune cells, CD11b and CD64 for macrophages, TCRβ and TCRγδ for γδ T cells) for 20 minutes at 4°C in the presence of Fcγ receptor–blocking antibodies. Intracellular staining was performed using the Perm/Wash buffer set (BD Biosciences) for 20 minutes at 4°C followed by staining with anti-mouse antibodies (IFN-γ, IL-17A, IL-22, IL-6, and TNF-α). For cytokine staining, cells were stimulated for 4 hours at 37°C with PMA (5 ng/mL), ionomycin (1 μg/mL), and Golgi-Plug (1:1,000 dilution; BD Biosciences). Cells were analyzed on a BD LSRFortessa analyzer.

For analysis of pSmad2/3C by flow cytometry, peritoneal cells were fixed using 4% paraformaldehyde for 20 minutes at 37°C followed by a PBS wash. Cells were then permeabilized with 90% methanol overnight at –20°C, followed by 2 washes with PBS. Cells were then stained with pSmad2/3C antibody (Cell Signaling Technology) for 45 minutes at 4°C followed by a wash and staining with an anti-rabbit Alexa Fluor 488 secondary antibody (Thermo Fisher Scientific) for 45 minutes at 4°C with anti-Ly6G, F4/80, CD11c, CD3, and CD19 to identify neutrophils, macrophages, dendritic cells, T cells, and B cells.

For mitochondrial staining, macrophages were stained at 37°C with TMRM (50 nM for 30 minutes), MitoSOX Red (5 μM for 10 minutes), and MitoTracker Green (100 nM for 30 minutes) (Thermo Fisher Scientific) in RPMI medium. For immunofluorescence, cells were further permeabilized with methanol for 15 minutes at 4°C, washed, and stained with DAPI for 5 minutes (1 μg/mL; Thermo Fisher Scientific, 62247) before mounting on slides and images were acquired with a Nikon A1R+ MP microscope. Data were quantified using ImageJ.

### RNA sequencing.

Total RNA was reverse-transcribed by SuperScript IV (Invitrogen) using template-switching oligonucleotide and oligo-dT primers followed by amplification of the second-strand cDNA with LongAmp Taq polymerase (New England Biolabs). Libraries were prepared using the Nextera XT method (Illumina) kit, individually barcoded, and sequenced on a NextSeq 2000 instrument (Illumina) using 100 × 100 paired-end mode. The FASTQ files were aligned to the mouse genome (GRCm38) using vM11 annotation and gene counts generated using STAR (v2.7.3a) (Github). An expression matrix of raw gene counts was filtered to remove low-count genes (defined as those with fewer than 5 reads in at least 1 sample). The filtered expression matrix was analyzed in DESeq2 (Bioconductor) to find differentially expressed genes (48).

### Sepsis models.

Mice were injected with 15 mg/kg of LPS from *E*. *coli* (Sigma-Aldrich). Survival rates were monitored for 96 hours, and serum was extracted, 3 hours after LPS injection, from the blood, followed by centrifugation for 20 minutes at room temperature. Mice defined as “WT” were *Acvr1b^+/+^*-Lyz2cre^+^ littermates (as opposed to *Acvr1b*-Lyz2cre mice, which were *Acvr1b^fl/fl^*-Lyz2cre^+^).

For the CLP-induced sepsis model (12), mice were subjected to a midline laparotomy followed by a ligation of approximately 50% of the cecum to induce sepsis. A single through-and-through puncture with a 19 G needle was then made distal to the ligature. Survival rates were monitored for 7 days, and serum was harvested at 18 hours.

### Psoriasis model.

62.5 mg of imiquimod (IMQ) (or Vaseline, Unilever, as a control) was applied to the back of shaved mice for 6 consecutive days. Tissues were homogenized using 2.0 mm zirconia beads (Biospec) and Trizol reagent according to the manufacturer’s instructions (QIAGEN). For Western blotting, tissue was homogenized using T-PER buffer (Thermo Fisher Scientific) supplemented with protease inhibitor (cOmplete Mini, Sigma-Aldrich) and phosphatase inhibitor (PhosSTOP, Roche). Cells were extracted by cutting and incubation of the skin at 37°C in 500 μg/mL of Liberase DH (Roche) dissolved in HBSS for 1 hour. After incubation, the skin was smashed through a 70 μm strainer and filtered a second time before FACS staining as described above. For the macrophage transfer experiments, 0.5 million thioglycolate-elicited macrophages, isolated from WT or *Smad3*-KO mice, were injected intradermally (in 2 sites) in CD45.1 mice right before the first IMQ application. For the blocking antibody experiment, anti–TNF-α, –IL-6, –IL-1β, and –IL-23A antibodies (or IgG control; 100 μg of each antibody or 400 μg of IgG control) were injected i.p. 24 hours before the first IMQ application and again at day 4. A list of all the antibodies used in this study is provided in Supplemental Table 3. For histology, organs were fixed in 10% formalin (Thermo Fisher Scientific), paraffin-embedded, and cut in 4-μm sections. Tissues were stained with hematoxylin and eosin, and acquisition was performed using a NanoZoomer S60 scanner (Hamamatsu). For the BM chimeras, CD45.1 mice were lethally irradiated (9.50 Gy) before injection of 5 million cells from CD45.2 WT or *Smad3*-KO BM 5 hours after irradiation. Trimethoprim-sulfamethoxazole was given for 2 weeks, and autoclaved cages were used to house the animals. Four weeks after reconstitution, IMQ was applied for 6 days as described above.

### Statistics.

Statistical analyses were performed using GraphPad Prism 8 software. Data are presented as mean ± SEM. Statistical significance (*P* < 0.05) was determined by unpaired *t* test (2-tailed, 2 groups), 1-way analysis of variance (ANOVA) (more than 2 groups), or log-rank (Mantel-Cox) test (survival curve). Identified outliers were excluded. Statistical analysis was performed in all the required experiments. All experiments were performed at least twice independently.

### Study approval.

Animal studies were performed according to National Institutes of Health (NIH) guidelines and approved by the NIDCR Animal Care and Use Committee. Human studies were approved by the US NIH through their protocol number NCT000001846 (Department of Transfusion Medicine).

### Data availability.

All data underlying the figures are provided in the Supporting Data Values file. RNA-Seq data were deposited in the NCBI’s Gene Expression Omnibus database (GEO GSE284154).

## Author contributions

TG conceived the research, designed and performed the experiments, analyzed data, and drafted the manuscript. YJL, WJ, NL, LCP, Weiwei Chen, JW, and GD performed and analyzed experiments. DM and RJM analyzed the RNA-Seq data. GHS provided the *Acvr1b*-floxed mice. WanJun Chen conceived and supervised the research, designed the experiments, and wrote the manuscript.

## Supplementary Material

Supplemental data

Unedited blot and gel images

Supplemental tables 1-3

Supporting data values

## Version 1 03/11/2025

In-Press Preview

## Version 2 05/01/2025

Electronic publication
