# Supplementary material for: Activin A activation of Smad3 mitigates innate inflammation in mouse models of psoriasis and sepsis
Source: J Clin Invest. 2025 Mar 11;135(9):e187063. doi: 10.1172/JCI187063 (PMC12043092; doi:10.1172/JCI187063)
Supplement: Supplemental data [file jci-135-187063-s254.pdf]

Figure S1 LPS activates Smad3 in a TGF-β independent manner

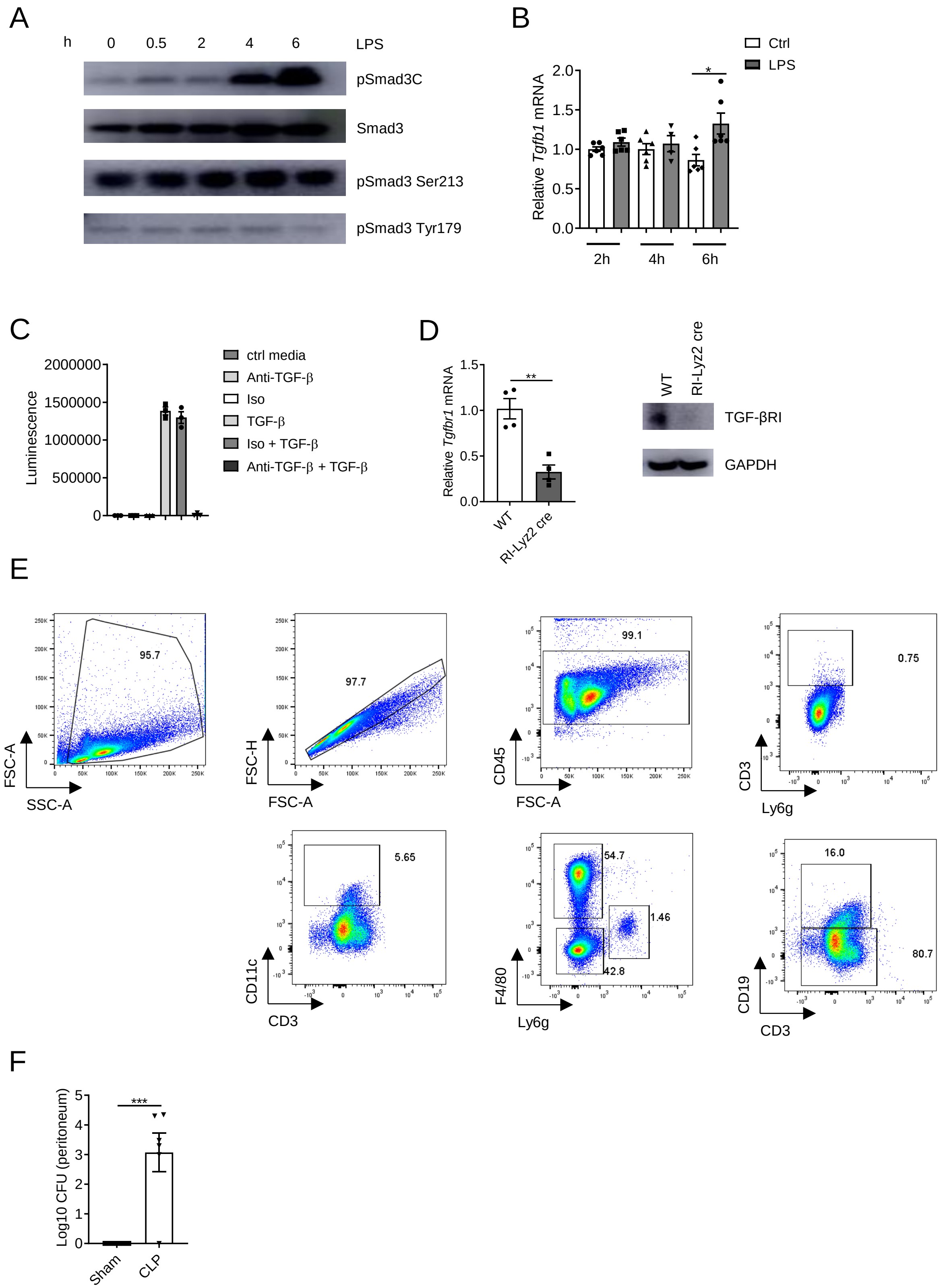

Figure S2 Gene expression of TGF-β superfamily members

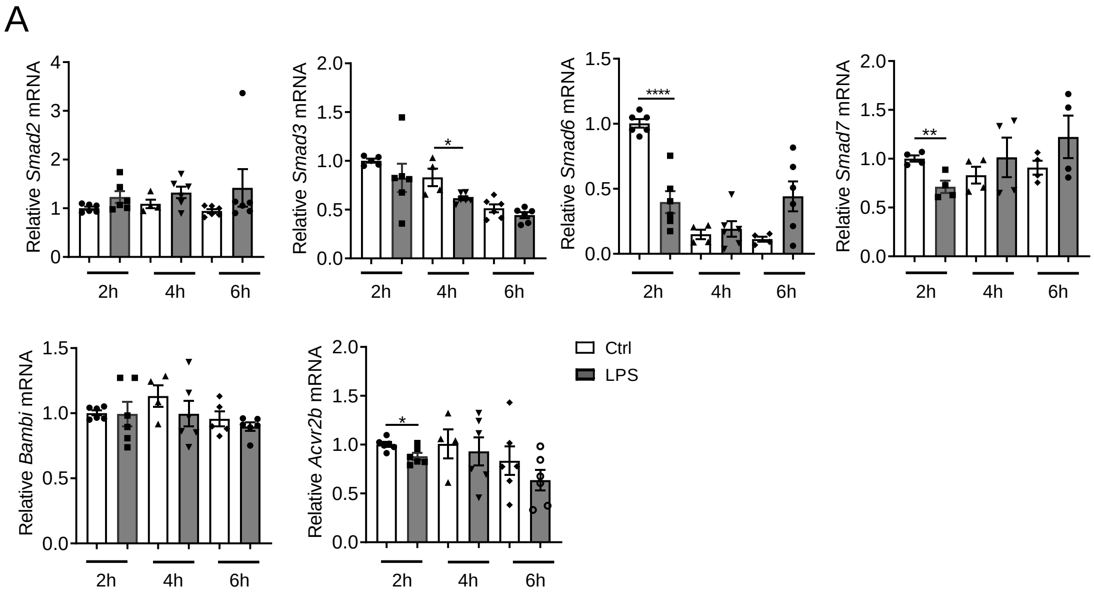

Figure S3 LPS phosphorylates Smad3 in an Activin A dependent manner

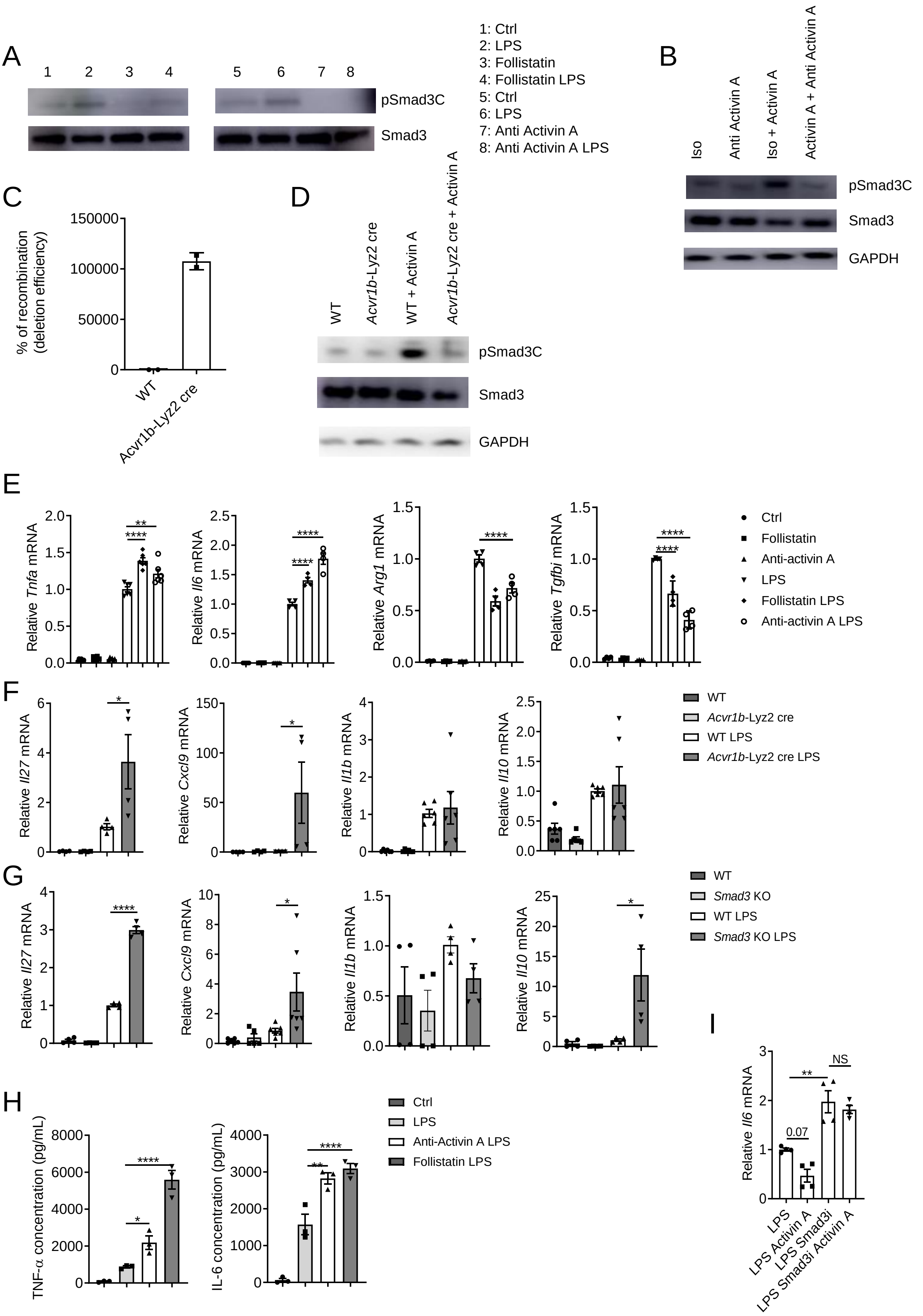

Figure S4 LPS induces Activin A expression through a TLR4-Myd88-MAPK-STAT5 pathway

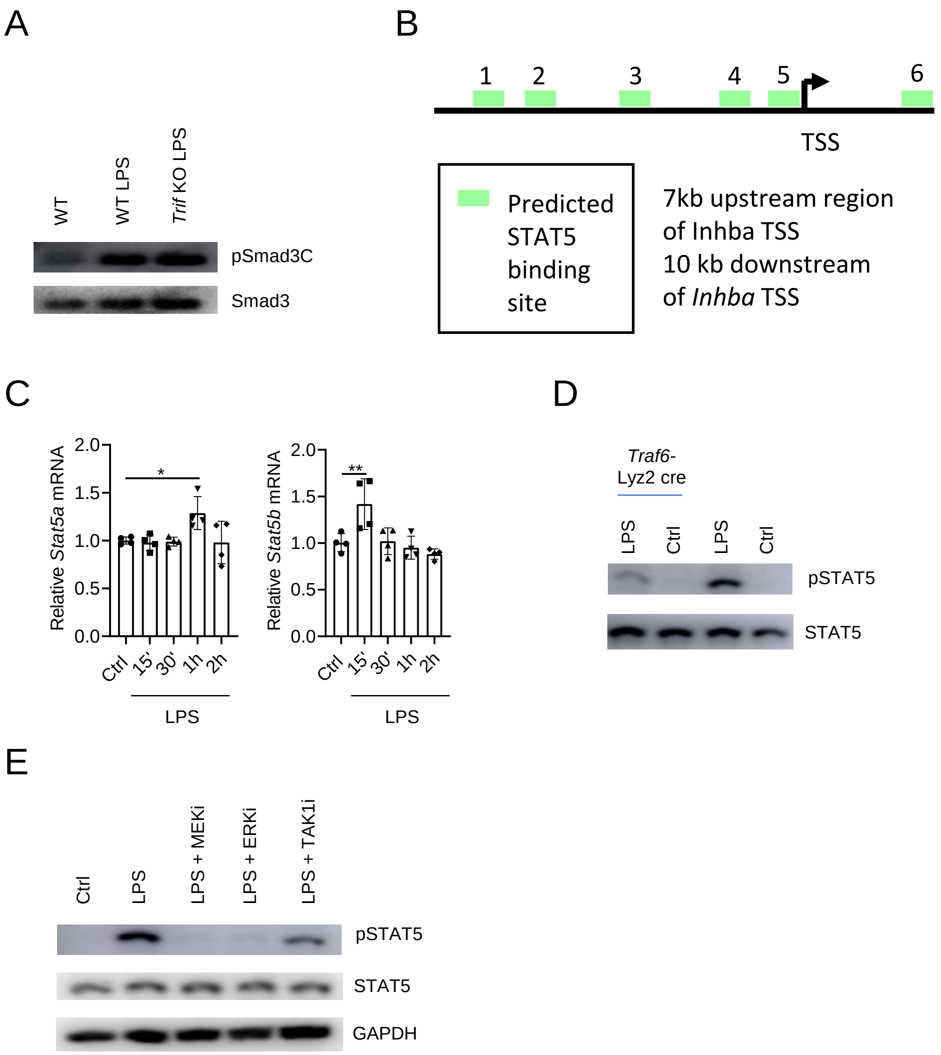

Figure S5 Validation of knock-out macrophages and inhibitors

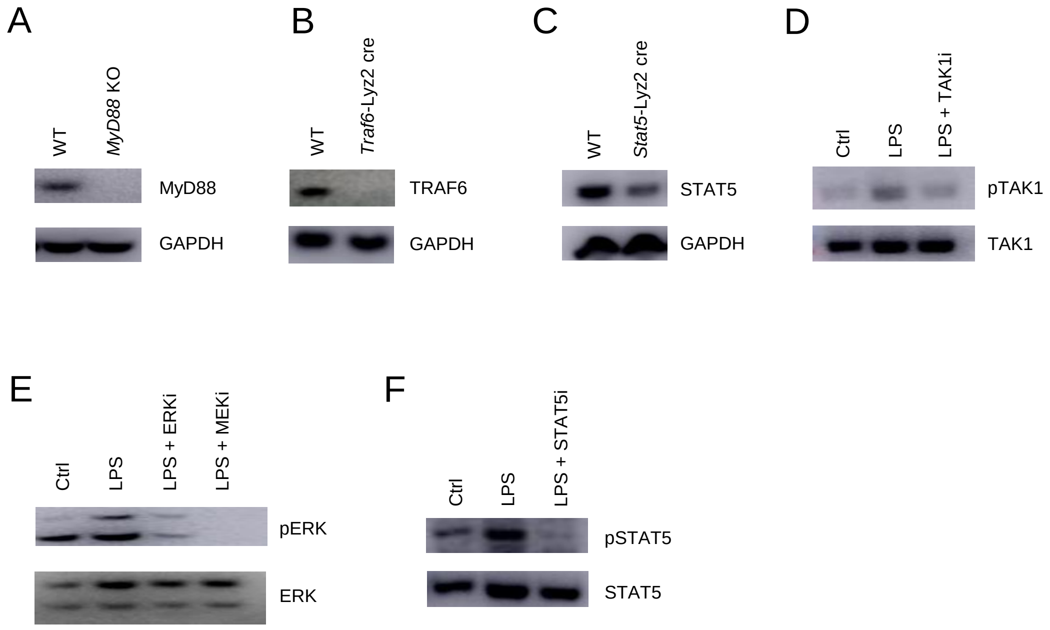

Figure S6 RNAseq of *Smad3* deficient macrophages

A

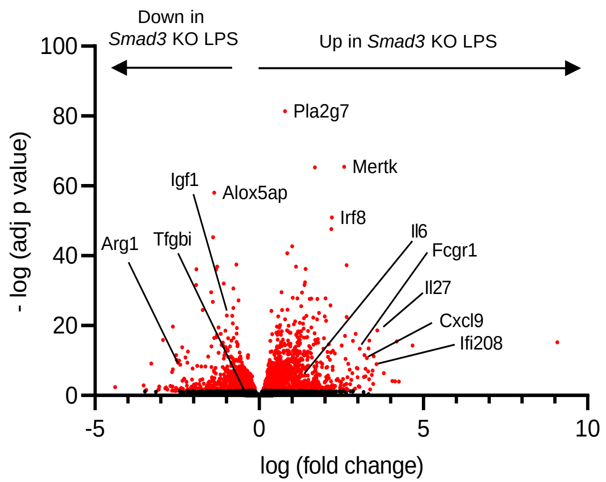

B

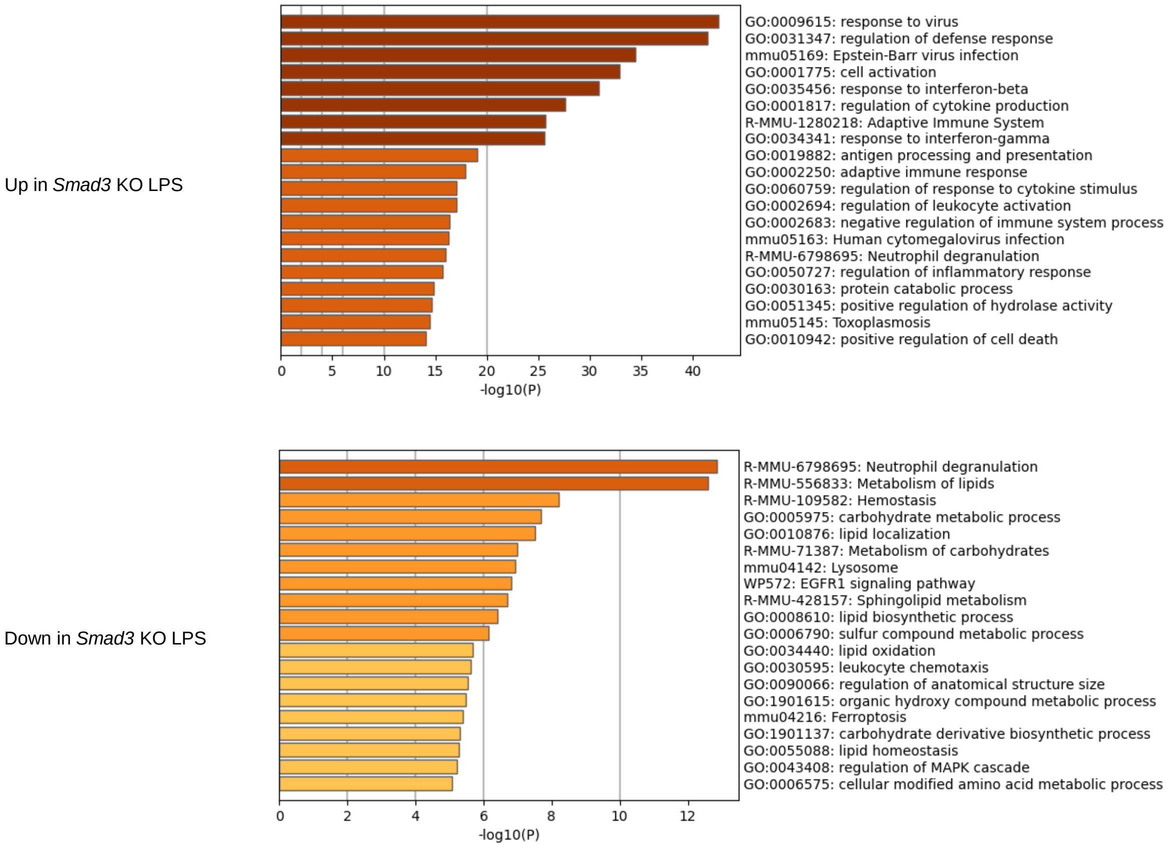

Figure S7 The Activin A-Smad3 pathway regulates macrophage ATP metabolism during inflammation

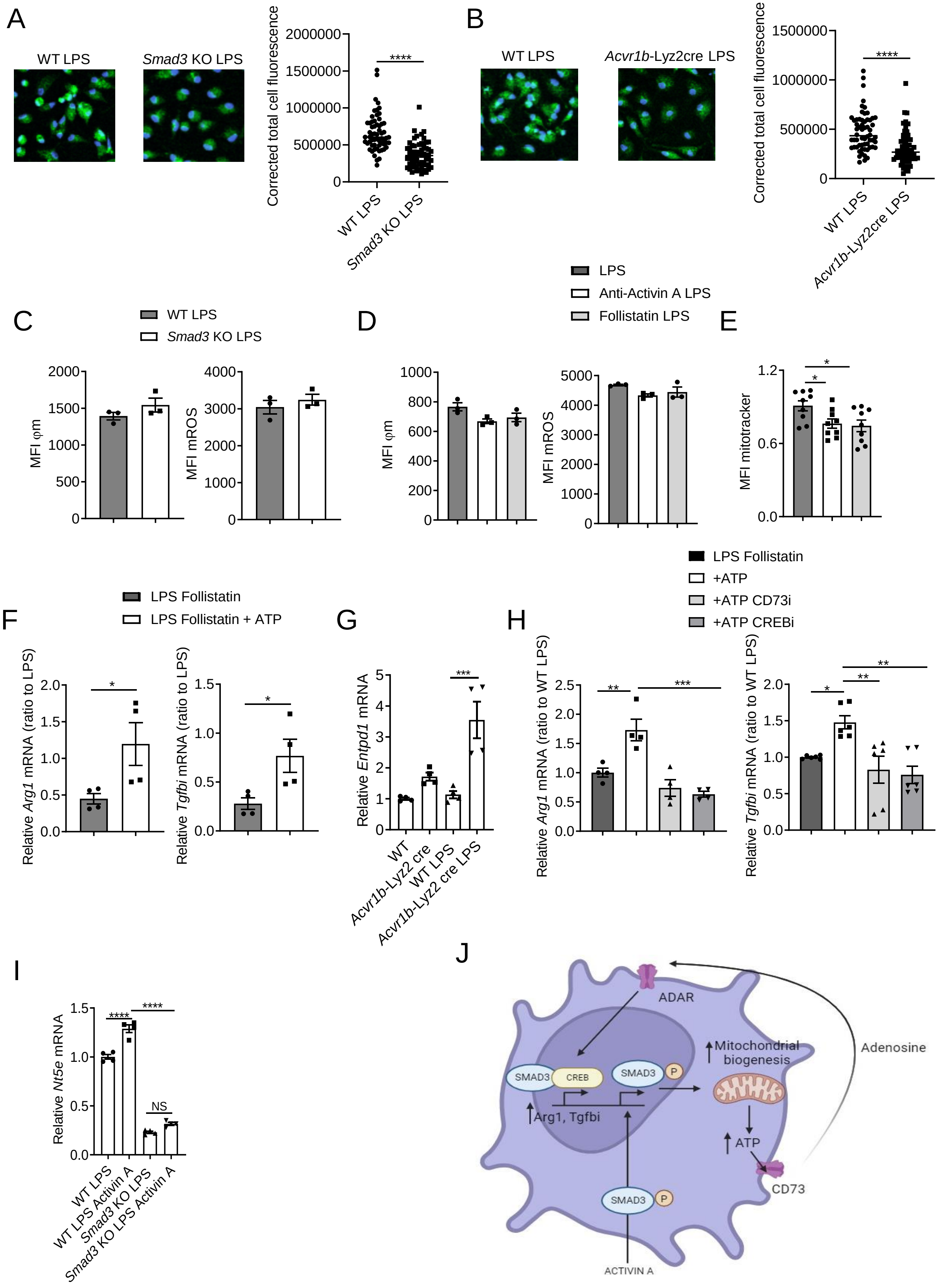

Figure S8 The Activin A-Smad3 pathway regulates macrophage ATP metabolism during inflammation (2)

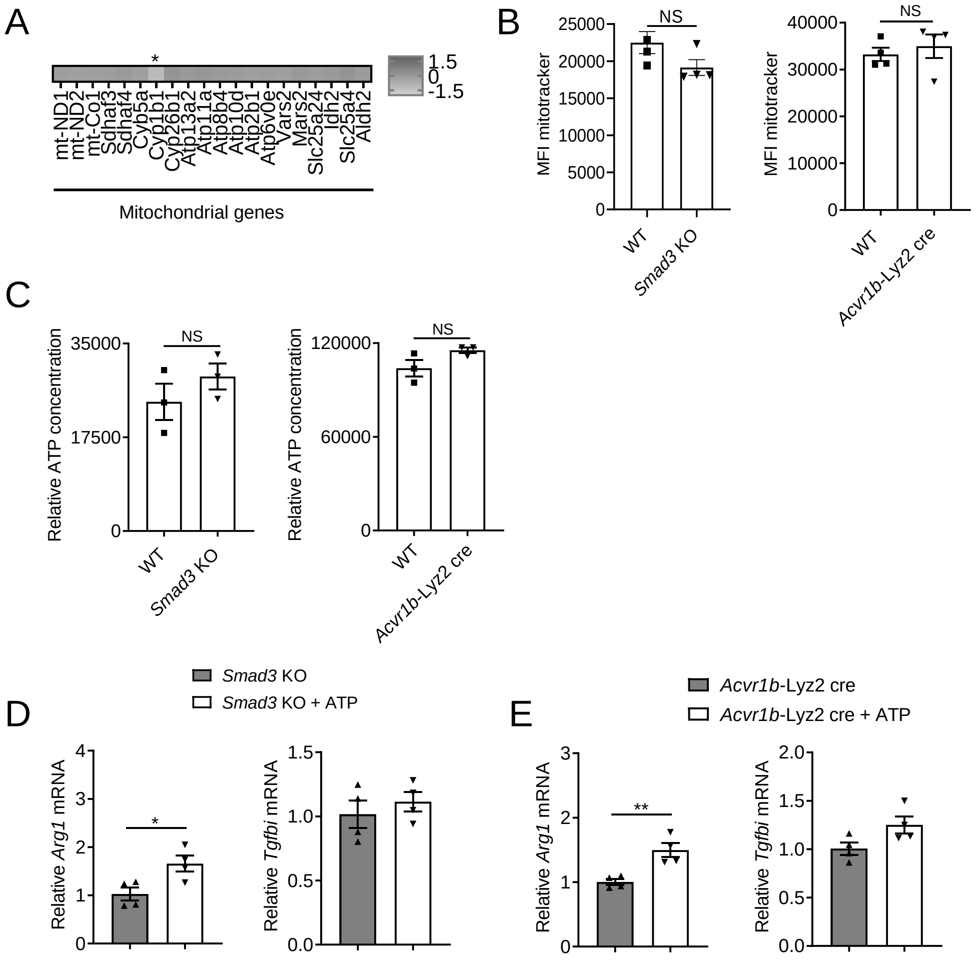

Figure S9 Smad3 controls inflammation and survival during sepsis

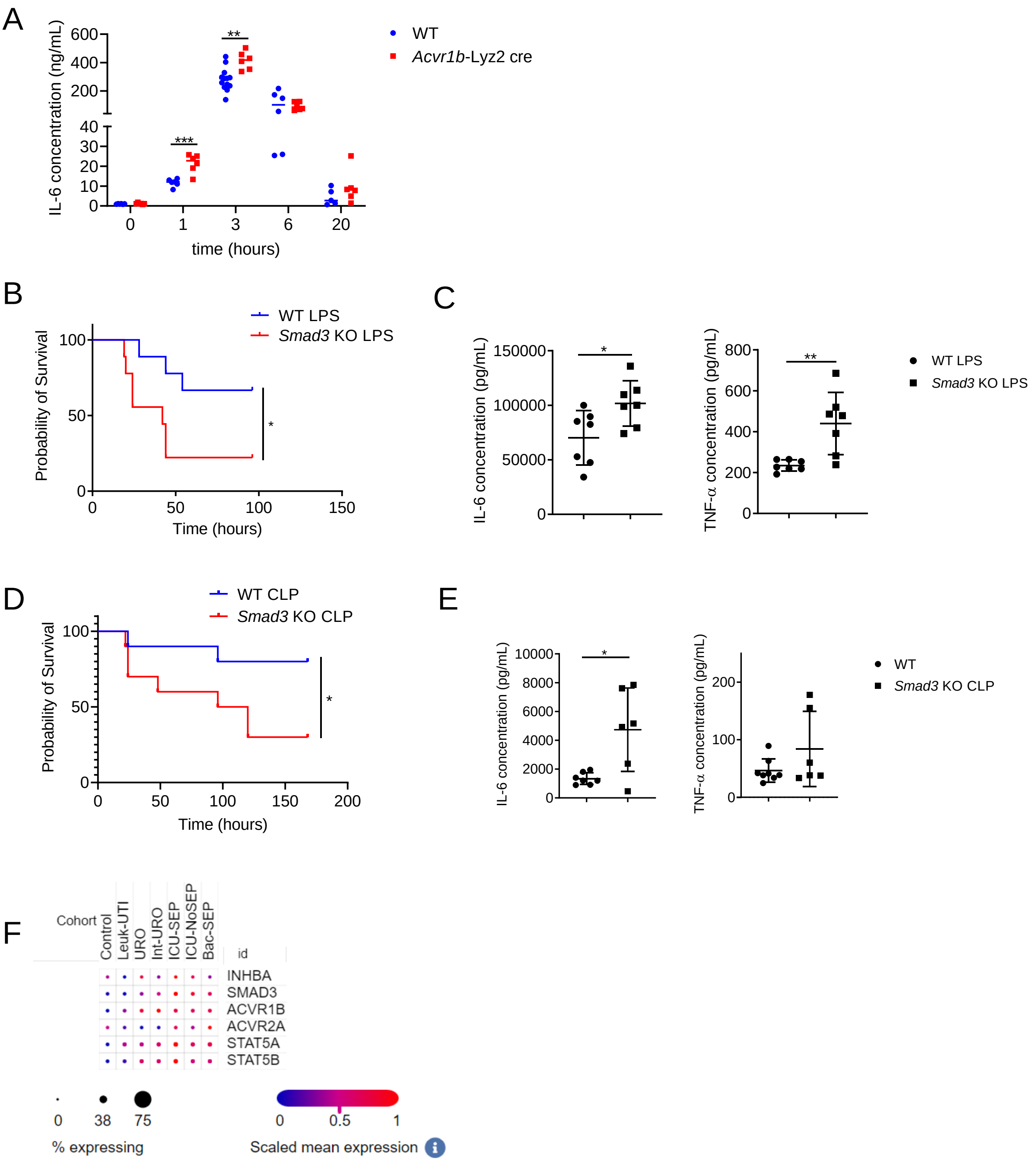

Figure S10 The Activin-Smad3 axis is a crucial modulator of SARS-CoV2 mediated inflammation

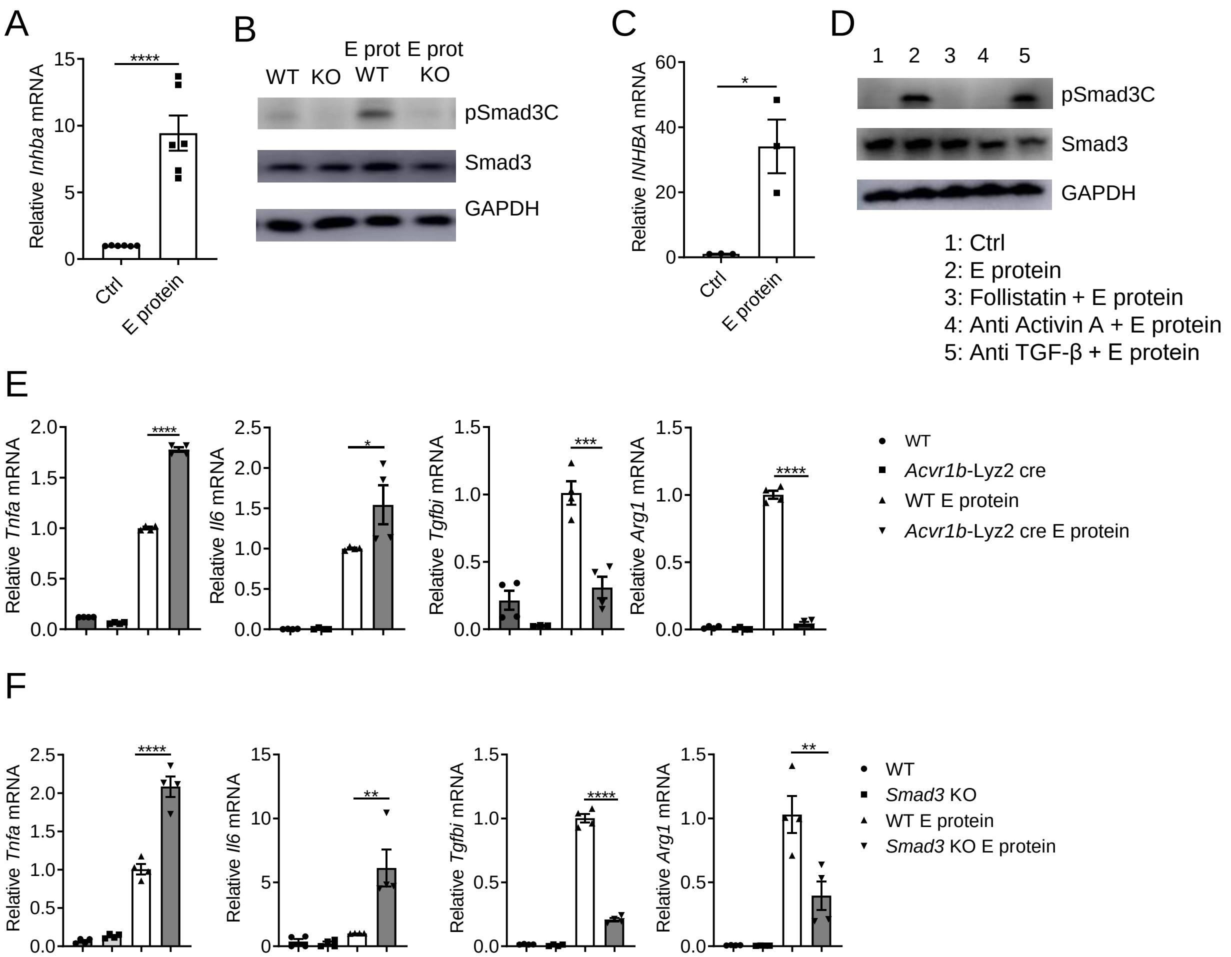

Figure S11 The Activin A-Smad3 axis regulates IMQ-induced inflammation

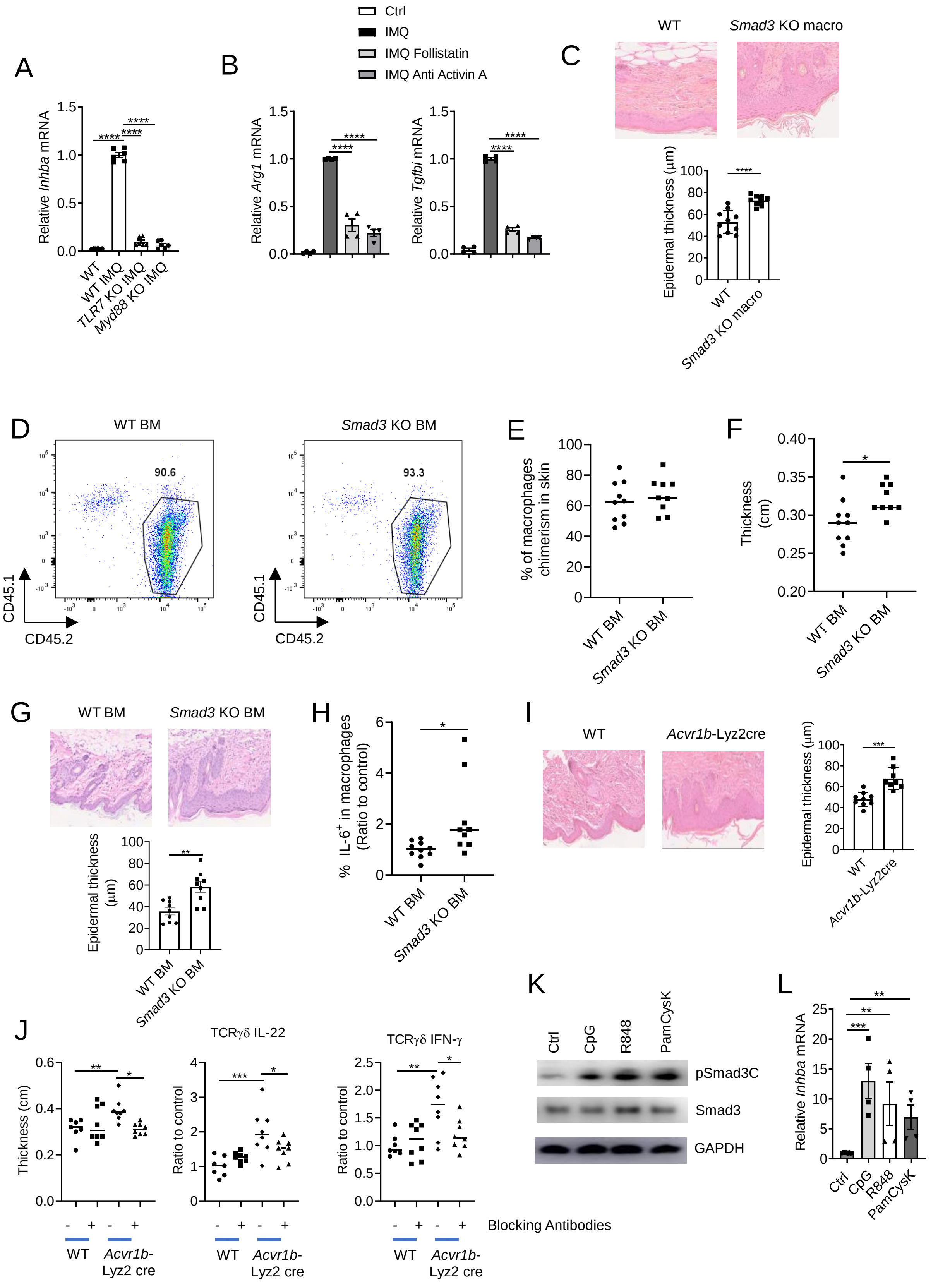

Figure S12 Gating strategies and representative plots for psoriasis experiments

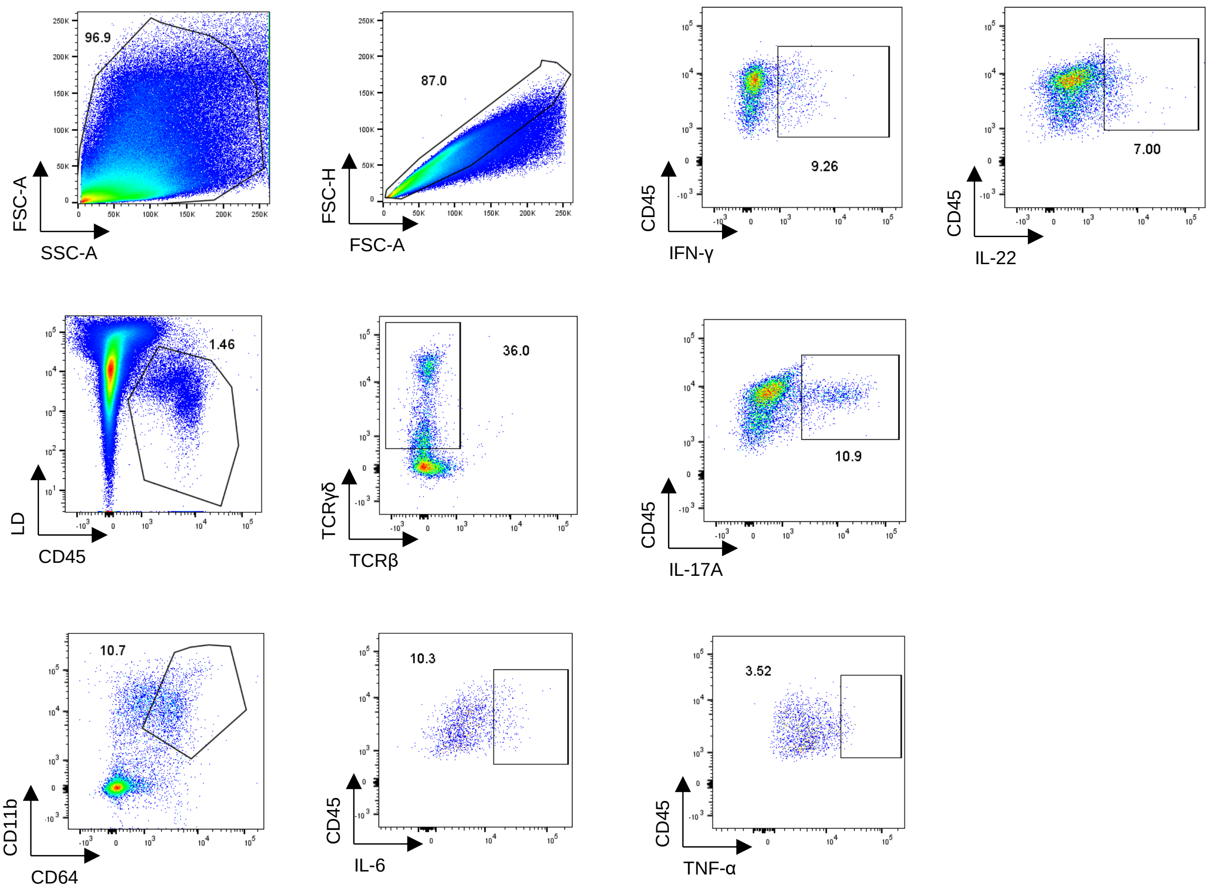

Figure S13 Western blotting quantification of main figures

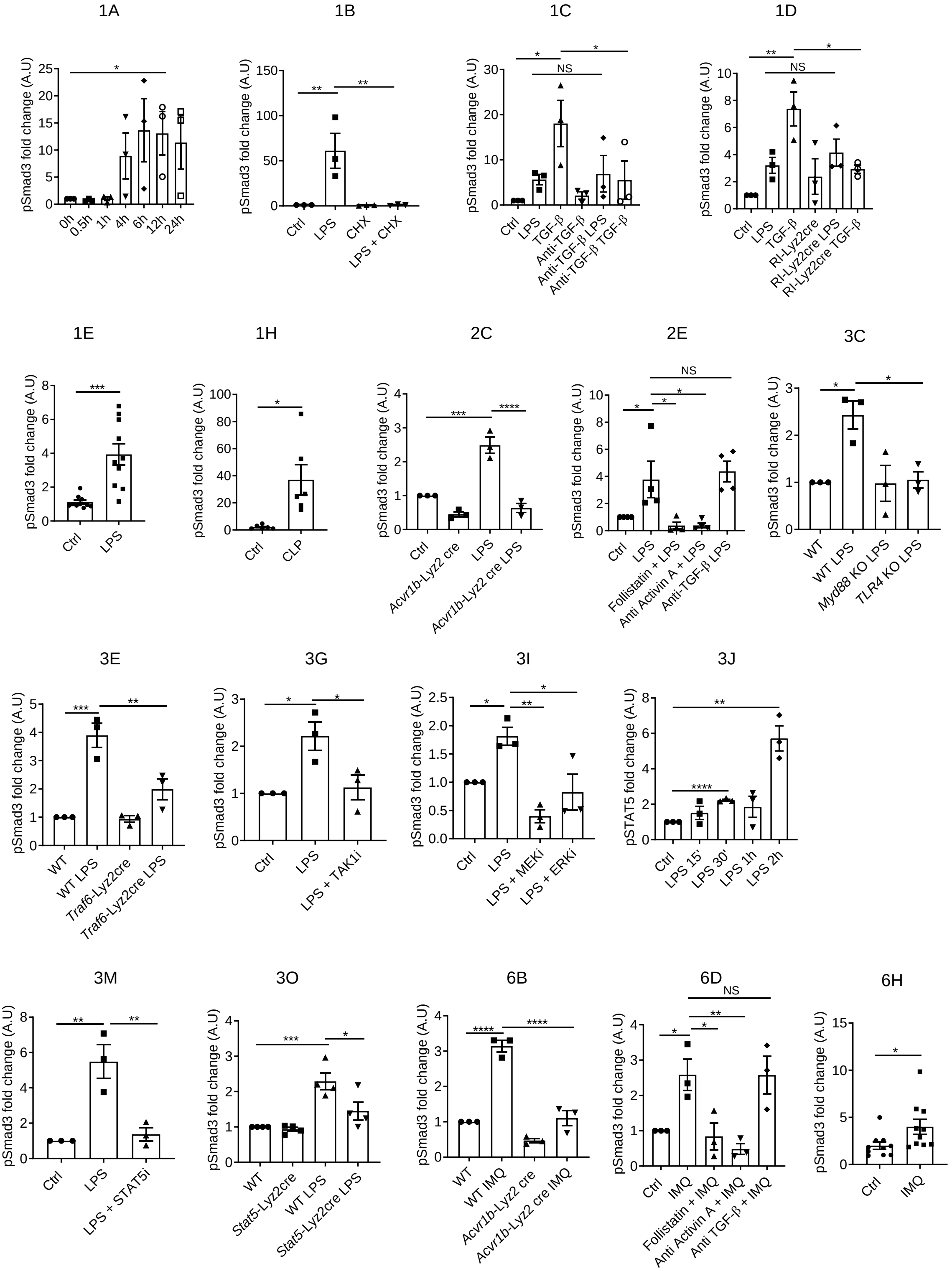

Figure S14 Western blotting quantification of supplemental figures

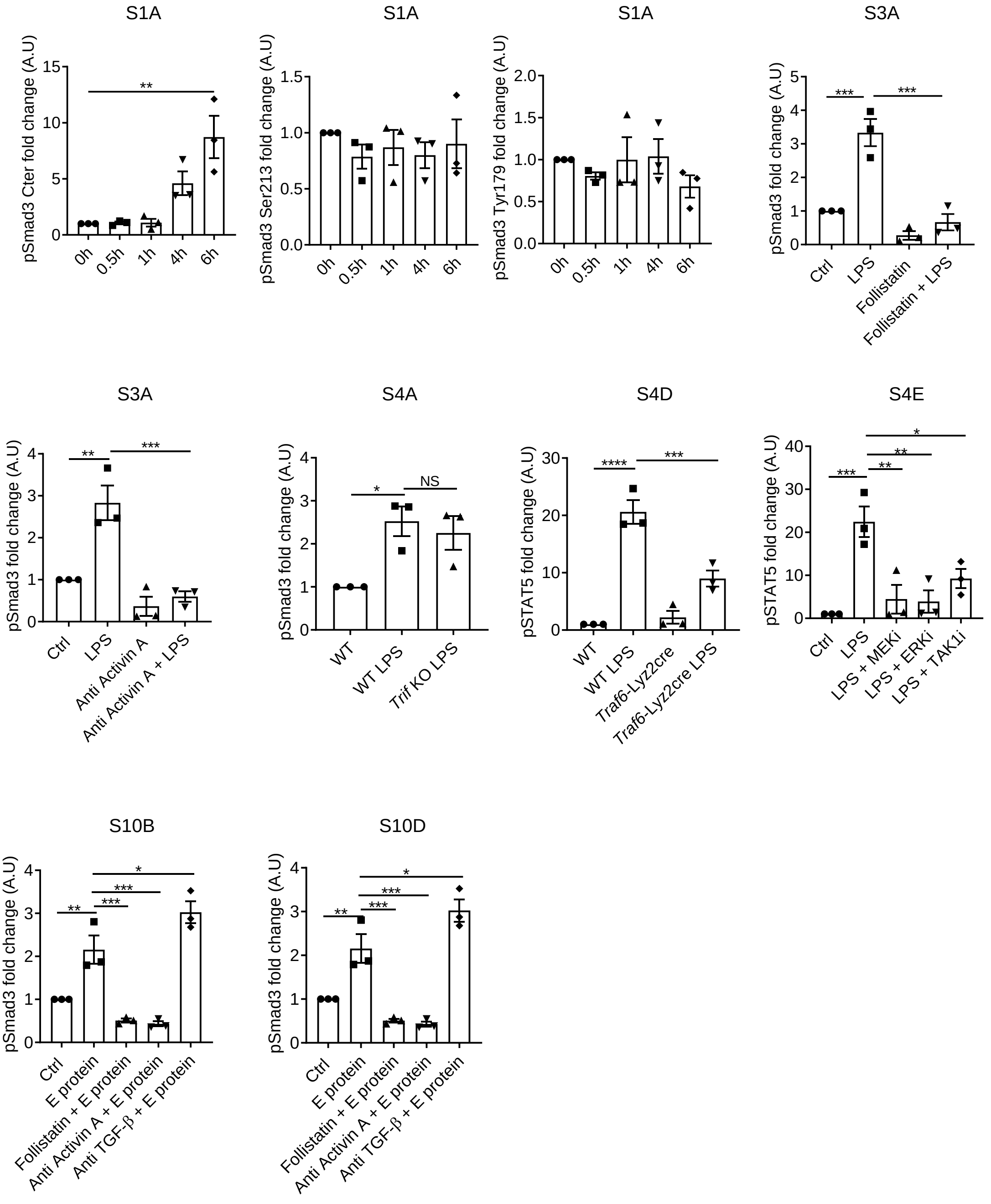

Figure S15 Activin A-induced SMAD3 activation restrains macrophage-mediated inflammation

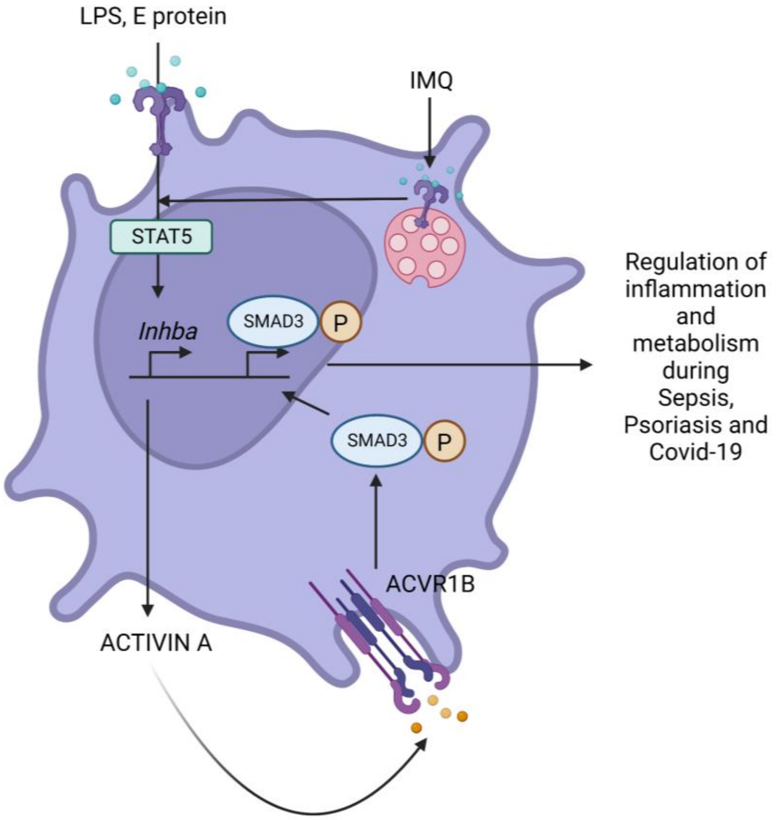

Figure S1. LPS activates Smad3 in a TGF-β independent manner (**A**) Abundance of the indicated proteins in macrophages treated with LPS for the indicated time points. (**B**) RT-qPCR analysis of *Tgfb1* mRNA in macrophages stimulated by LPS for 2, 4 or 6 h. (n=6) (**C**) Activation of a TGF-β reporter cell line during 24 h in presence of TGF-β and/or anti-TGF-β antibody or its isotype control. (n=3) (**D**) RT-qPCR analysis (left panel) or Western blotting (right panel) of the expression of *Tgfbr1* in RI-Lyz2 cre or WT macrophages. (n=4) (**E**) Representative FACS plots for the gating strategy of figure 1F (Sepsis experiment to define pSmad2/3 levels). Cells were first gated on FSC-A and SSC-A followed by exclusion of doublets (FSC-H and FSC-A) and selection of CD45 positive cells. Macrophages were selected based on the expression of F4/80 and lack of Ly6G and neutrophils were F4/80^{-} Ly6G^{+}. The remaining cells (F4/80^{-}Ly6G^{-}) were used to identify DCs (CD11c^{+}CD3^{-}) or B cells (CD19^{+}). Finally, T cells were gated as CD19^{-} and CD3^{+}. (**F**) Bacterial load in the peritoneum of CLP-induced sepsis mice 6 hours after surgery. (n=6) Representative or pooled of at least 2 independent experiments. *P<0.05, by student's T test.

Figure S2. Gene expression of TGF-β superfamily members (**A**) RT-qPCR analysis of the indicated genes in macrophages stimulated by LPS for 2, 4 or 6 h. (n=4-6) Pooled from at least 2 independent experiments. *P<0.05, **P<0.01, by student's T test.

Figure S3. LPS phosphorylates Smad3 in an Activin A dependent manner (**A**) Abundance of the indicated proteins in macrophages pre-treated with Follistatin or an anti-Activin A blocking antibody for 1 h followed by LPS stimulation for 6 h. (**B**) Abundance of the indicated proteins in macrophages treated with Activin A (10 ng/mL) in presence of anti-Activin A antibody or its isotype control for 1 h. (**C**) RT-qPCR showing the recombination

between *Acvr1b* and Lyz2 cre in WT (*Acvr1b*$^{+/+}$ Lyz2 cre+) and *Acvr1b*$^{fl/fl}$ Lyz2 cre+ macrophages which depicts the efficient deletion of *Acvr1b*. (**D**) Abundance of the indicated proteins in macrophages treated with Activin A for 1 h in macrophages isolated from WT or *Acvr1b*-Lyz2 cre mice. (**E**) RT-qPCR analysis of the indicated genes in macrophages pre-treated with Follistatin or an anti-Activin A blocking antibody for 1 h followed by LPS stimulation or not for 24 h. (n=4-6) (**F**) RT-qPCR analysis of the indicated genes in macrophages from WT or *Smad3* KO mice stimulated or not by LPS for 24 h. (n=4-6) (**G**) RT-qPCR analysis of the indicated genes in macrophages from WT or *Acvr1b*-Lyz2cre mice stimulated or not by LPS for 24 h. (n=4-6) (**H**) TNF-α and IL-6 protein levels in macrophages pre-treated with Follistatin or an anti-Activin A blocking antibody for 1 h followed by LPS stimulation or not for 24 h. (**I**) RT-qPCR analysis of *Il6* in macrophages pre-treated or not with a Smad3 inhibitor for 1 h followed by LPS stimulation for 24 h in presence or absence of Activin A. (n=4) Representative or pooled of at least 2 independent experiments. *P<0.05, **P<0.01, ***P<0.005, ****P<0.001 by one-way ANOVA.

Figure S4. LPS induces Activin A expression through a TLR4-MyD88-MAPK-STAT5 pathway (**A**) Abundance of the indicated proteins in macrophages isolated from WT or *Trif* KO mice and treated with LPS for 6 h. (**B**) Predicted binding sites for STAT5 in the *Inhba* gene promoter. (**C**) RT-qPCR analysis of *Stat5a* and *Stat5b* in macrophages stimulated by LPS for the indicated times. (n=4) (**D**) Abundance of the indicated proteins in macrophages isolated from WT or *Traf6*-Lyz2 cre mice and stimulated by LPS for 2 h. (**E**) Abundance of the indicated proteins in macrophages pre-treated with a MEK, ERK or TAK1 inhibitor for 1 h

followed by LPS stimulation for 6 h. Representative or pooled of at least 2 independent experiments. *P<0.05, ** P<0.01 by one-way ANOVA.

Figure S5. Validation of knock-out macrophages and inhibitors Abundance of the indicated proteins in macrophages from WT or *MyD88* KO (**A**), *Traf6*-Lyz2 cre (**B**), *Stat5*-Lyz2 cre (**C**) or treated with LPS for 1h (**D**), 30 min (**E**) or 2h (**F**) and pre-treated with their respective inhibitors for 1h.

Figure S6. RNAseq of Smad3 deficient macrophages (**A**) Volcano plot depicting up-regulated (right) and down-regulated (left) genes obtained from RNA-seq analysis of WT or *Smad3* KO macrophages stimulated with LPS for 24 h. (**B**) Up-regulated (upper panel) and down-regulated (lower panel) pathways in WT or *Smad3* KO macrophages stimulated with LPS for 24 h. Data are from 4 biological replicates.

Figure S7. The Activin A-Smad3 pathway regulates macrophage ATP metabolism during inflammation Mitotracker staining in macrophages stimulated with LPS for 24 h and isolated from WT, *Smad3* KO (**A**) or *Acvr1b*-Lyz2 cre mice (**B**) (n=60). Mitochondrial potential membrane (ϕm, left panel) and mitochondrial ROS staining (right panel) in macrophages stimulated with LPS for 24 h and isolated from *Smad3* KO mice (**C**) or WT mice pre-treated with Anti-Activin A antibody or Follistatin for 1 h (**D**). (**E**) Mitotracker staining in macrophages pre-treated with Anti-Activin A antibody or Follistatin for 1 h and stimulated with LPS for 24 h. (n=9) (**F**) RT-qPCR analysis of *Arg1* and *Tgfbi* expression in macrophages pre-treated with Follistatin for 1 h and stimulated with LPS for 24 h in combination with ATP. (n=4) (**G**) RT-qPCR analysis of *Entpd1* expression (encoding CD39)

in macrophages stimulated or not with LPS for 24 h and isolated from *Acvr1b*-Lyz2 cre mice. (n=4) (**H**) RT-qPCR analysis of *Arg1* and *Tgfbi* expression in macrophages pre-treated with Follistatin for 1 h and stimulated with LPS for 24 h in combination (or not) with ATP and a CD73 inhibitor or a CREB inhibitor. (n=4-6) (**I**) RT-qPCR analysis of *Nt5e* expression (encoding CD73) in macrophages stimulated with LPS and Activin A for 24 h and isolated from *Smad3* KO or WT mice. (n=4) (**J**) Graphic summary representing the effect of the Activin A-Smad3 pathway in reprogramming ATP metabolism. Representative or pooled of at least 2 independent experiments. *P<0.05, **P<0.01, ***P<0.005, ****P<0.001 by student's T test (A, B and F) or one-way ANOVA (E, G-I).

Figure S8. The Activin A-Smad3 pathway regulates macrophage ATP metabolism during inflammation (2) (**A**) Heatmap representing significantly down-regulated genes in macrophages from *Smad3* KO mice (compared to WT macrophages). (**B**) Mitotracker staining in macrophages isolated from *Smad3* KO mice (left panel) or *Acvr1b*-Lyz2 cre mice (right panel). (n=4) (**C**) ATP production (intracellular) in macrophages isolated from *Smad3* KO mice (left panel) or *Acvr1b*-Lyz2 cre mice (right panel). RT-qPCR analysis of *Arg1* and *Tgfbi* expression in macrophages stimulated for 24 h with or without 20 µM of ATP and isolated from *Smad3* KO mice (**D**) or *Acvr1b*-Lyz2cre mice (**E**). (n=4). Representative or pooled of at least 2 independent experiments. *P<0.05, **P<0.01 by student's T test (B-E).

Figure S9. Smad3 controls inflammation and survival during sepsis (**A**) IL-6 levels in serum of WT or *Acvr1b*-Lyz 2cre mice injected intraperitoneally or not with LPS for the indicated times. Zero and three hours time points are taken from Figure 5B. (**B**) (n=5-13). Survival of WT or *Smad3* KO mice injected intraperitoneally with LPS. (n=9) (**C**) TNF-α and

IL-6 levels in serum of WT or *Smad3* KO mice injected intraperitoneally with LPS for 3 h. (n=7) (**D**) Survival of WT or *Smad3* KO mice subjected to CLP-surgery. (n=10) (**E**) TNF-α and IL-6 levels in serum of WT or *Smad3* KO mice subjected to CLP-surgery for 18 h. (n=6-8) (**F**) Relative expression levels of the indicated genes in patients grouped by cohort. Data were analyzed using the Single Cell Portal developed by the Broad Institute (29). Pooled from at least 2 independent experiments. *P<0.05 by log-rank (Mantel-Cox test, B and D) and student's T test (A,C and E).

Figure S10. The Activin-Smad3 axis is a crucial modulator of SARS-CoV2 mediated inflammation (**A**) RT-qPCR analysis of *Inhba* expression in macrophages stimulated with E protein for 2 h. (n=6) (**B**) Abundance of the indicated proteins in macrophages isolated from WT or *Acvr1b*-Lyz2 cre mice (KO) and stimulated with E protein for 6 h. (**C**) RT-qPCR analysis of *INHBA* expression in human macrophages stimulated with E protein for 2 h. (n=3) (**D**) Abundance of the indicated proteins in human macrophages pre-treated with Follistatin, an anti-Activin A or an anti-TGF-β blocking antibody for 1 h followed by E protein stimulation for 6 h. (**E**) RT-qPCR analysis of the indicated genes in macrophages from WT or *Acvr1b*-Lyz2 cre mice stimulated or not with E protein for 24 h. (n=4) (**F**) RT-qPCR analysis of the indicated genes in macrophages from WT or *Smad3* KO mice stimulated or not with E protein for 24 h. (n=4) Representative or pooled of at least 2 independent experiments. *P<0.05, **P<0.01, ***P<0.005, ****P<0.001 by student's T test (A and C) or one-way ANOVA (E and F).

Figure S11. The Activin A-Smad3 axis regulates IMQ-induced inflammation (**A**) RT-qPCR analysis of *Inhba* in macrophages stimulated with IMQ for 2 h and isolated from WT,

TLR7 or *MyD88* KO mice. (n=6) (**B**) RT-qPCR analysis of the indicated genes in macrophages pre-treated with Follistatin or anti-Activin A antibody for 1 h and stimulated with IMQ for 24 h. (n=4) (**C**) Macrophages from WT or *Smad3* KO macrophages were transferred intradermally in the skin of CD45.1 WT mice followed by IMQ topical application for 6 consecutive days. Skin was then harvested and analyzed by H&E staining. (n=9) (**D**) Representative FACS plot of WT and *Smad3* KO BM reconstitution in the blood. (**E**) Macrophages (CD11b+CD64+CD45.2+CD45.1-) reconstitution in the skin. (n=9-10) (**F**) Skin thickness in WT or *Smad3* KO BM mice 6 days after imiquimod application. (n=9-10) (**G**) Representative histology samples and quantification of epidermis thickness in WT and *Smad3* KO BM. (**H**) Production of IL-6 in CD45.2 macrophages from WT and *Smad3* KO BM. (n=9-10) (**I**) H&E staining of the skin from WT or *Acvr1b*-Lyz2cre mice after 6 days of IMQ application. (n=8-9) (**J**) WT or *Acvr1b*-Lyz2 cre mice were injected with anti TNF-α, IL-6, IL-1β and IL23A antibodies and treated with IMQ topical application for 6 consecutive days, then harvested and analyzed. Skin thickness (left panel). TCRγδ cytokines production in the skin (middle and right panels). (n=7-8) (**K**) Abundance of pSmad3C, Smad3, and GAPDH in macrophages treated with the indicated TLR ligands for 6 h. (**L**) RT-qPCR analysis of *Inhba* expression in macrophages stimulated with the indicated TLR ligands for 6 h. (n=4) Representative or pooled of at least 2 independent experiments. *P<0.05, **P<0.01, ***P<0.005 by student's T test (C, F, G-I) or one-way ANOVA (A, B, J and L).

Figure S12. Gating strategies and representative plots for psoriasis experiments

Representative FACS plot for the gating strategy of psoriasis experiments. Cells were first gated on FSC-A and SSC-A followed by exclusion of doublets and gating of $CD45^+$ live cells.

TCRγδ+TCRβ- were further identified as γδ T cells and their production of IFN-γ, IL-17A and IL-22 was assessed. Macrophages were gated as $CD11b^+$ $CD64^+$ and their production of IL-6 and TNF-α was determined.

Fig S13. Western blotting quantification of main figures

Fig S14. Western blotting quantification of supplemental figures

Figure S15. Activin A-induced SMAD3 activation restrains macrophage-mediated inflammation
