# Supplementary material for: Activin A activation of Smad3 mitigates innate inflammation in mouse models of psoriasis and sepsis
Source: J Clin Invest. 2025 Mar 11;135(9):e187063. doi: 10.1172/JCI187063 (PMC12043092; doi:10.1172/JCI187063)
Supplement: Unedited blot and gel images [file jci-135-187063-s255.pdf]

Fig 1A

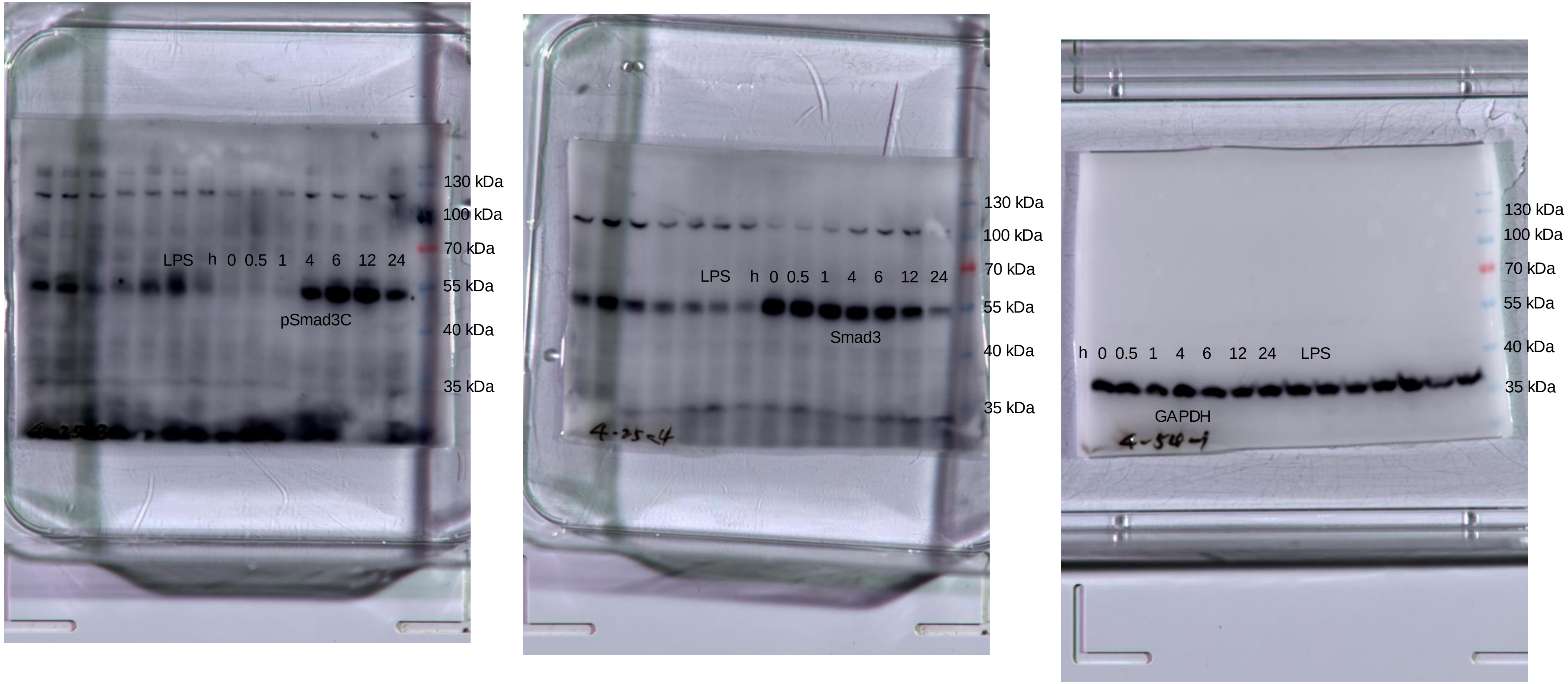
130 kDa
100 kDa
70 kDa
55 kDa
40 kDa
35 kDa
LPS h 0 0.5 1 4 6 12 24
pSmad3C
130 kDa
100 kDa
70 kDa
55 kDa
40 kDa
35 kDa
LPS h 0 0.5 1 4 6 12 24
Smad3
130 kDa
100 kDa
70 kDa
55 kDa
40 kDa
35 kDa
h 0 0.5 1 4 6 12 24 LPS
GAPDH

Fig 1B

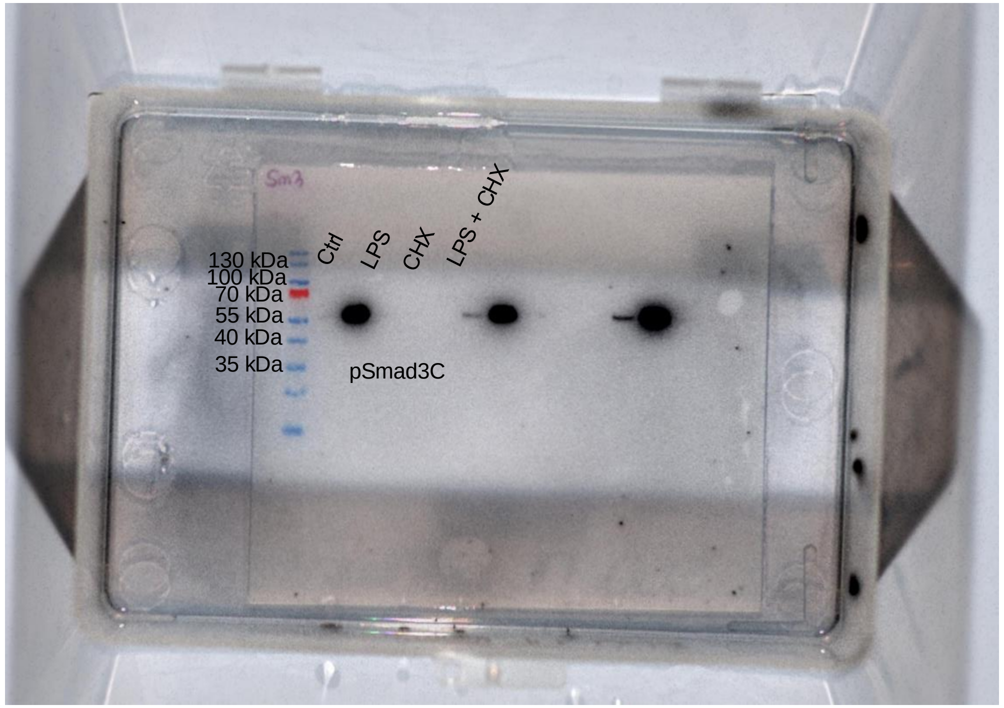
Sm3
Ctrl
LPS
CHX
LPS + CHX
130 kDa
100 kDa
70 kDa
55 kDa
40 kDa
35 kDa
pSmad3C

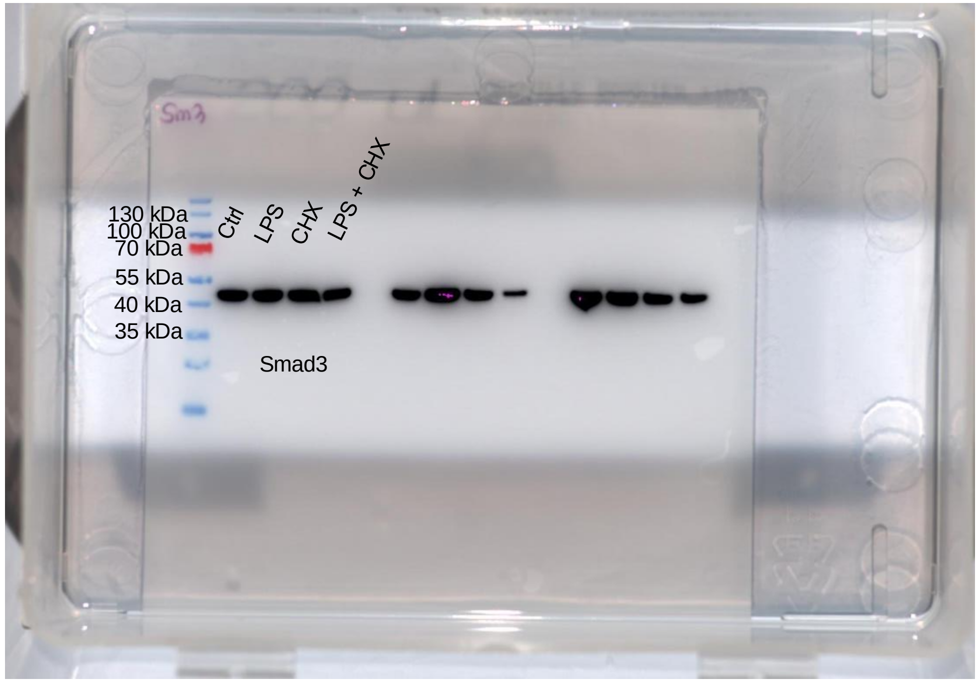
Sm3
Ctrl
LPS
CHX
LPS + CHX
130 kDa
100 kDa
70 kDa
55 kDa
40 kDa
35 kDa
Smad3

Fig 1C

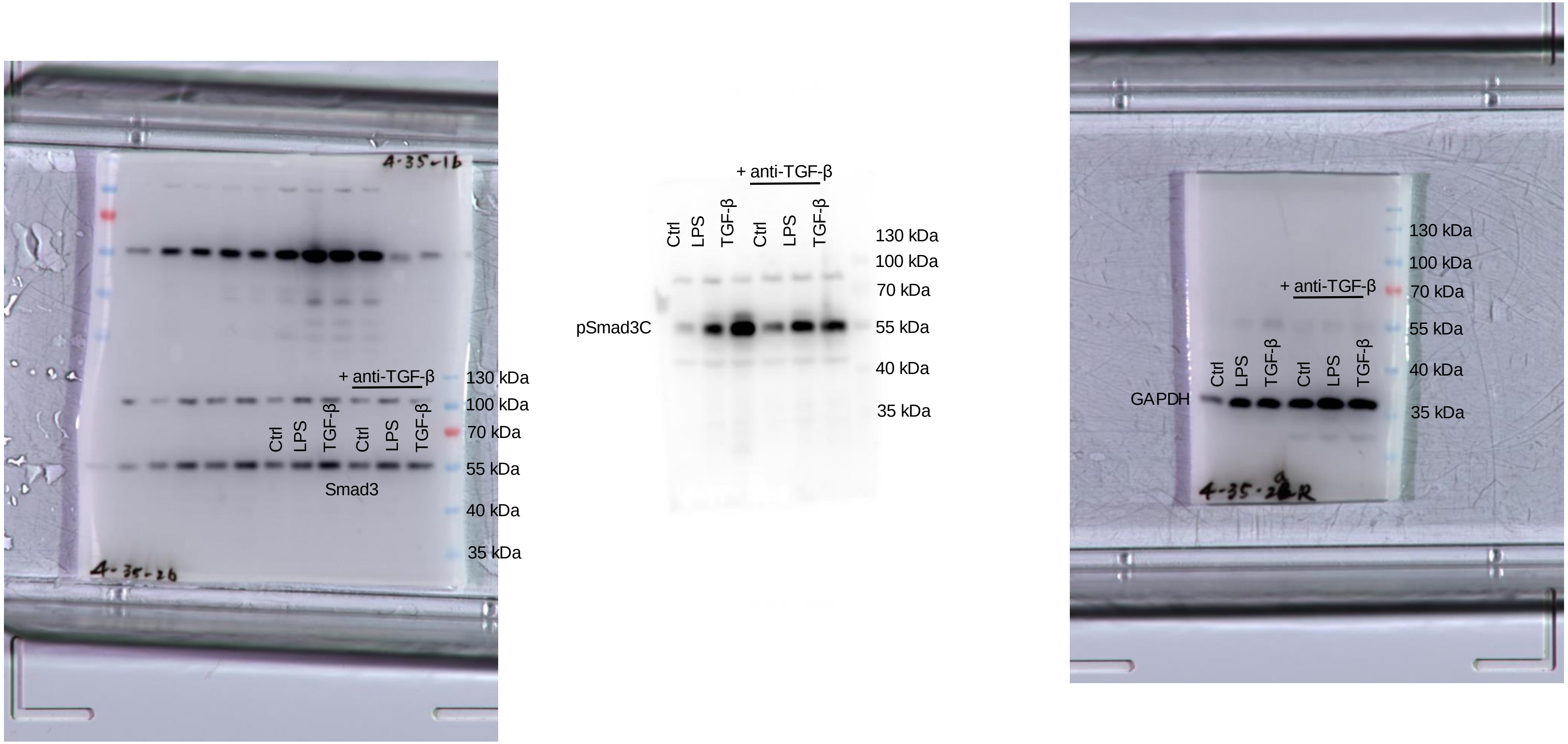
+ anti-TGF-β
Ctrl
LPS
TGF-β
Ctrl
LPS
TGF-β
130 kDa
100 kDa
70 kDa
55 kDa
40 kDa
35 kDa
Smad3
pSmad3C
GAPDH

Fig 1D

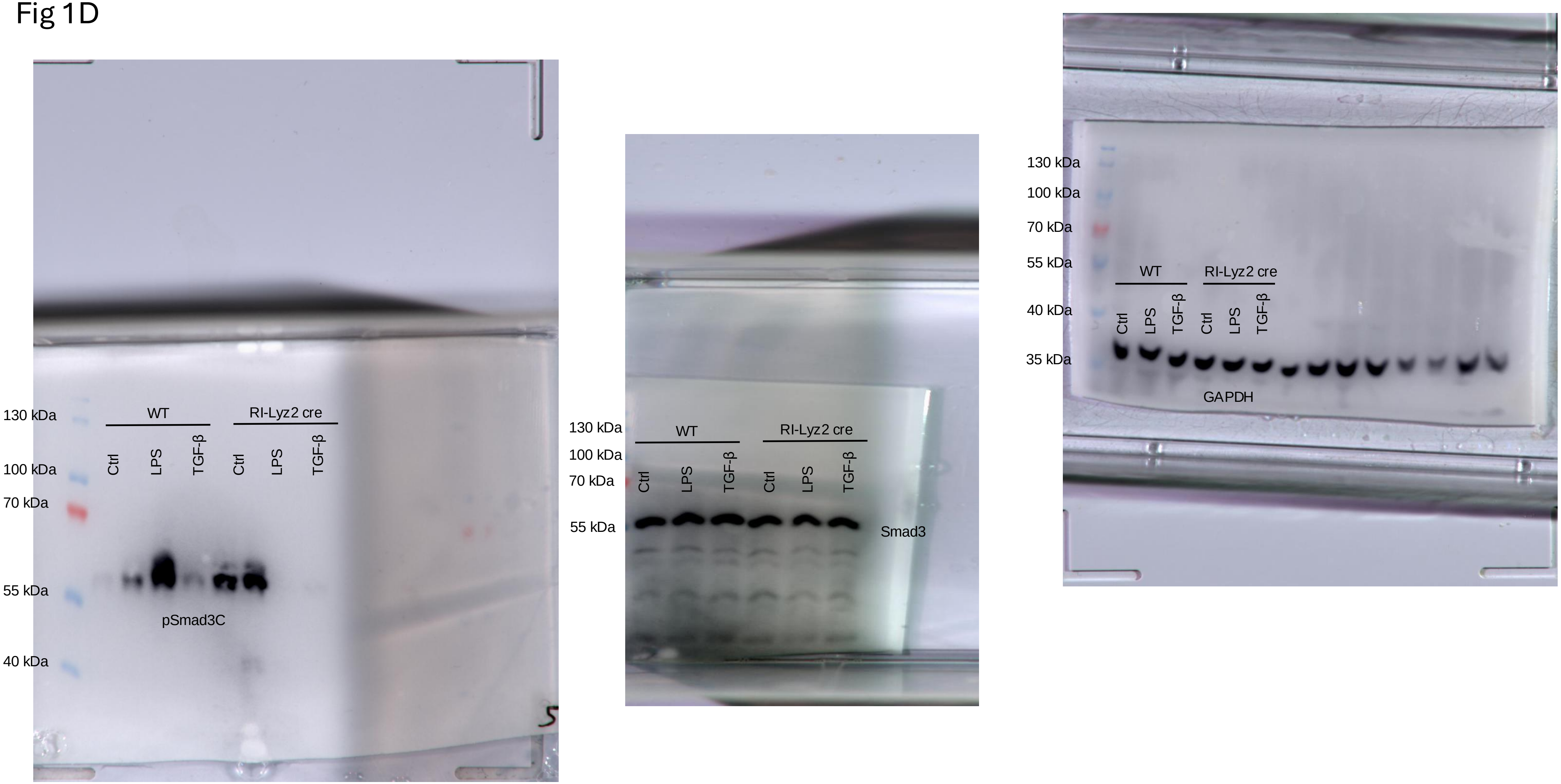
130 kDa
100 kDa
70 kDa
55 kDa
40 kDa
WT
RI-Lyz2 cre
Ctrl
LPS
TGF-β
Ctrl
LPS
TGF-β
pSmad3C
130 kDa
100 kDa
70 kDa
55 kDa
WT
RI-Lyz2 cre
Ctrl
LPS
TGF-β
Ctrl
LPS
TGF-β
Smad3
130 kDa
100 kDa
70 kDa
55 kDa
40 kDa
35 kDa
WT
RI-Lyz2 cre
Ctrl
LPS
TGF-β
Ctrl
LPS
TGF-β
GAPDH

Fig 1E

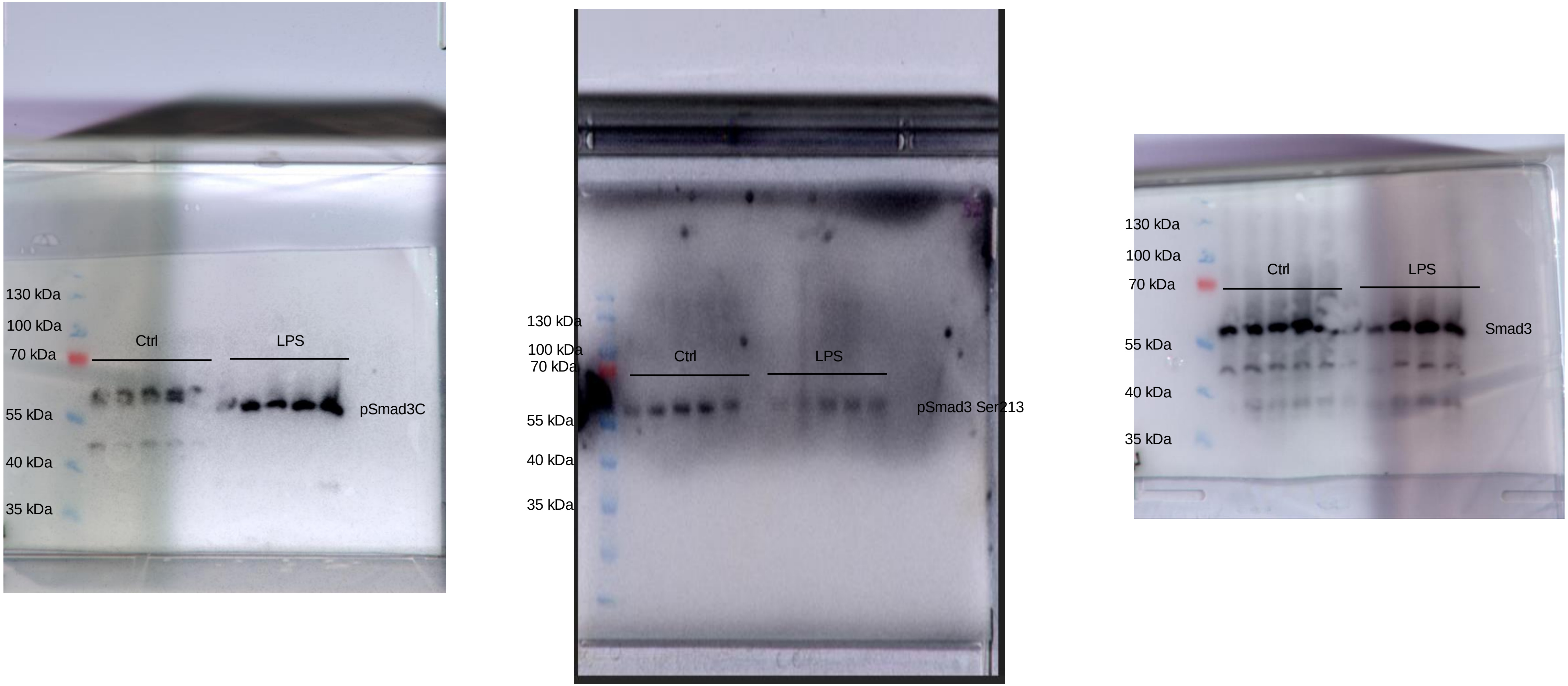
130 kDa
100 kDa
70 kDa
55 kDa
40 kDa
35 kDa
Ctrl
LPS
pSmad3C
130 kDa
100 kDa
70 kDa
55 kDa
40 kDa
35 kDa
Ctrl
LPS
pSmad3 Ser213
130 kDa
100 kDa
70 kDa
55 kDa
40 kDa
35 kDa
Ctrl
LPS
Smad3

Fig 1H

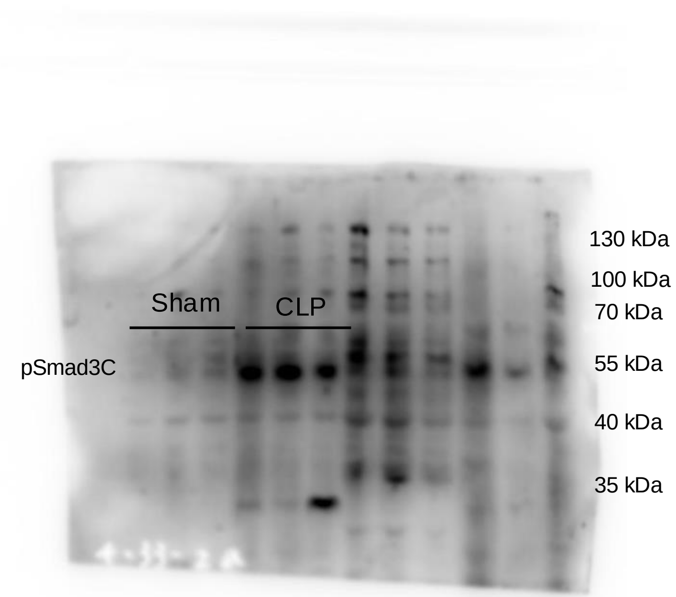

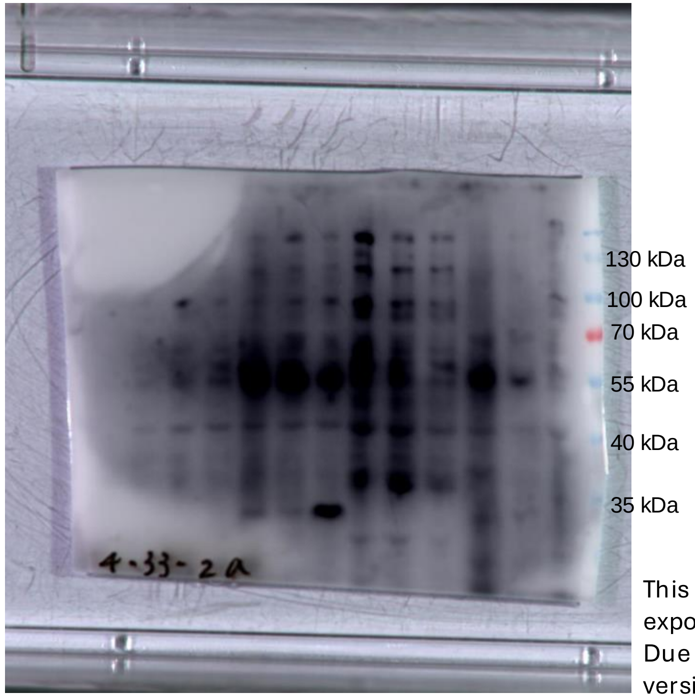

This is the same gel than above but re-exposed in order to see the marker.
Due to overexposure we used the above version with less exposure for publication

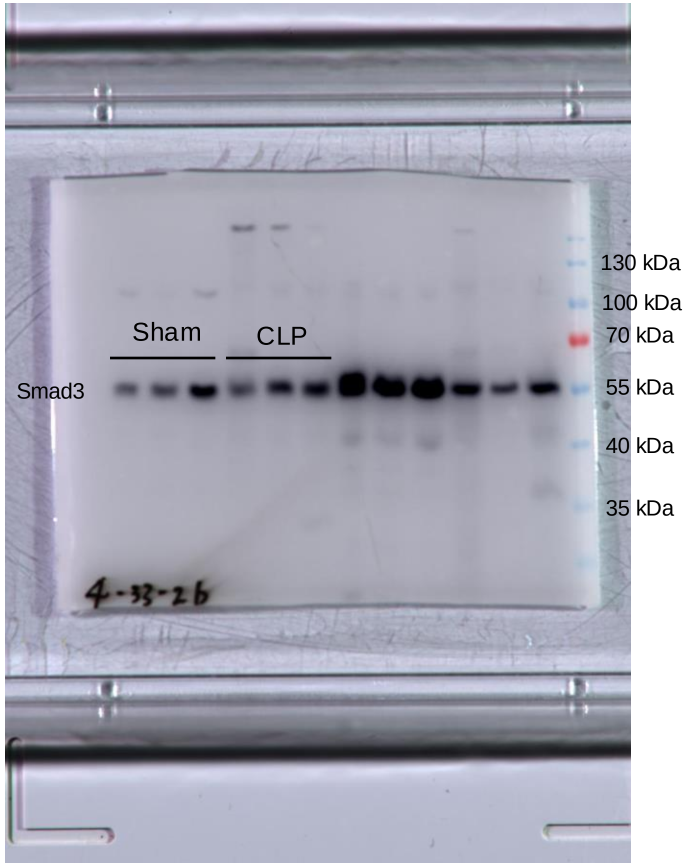

Fig S1A

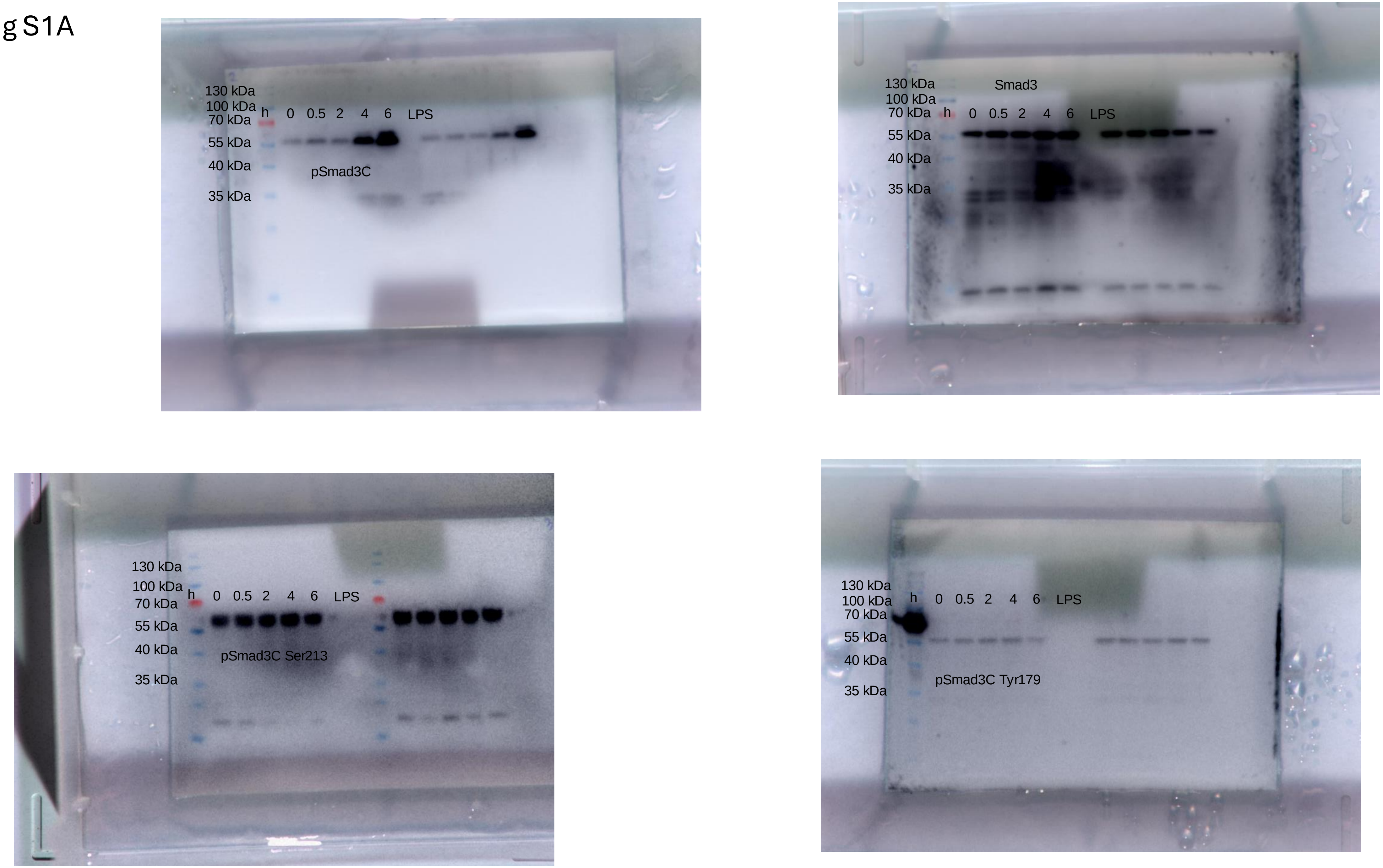
130 kDa
100 kDa
70 kDa
55 kDa
40 kDa
35 kDa
h
0
0.5
2
4
6
LPS
pSmad3C
130 kDa
100 kDa
70 kDa
55 kDa
40 kDa
35 kDa
Smad3
h
0
0.5
2
4
6
LPS
130 kDa
100 kDa
70 kDa
55 kDa
40 kDa
35 kDa
h
0
0.5
2
4
6
LPS
pSmad3C Ser213
130 kDa
100 kDa
70 kDa
55 kDa
40 kDa
35 kDa
h
0
0.5
2
4
6
LPS
pSmad3C Tyr179

Fig S1D

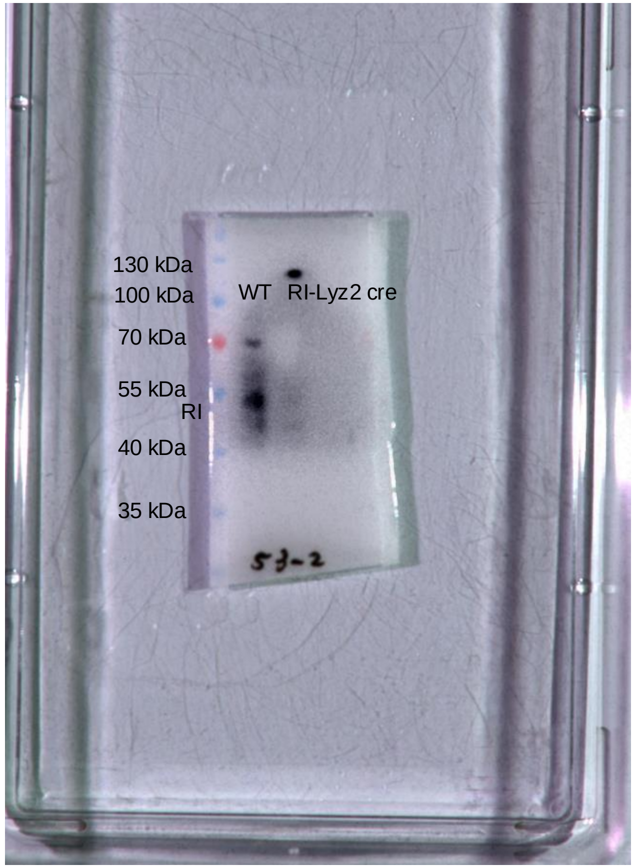
130 kDa
100 kDa
WT
RI-Lyz2 cre
70 kDa
55 kDa
RI
40 kDa
35 kDa

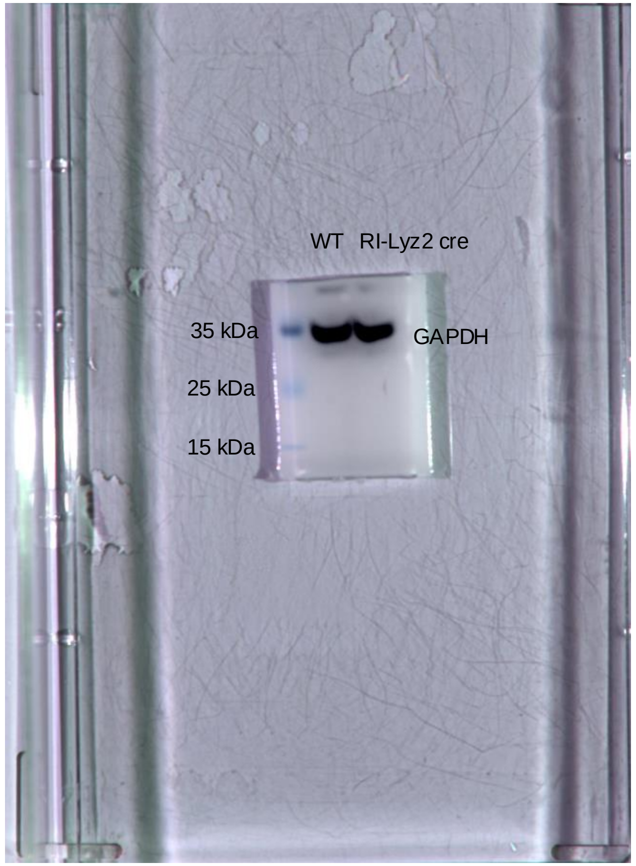
WT
RI-Lyz2 cre
35 kDa
GAPDH
25 kDa
15 kDa

Fig 2C

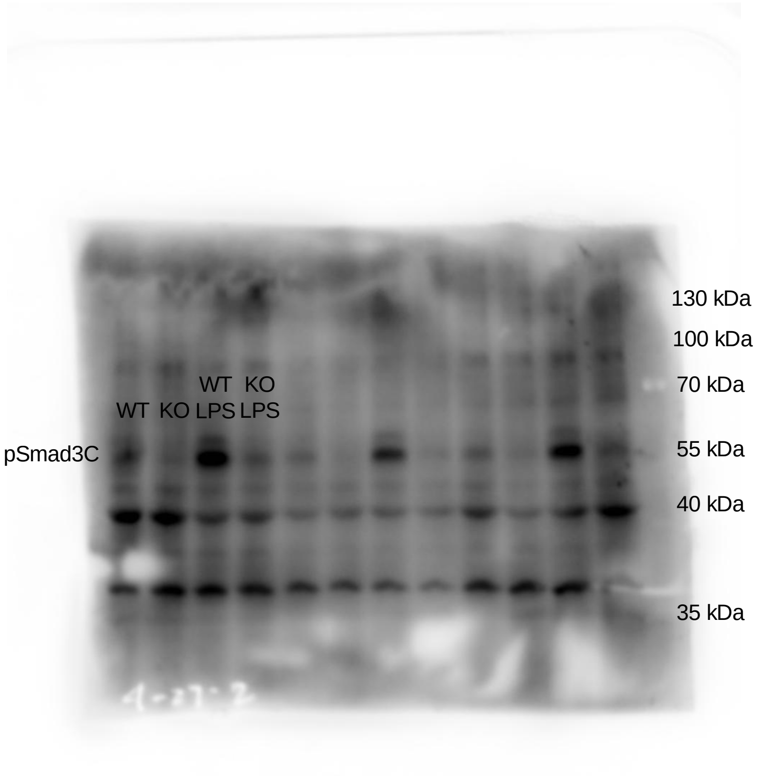

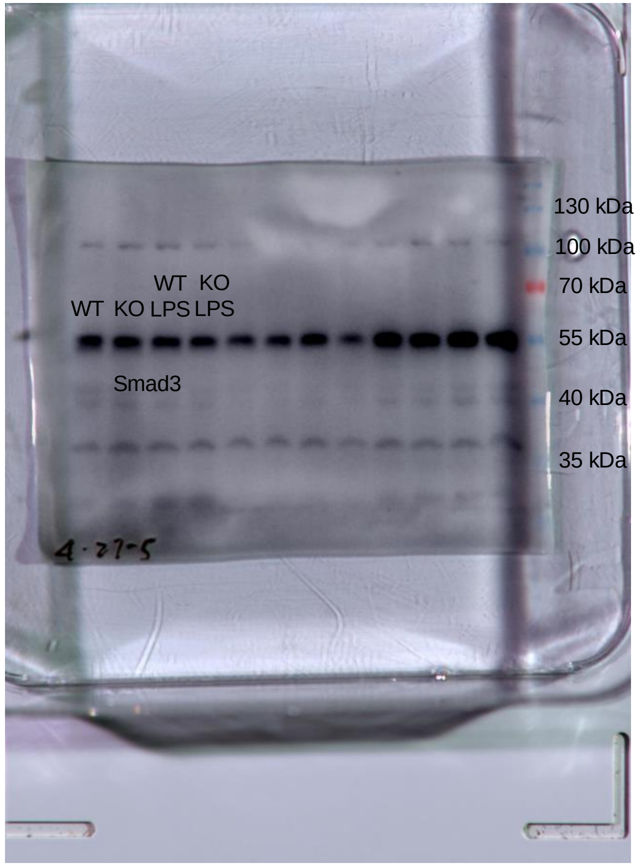

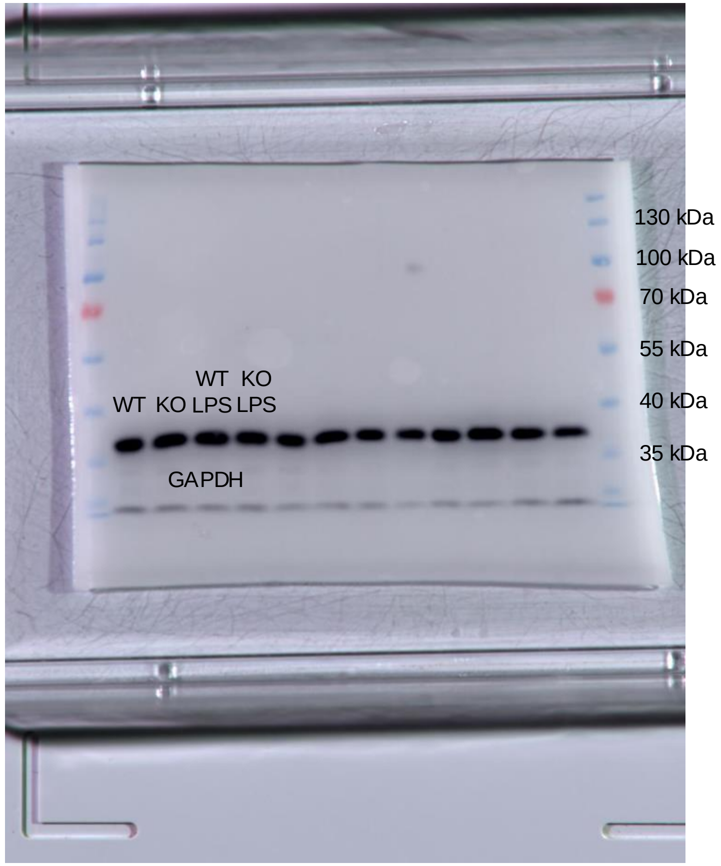

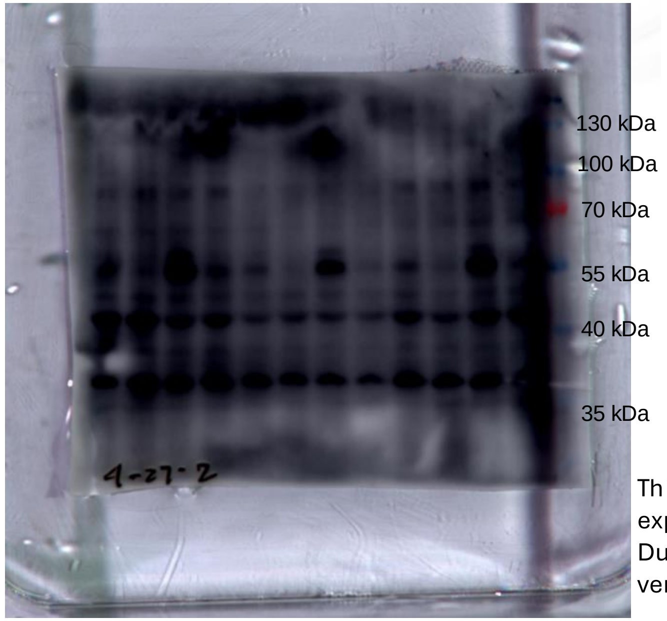

This is the same gel than above but re-exposed in order to see the marker.
Due to overexposure we used the above version with less exposure for publication

Fig 2E

1: Ctrl
2: LPS
3: Follistatin + LPS
4: Anti Activin A + LPS
5: Anti-TGF-β + LPS

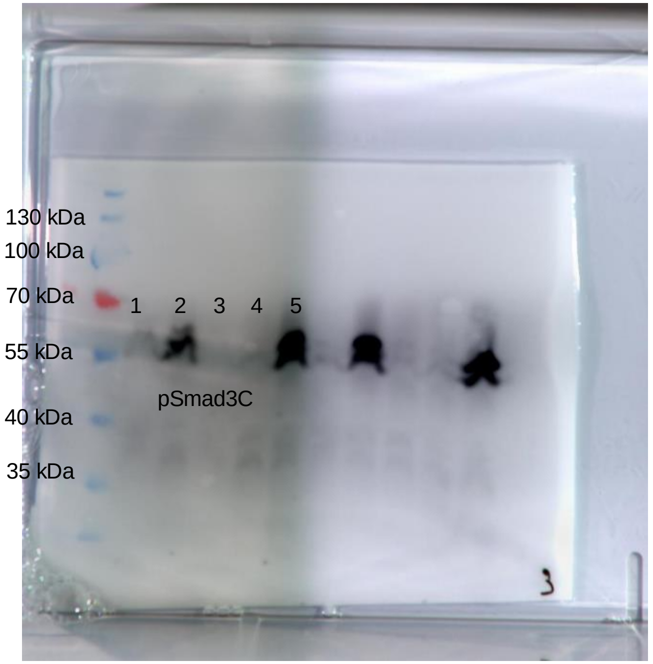

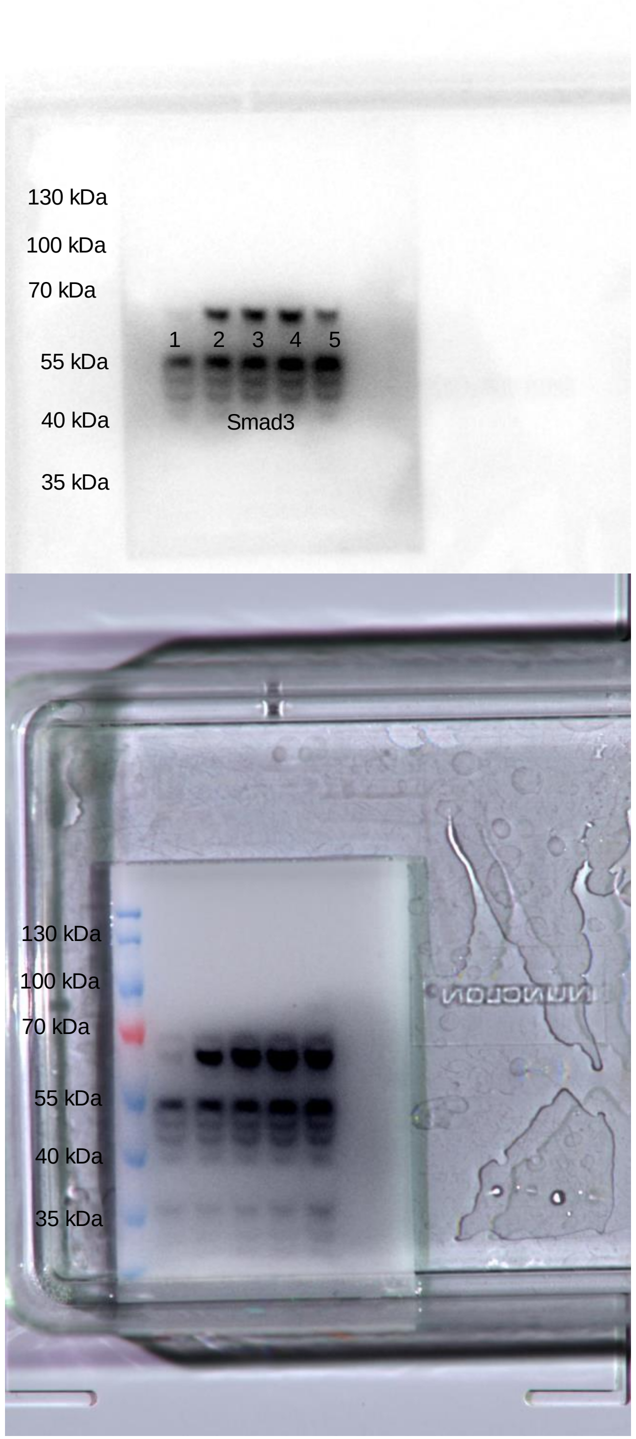

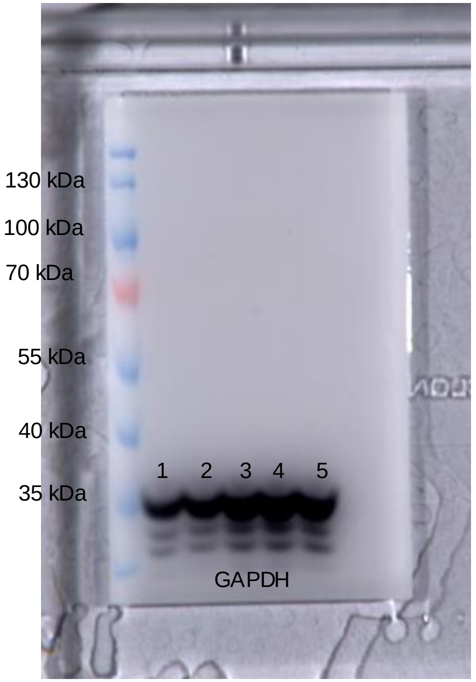

This is the same gel than above but re-exposed in order to see the marker.
Due to overexposure we used the above version with less exposure for publication

Fig S3A

1: Ctrl
2: LPS
3: Follistatin
4: Follistatin LPS
5: Ctrl
6: LPS
7: Anti Activin A
8: Anti Activin A LPS

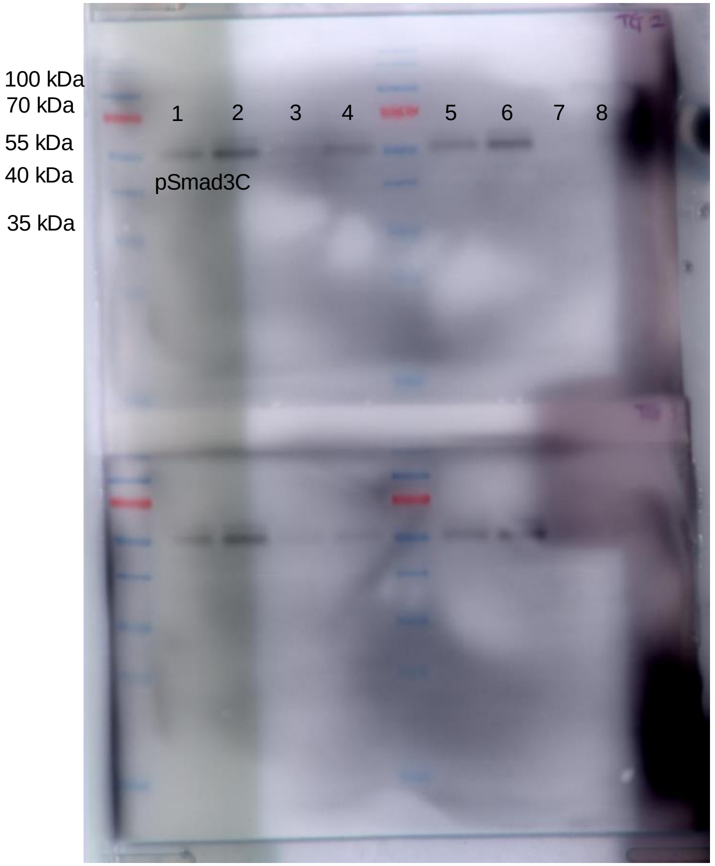

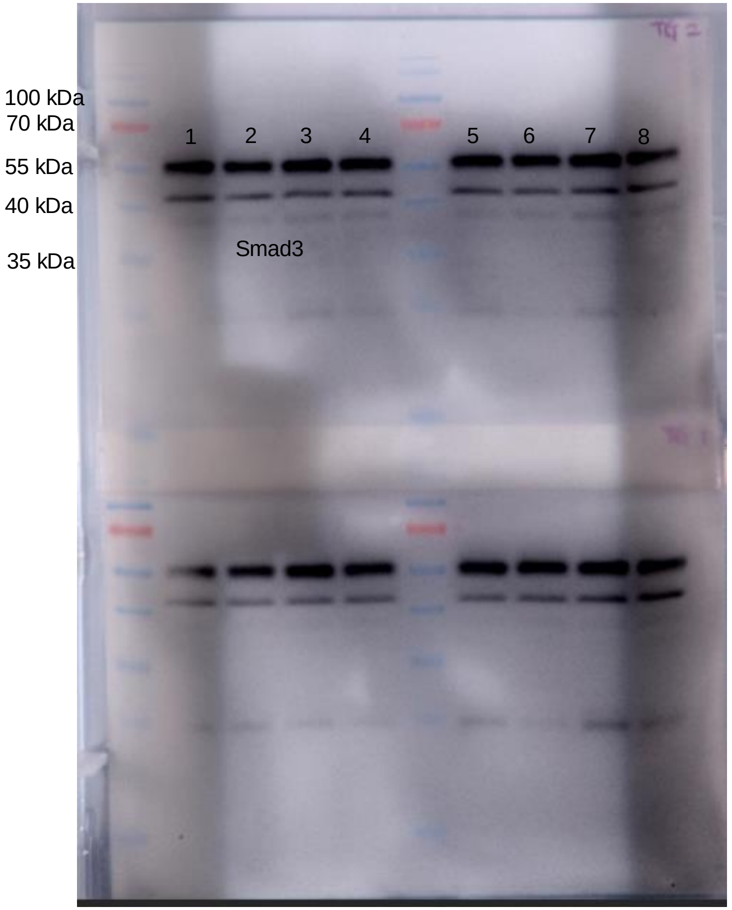

Fig S3B

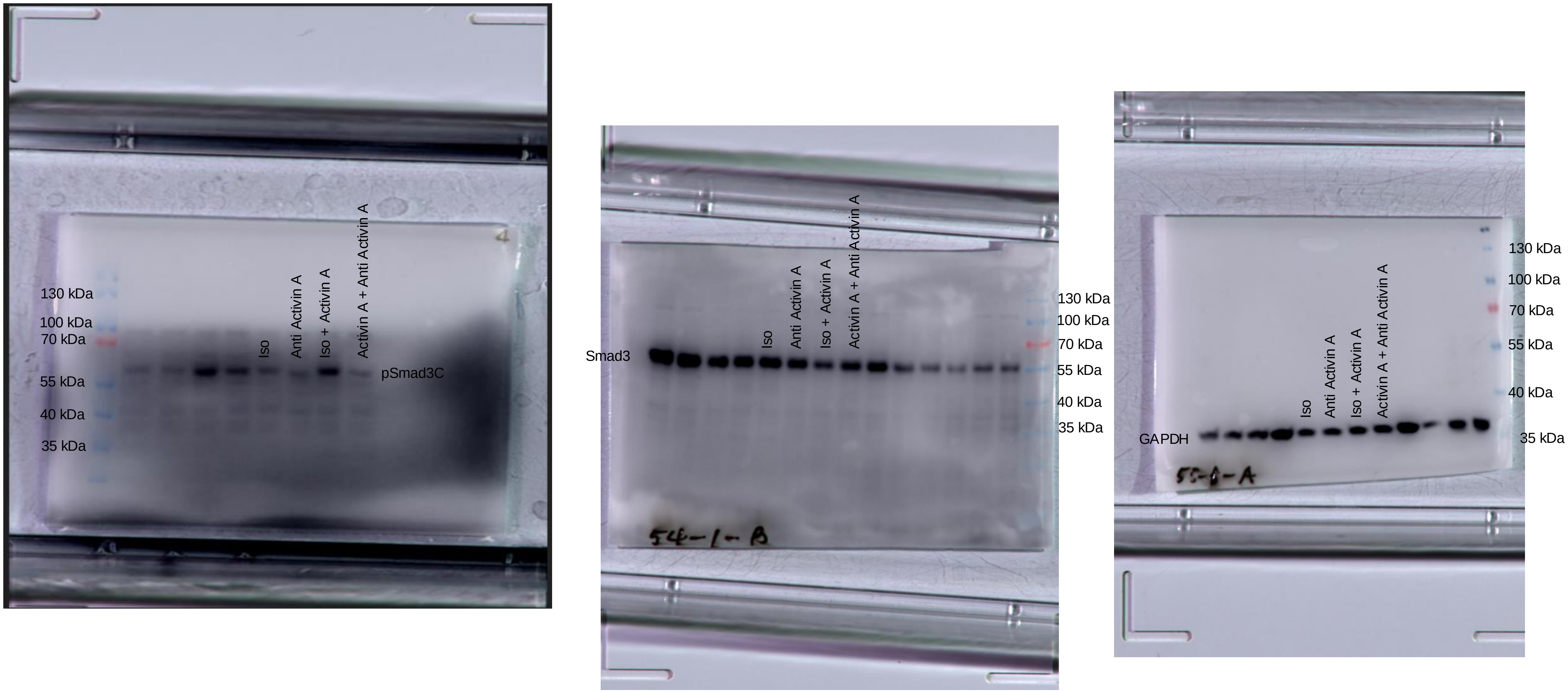
130 kDa
100 kDa
70 kDa
55 kDa
40 kDa
35 kDa
Iso
Anti Activin A
Iso + Activin A
Activin A + Anti Activin A
pSmad3C
Smad3
Iso
Anti Activin A
Iso + Activin A
Activin A + Anti Activin A
130 kDa
100 kDa
70 kDa
55 kDa
40 kDa
35 kDa
130 kDa
100 kDa
70 kDa
55 kDa
40 kDa
Iso
Anti Activin A
Iso + Activin A
Activin A + Anti Activin A
GAPDH
35 kDa

Fig S3D

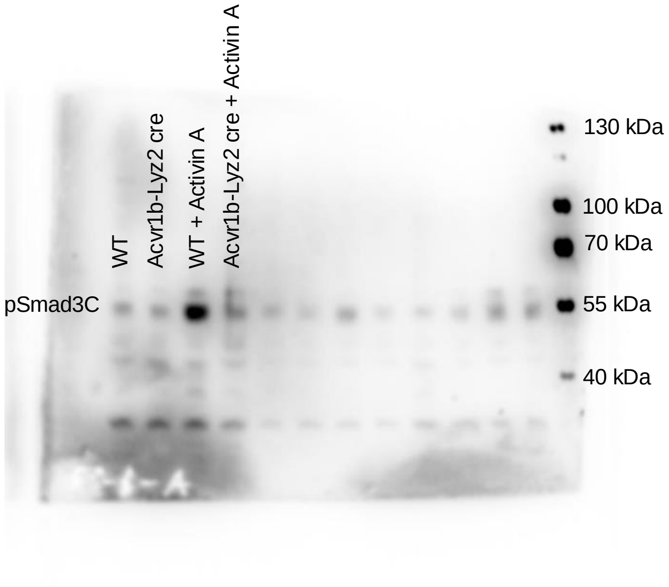

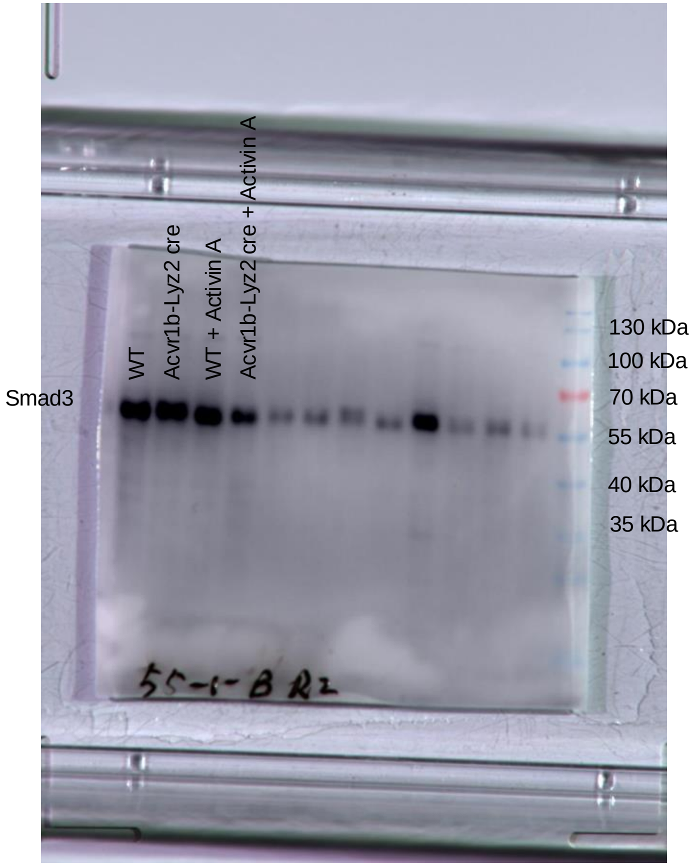

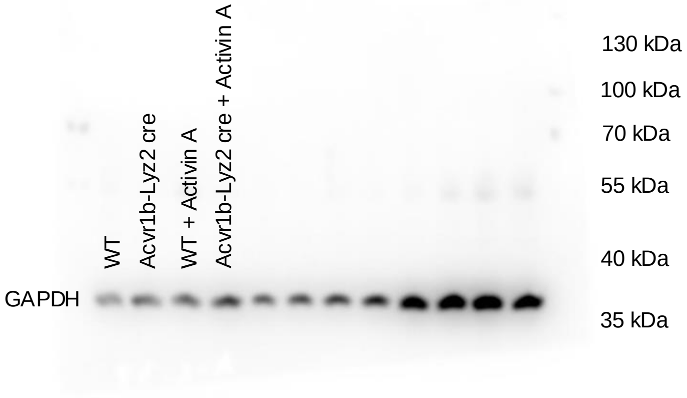

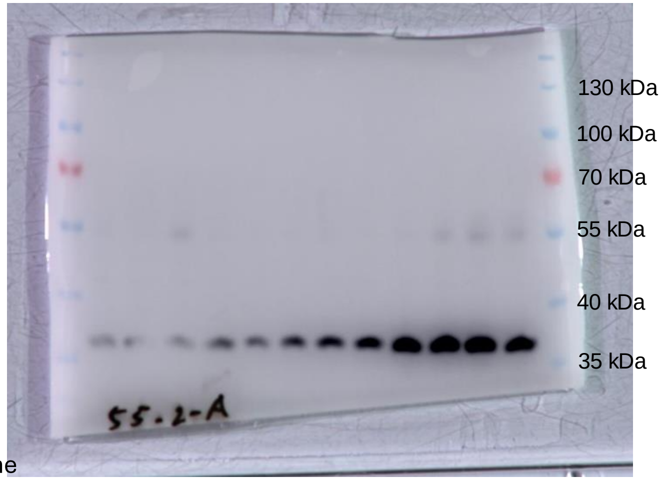

This is the same gel than above but re-exposed in order to see the marker.
Due to overexposure of the substrate some Bands started to fade.

Fig 3C

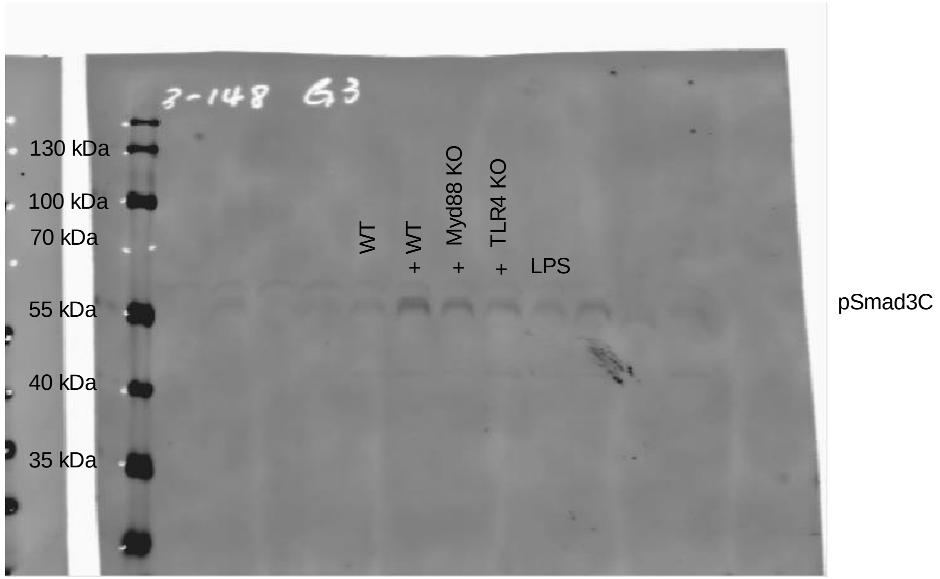
3-148 G3
130 kDa
100 kDa
70 kDa
55 kDa
40 kDa
35 kDa
WT
+ WT
+ Myd88 KO
+ TLR4 KO
LPS
pSmad3C

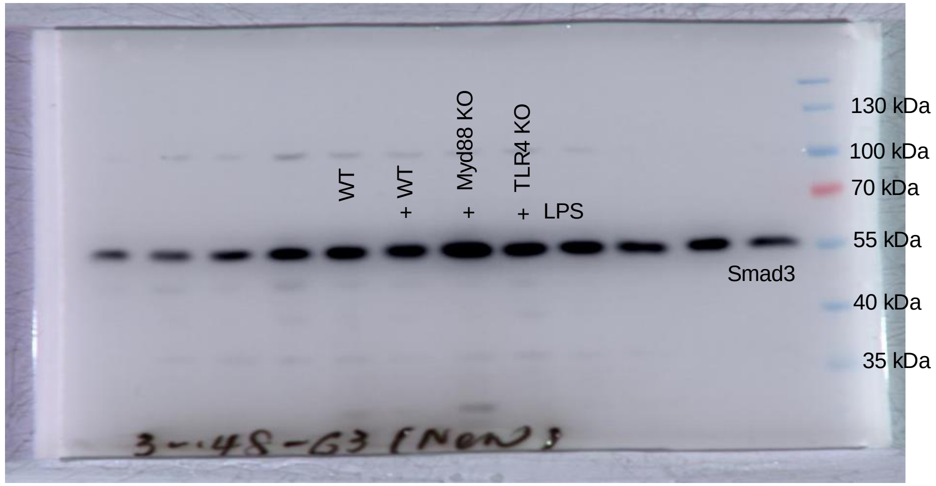
WT
+ WT
+ Myd88 KO
+ TLR4 KO
LPS
130 kDa
100 kDa
70 kDa
55 kDa
Smad3
40 kDa
35 kDa

Fig 3E

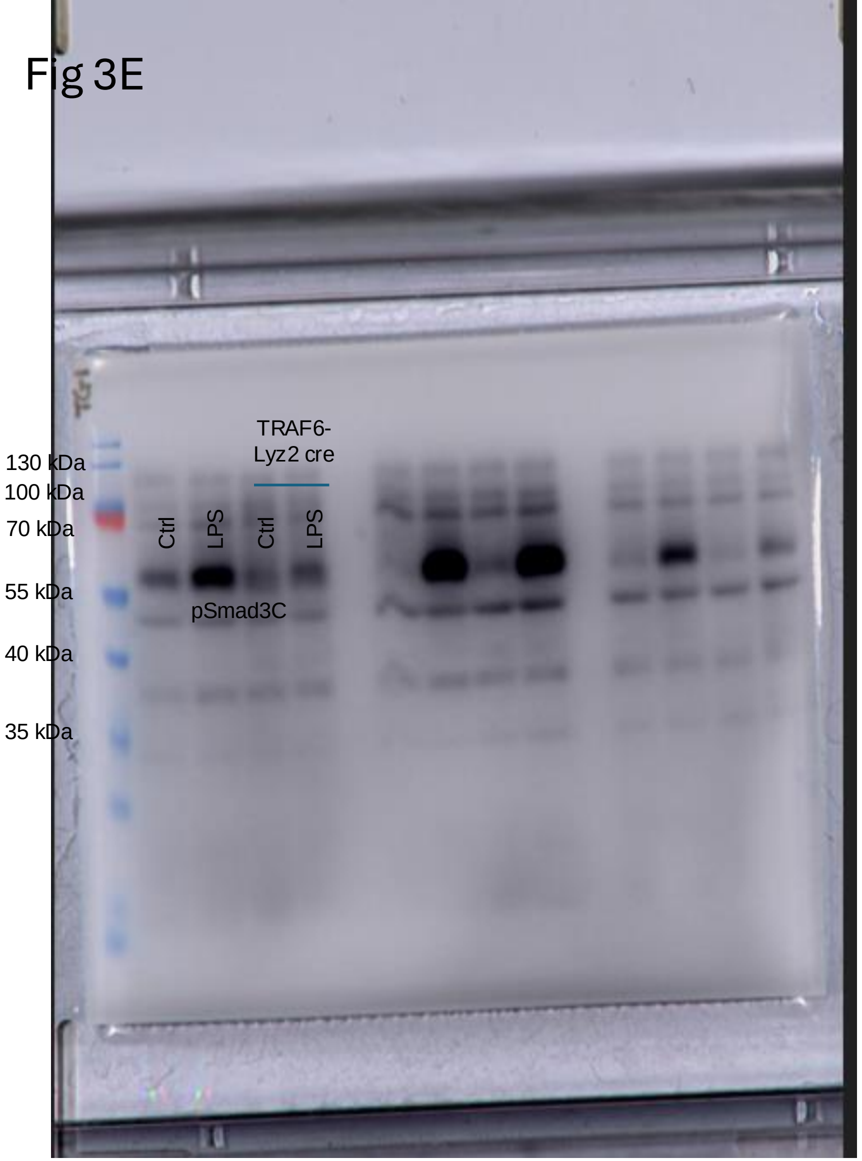
130 kDa
100 kDa
70 kDa
55 kDa
40 kDa
35 kDa
TRAF6-
Lyz2 cre
Ctrl
LPS
Ctrl
LPS
pSmad3C

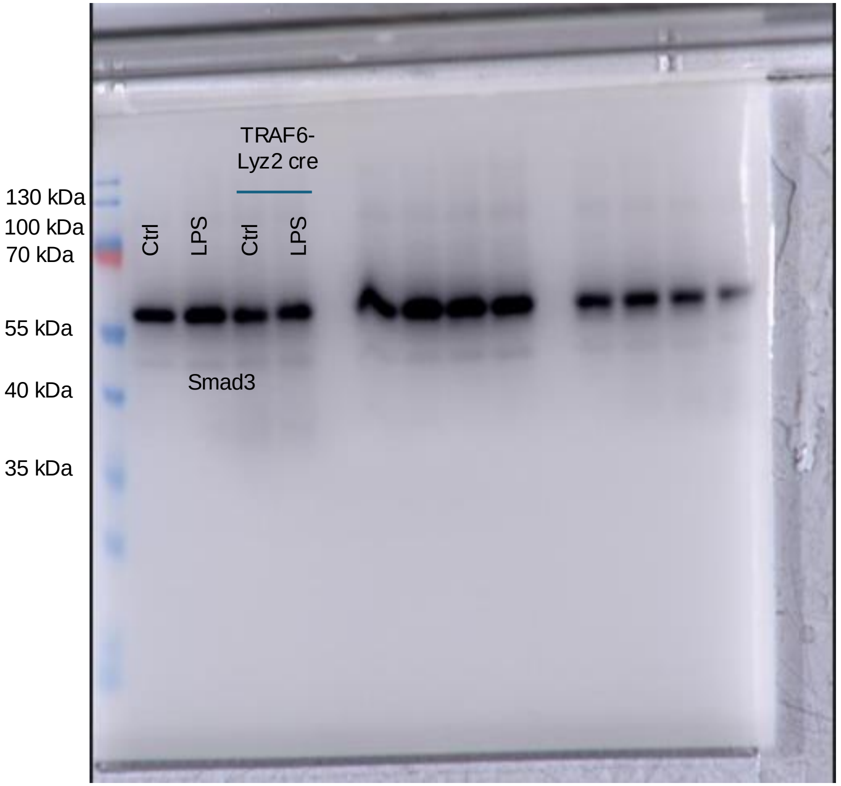
130 kDa
100 kDa
70 kDa
55 kDa
40 kDa
35 kDa
TRAF6-
Lyz2 cre
Ctrl
LPS
Ctrl
LPS
Smad3

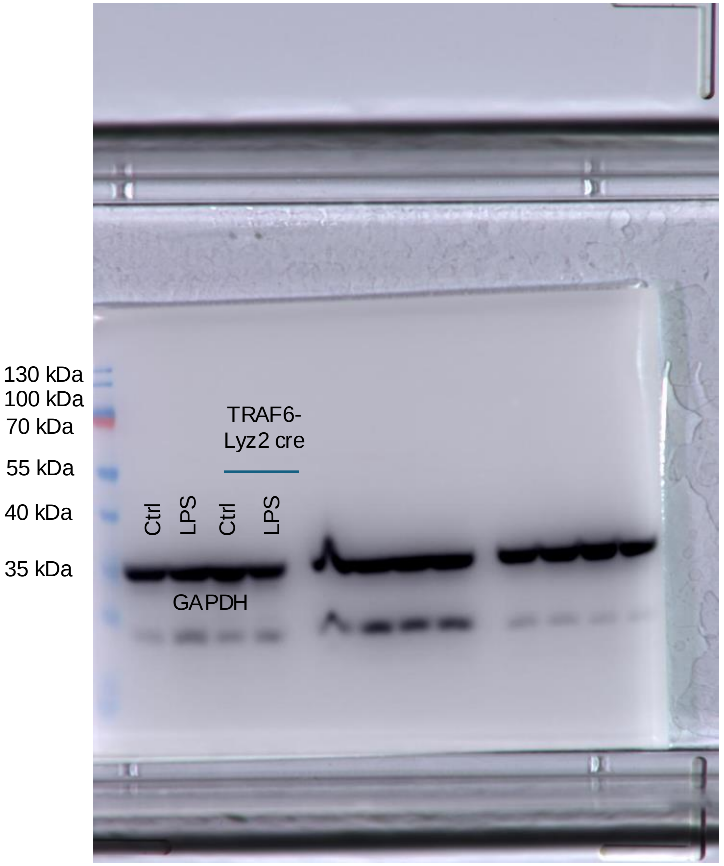
130 kDa
100 kDa
70 kDa
55 kDa
40 kDa
35 kDa
TRAF6-
Lyz2 cre
Ctrl
LPS
Ctrl
LPS
GAPDH

Fig 3G

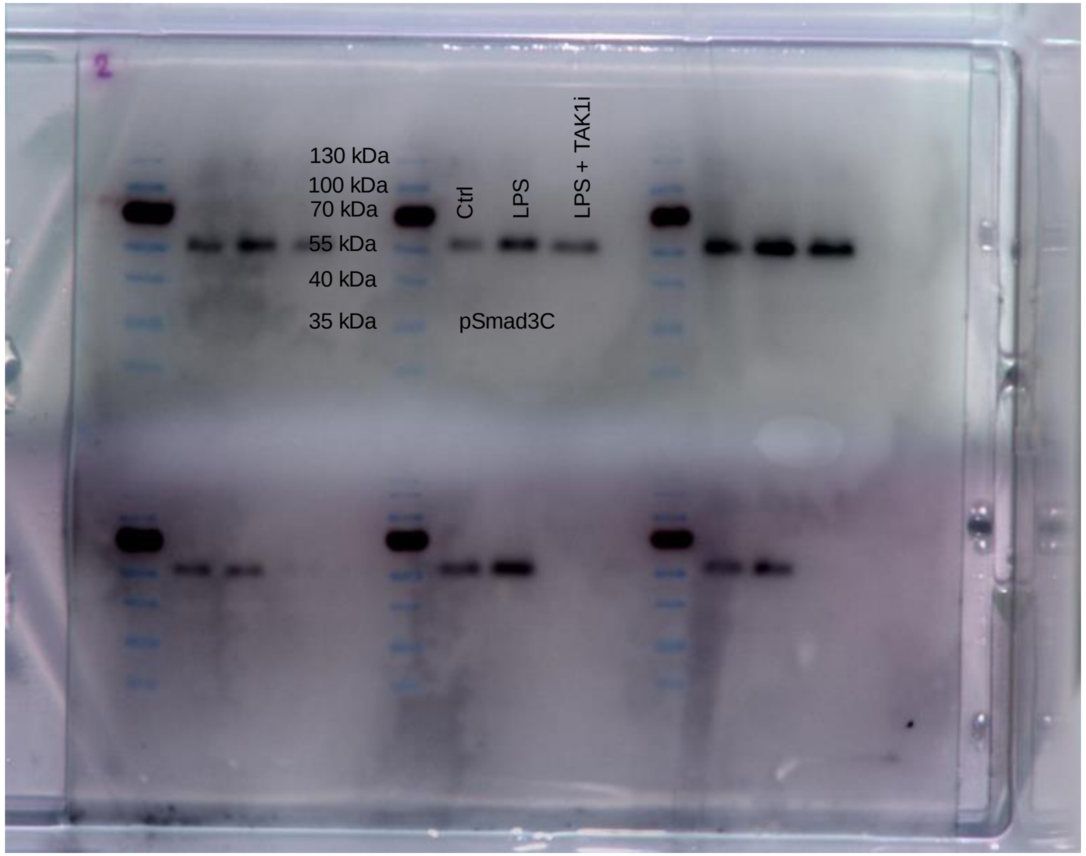

130 kDa
100 kDa
70 kDa
55 kDa
40 kDa
35 kDa
Ctrl
LPS
LPS + TAK1i
pSmad3C

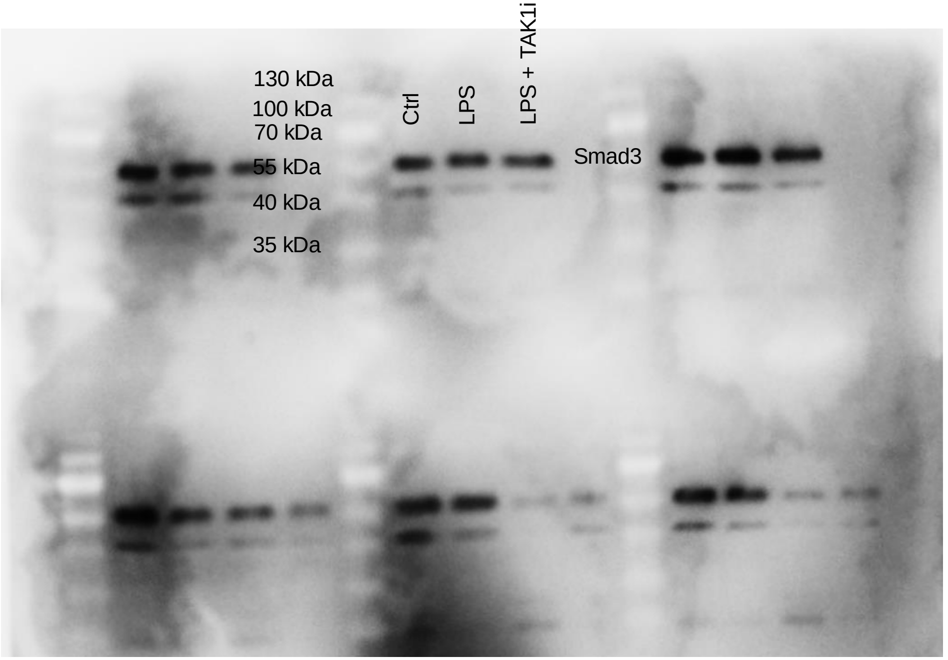

130 kDa
100 kDa
70 kDa
55 kDa
40 kDa
35 kDa
Ctrl
LPS
LPS + TAK1i
Smad3

Fig 3I

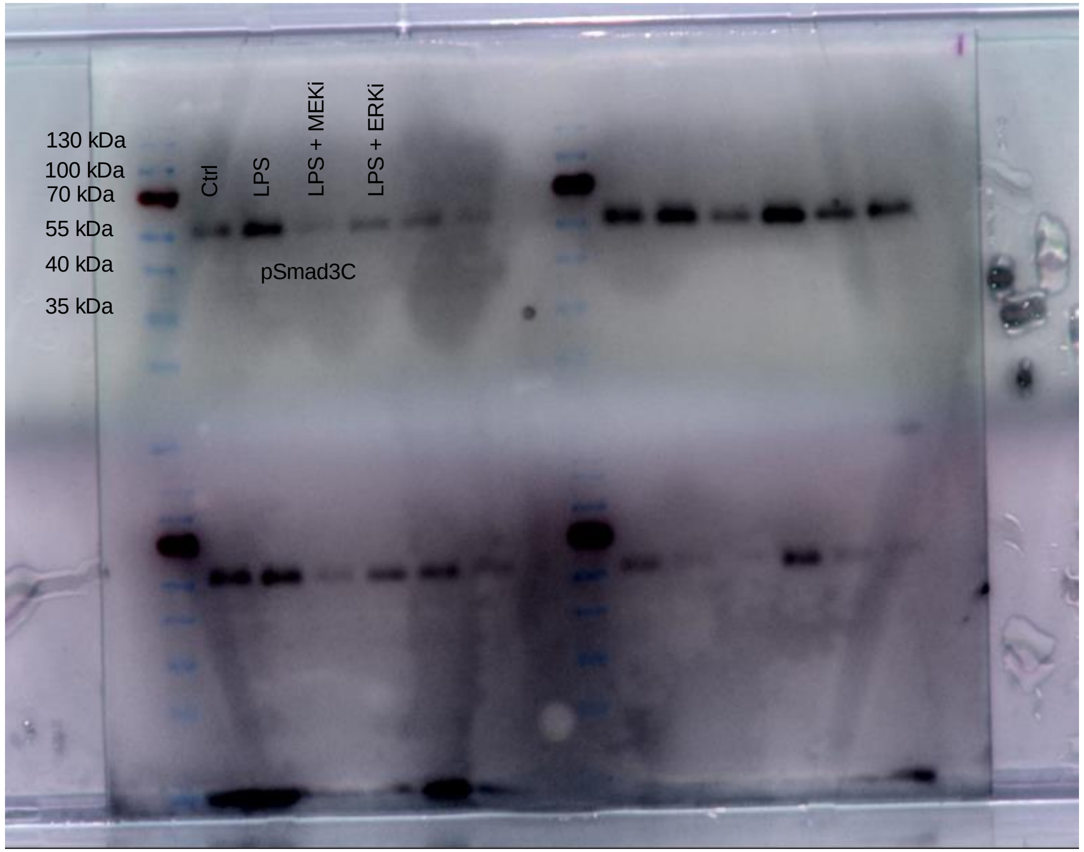

130 kDa
100 kDa
70 kDa
55 kDa
40 kDa
35 kDa
Ctrl
LPS
LPS + MEKi
LPS + ERKi
pSmad3C

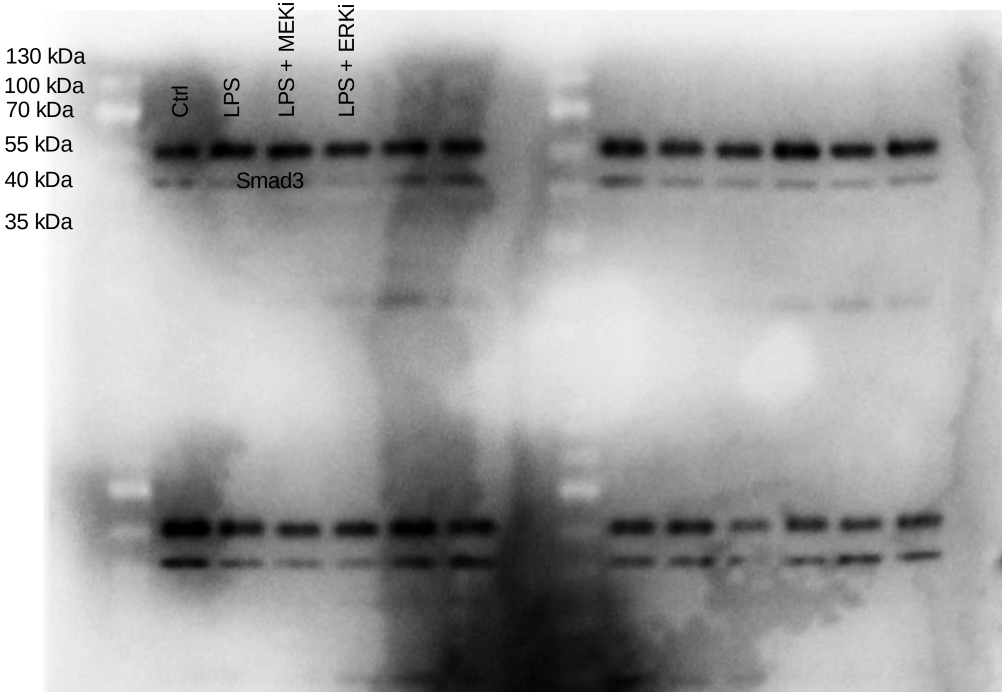

130 kDa
100 kDa
70 kDa
55 kDa
40 kDa
35 kDa
Ctrl
LPS
LPS + MEKi
LPS + ERKi
Smad3

Fig 3J

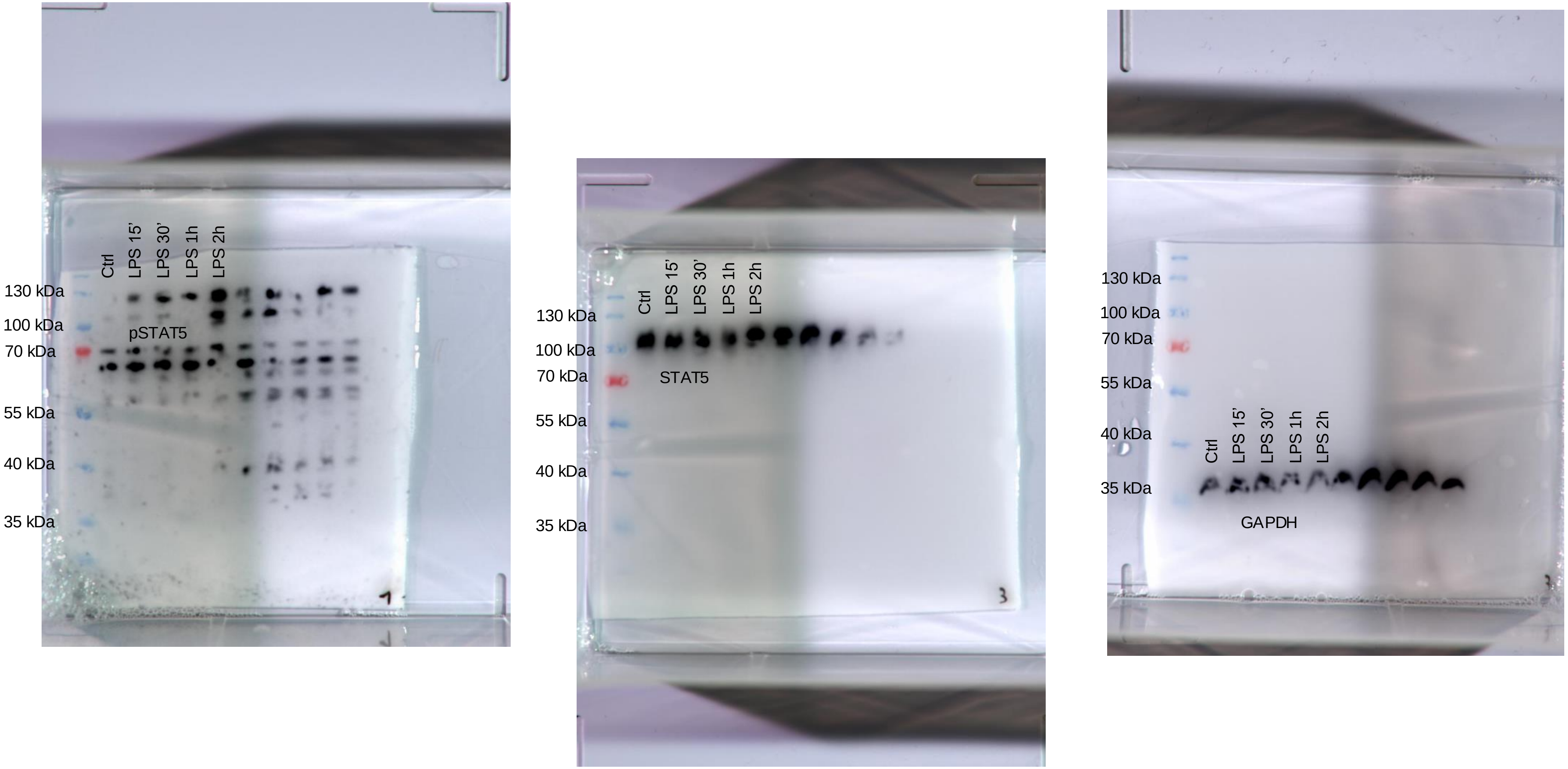
Ctrl
LPS 15'
LPS 30'
LPS 1h
LPS 2h
130 kDa
100 kDa
70 kDa
55 kDa
40 kDa
35 kDa
pSTAT5
Ctrl
LPS 15'
LPS 30'
LPS 1h
LPS 2h
130 kDa
100 kDa
70 kDa
55 kDa
40 kDa
35 kDa
STAT5
130 kDa
100 kDa
70 kDa
55 kDa
40 kDa
35 kDa
Ctrl
LPS 15'
LPS 30'
LPS 1h
LPS 2h
GAPDH

Fig 3M

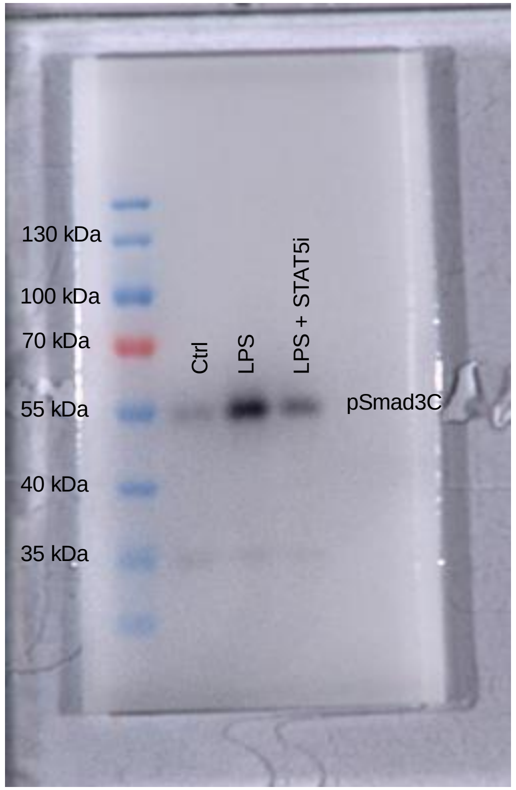

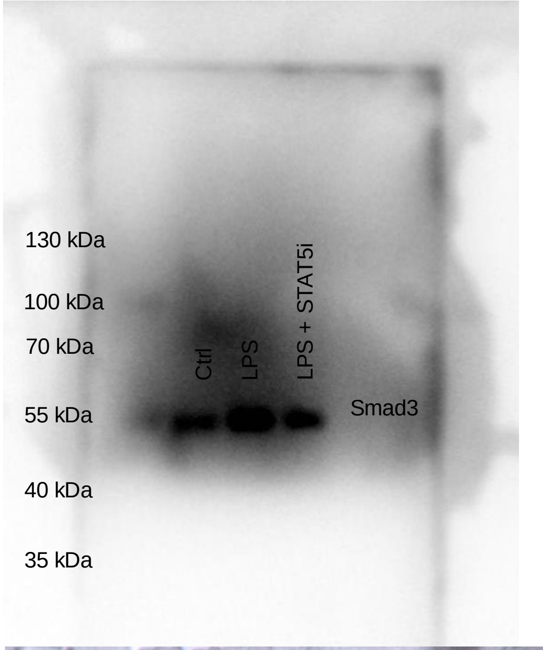

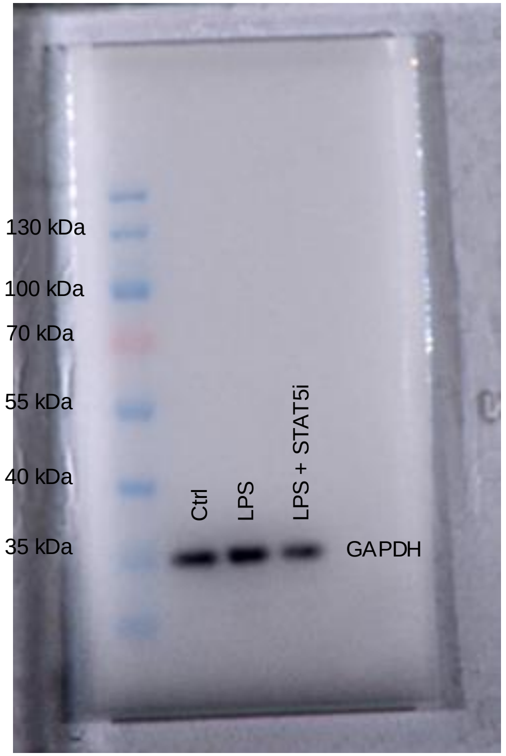

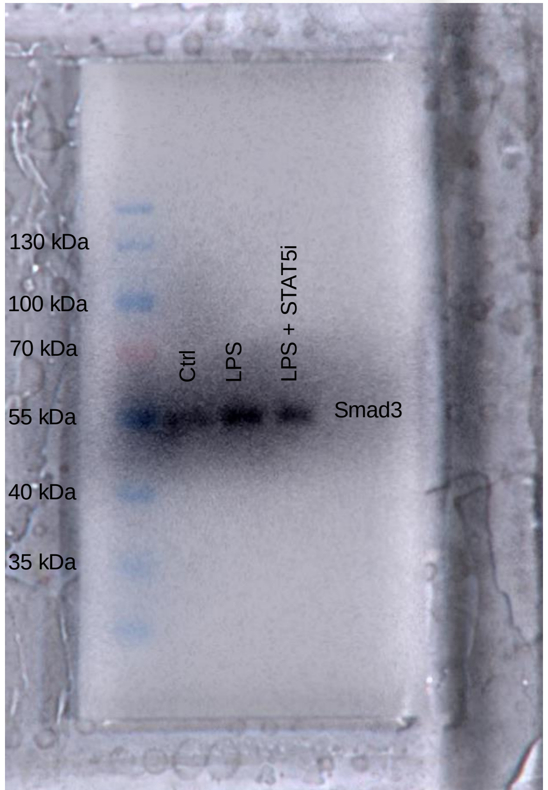

This is the same gel than above but re-exposed in order to see the marker.
Due to high background we used the upper gel for publication

Fig 3O

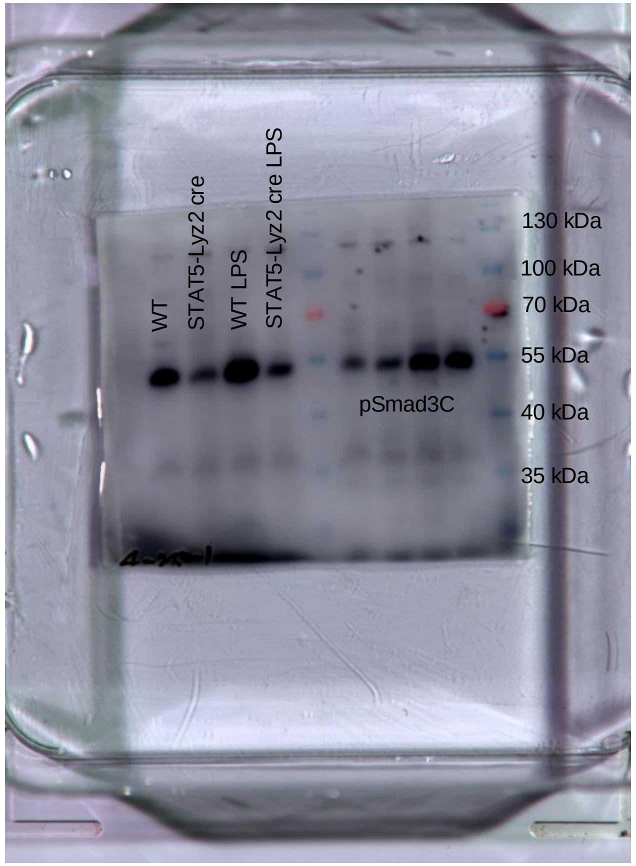
WT
STAT5-Lyz2 cre
WT LPS
STAT5-Lyz2 cre LPS
130 kDa
100 kDa
70 kDa
55 kDa
pSmad3C
40 kDa
35 kDa

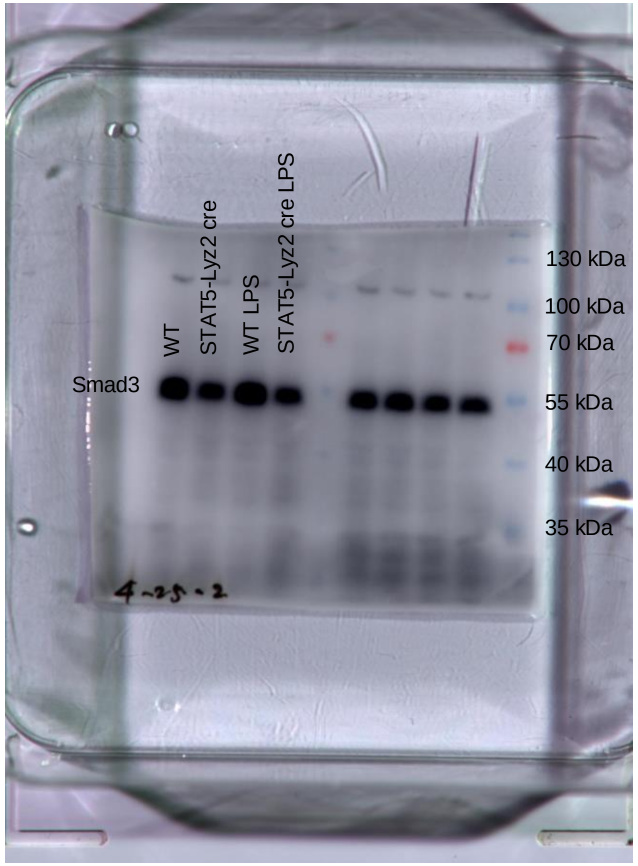
WT
STAT5-Lyz2 cre
WT LPS
STAT5-Lyz2 cre LPS
130 kDa
100 kDa
70 kDa
Smad3
55 kDa
40 kDa
35 kDa

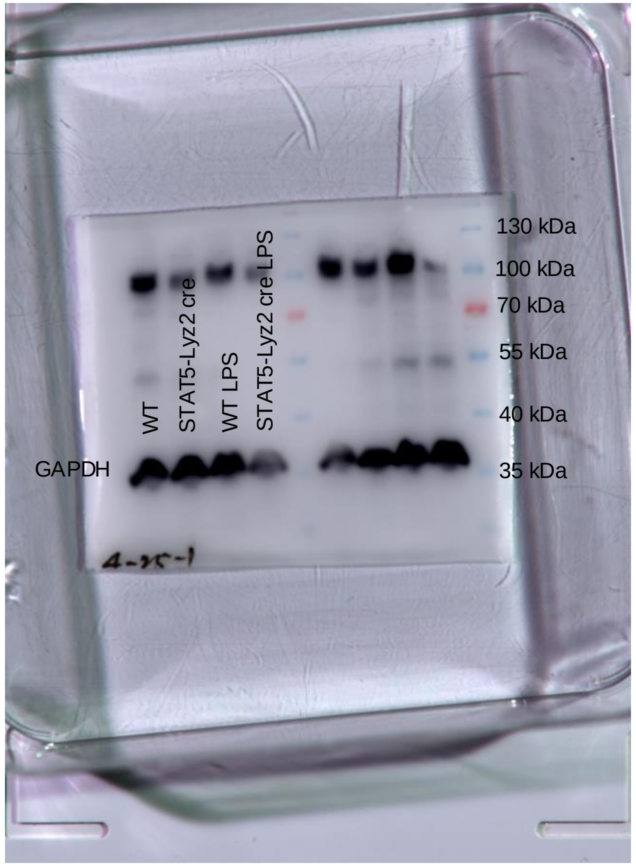
130 kDa
100 kDa
70 kDa
55 kDa
WT
STAT5-Lyz2 cre
WT LPS
STAT5-Lyz2 cre LPS
40 kDa
GAPDH
35 kDa

Fig S4A

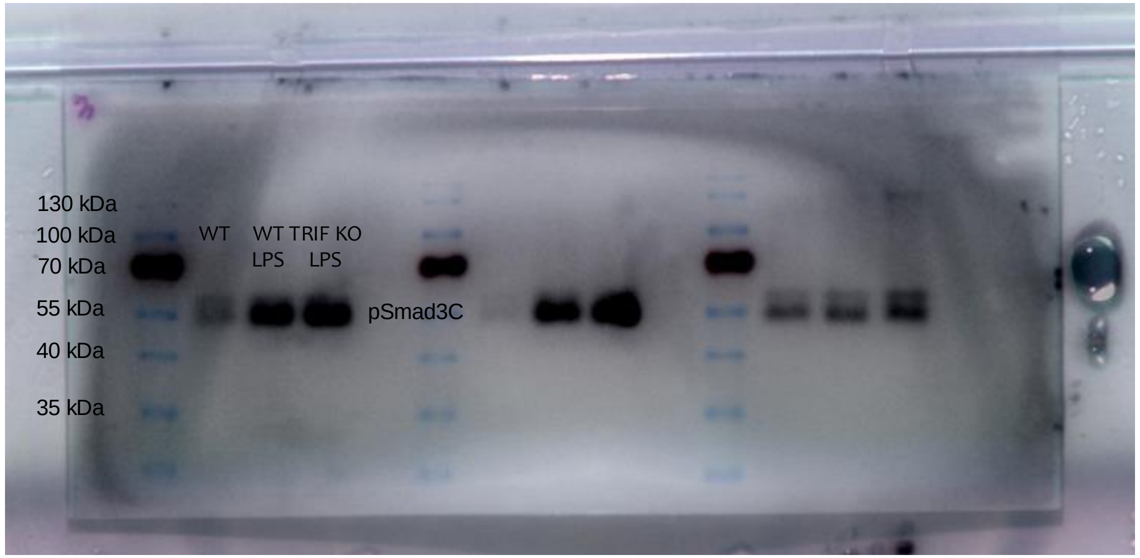

130 kDa
100 kDa
70 kDa
55 kDa
40 kDa
35 kDa
WT
WT LPS
TRIF KO LPS
pSmad3C

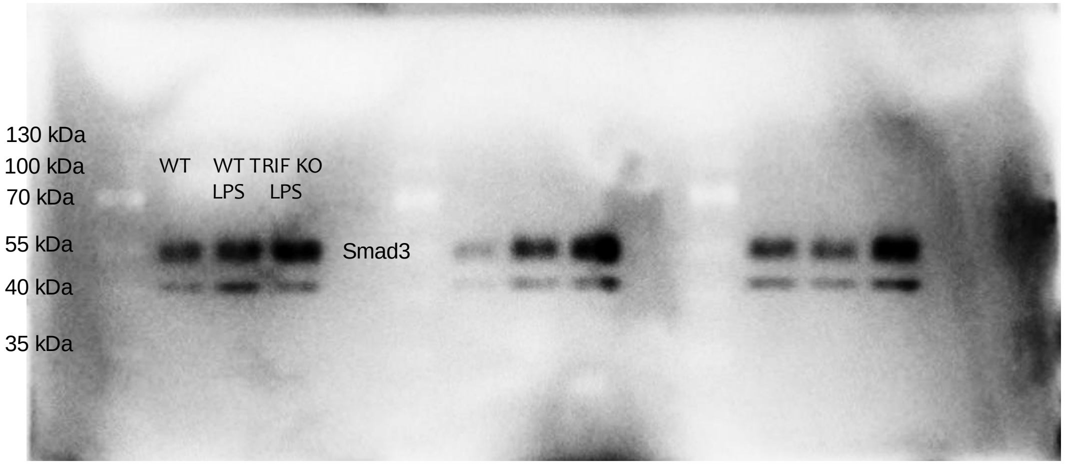

130 kDa
100 kDa
70 kDa
55 kDa
40 kDa
35 kDa
WT
WT LPS
TRIF KO LPS
Smad3

Fig S4D

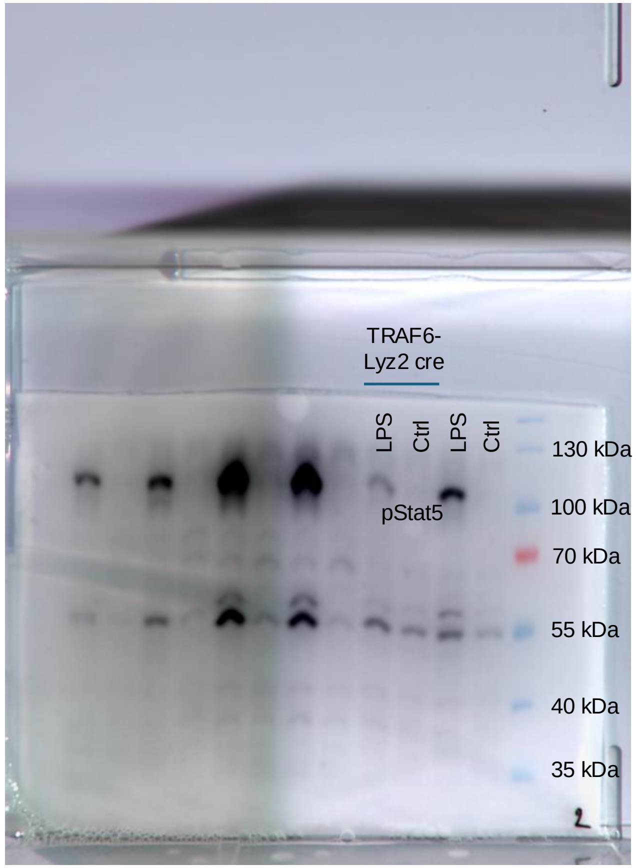
TRAF6-
Lyz2 cre
LPS
Ctrl
LPS
Ctrl
130 kDa
100 kDa
pStat5
70 kDa
55 kDa
40 kDa
35 kDa

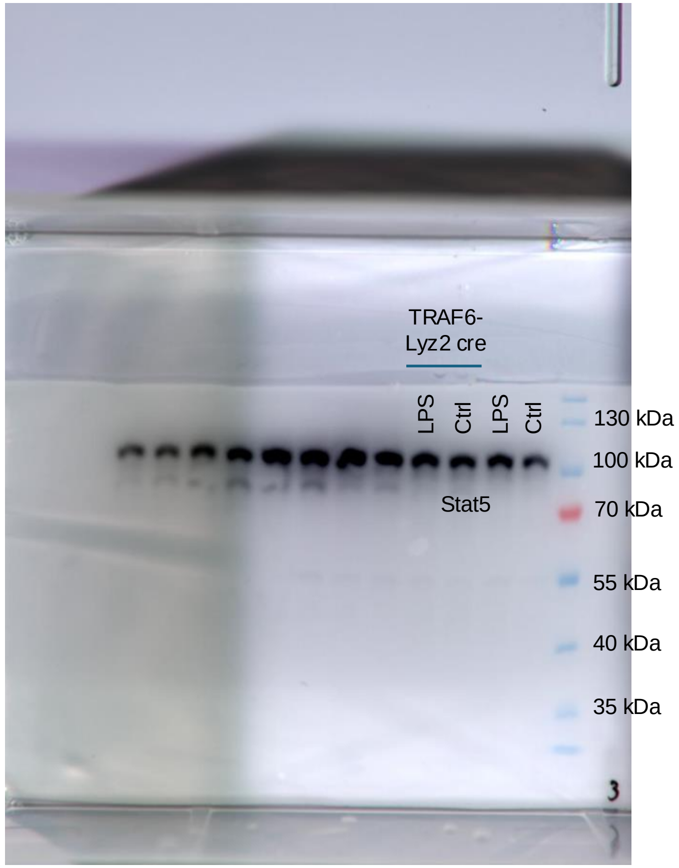
TRAF6-
Lyz2 cre
LPS
Ctrl
LPS
Ctrl
130 kDa
100 kDa
Stat5
70 kDa
55 kDa
40 kDa
35 kDa

Fig S4E

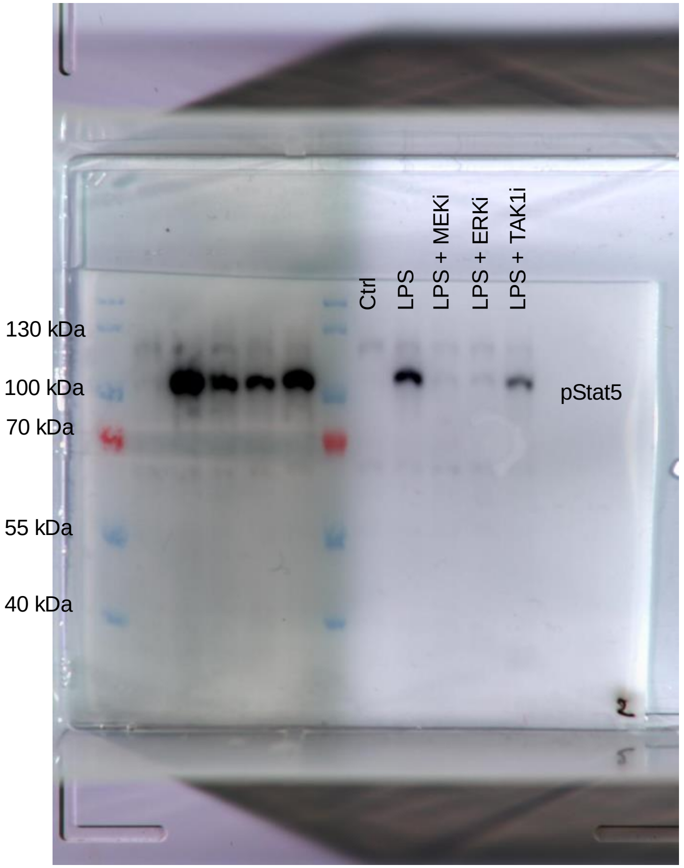

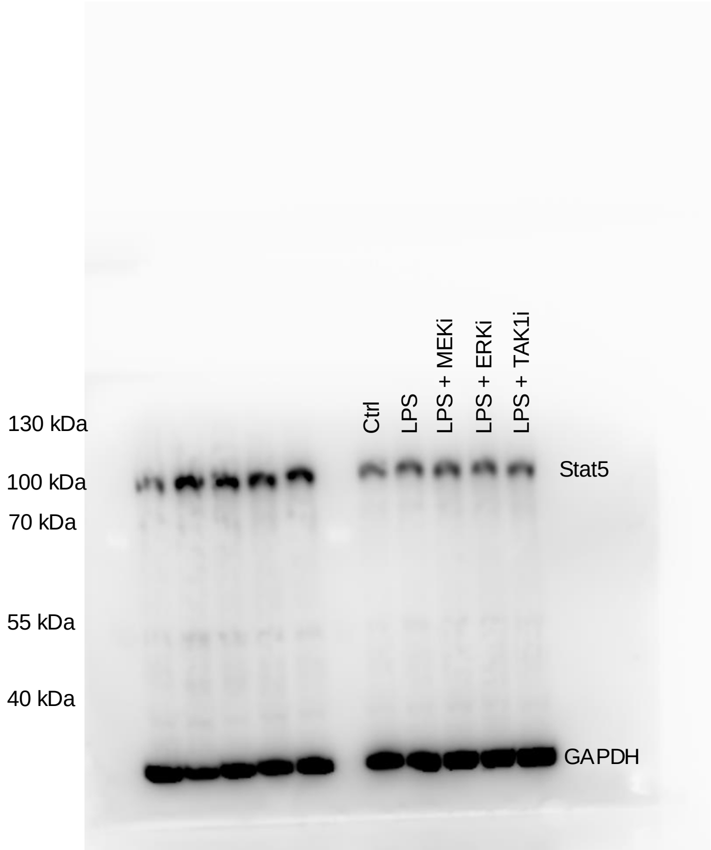

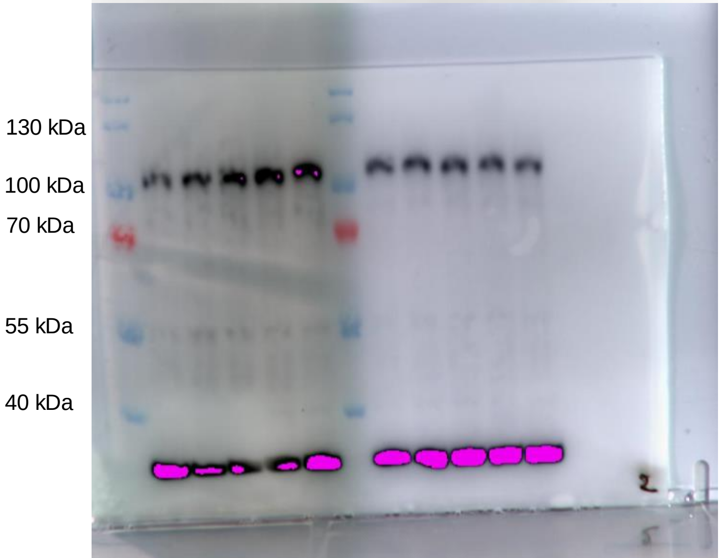

This is the same gel than above but re-exposed in order to see the marker.
Due to saturation of the bands we used the above gel for publication.

Fig S5A

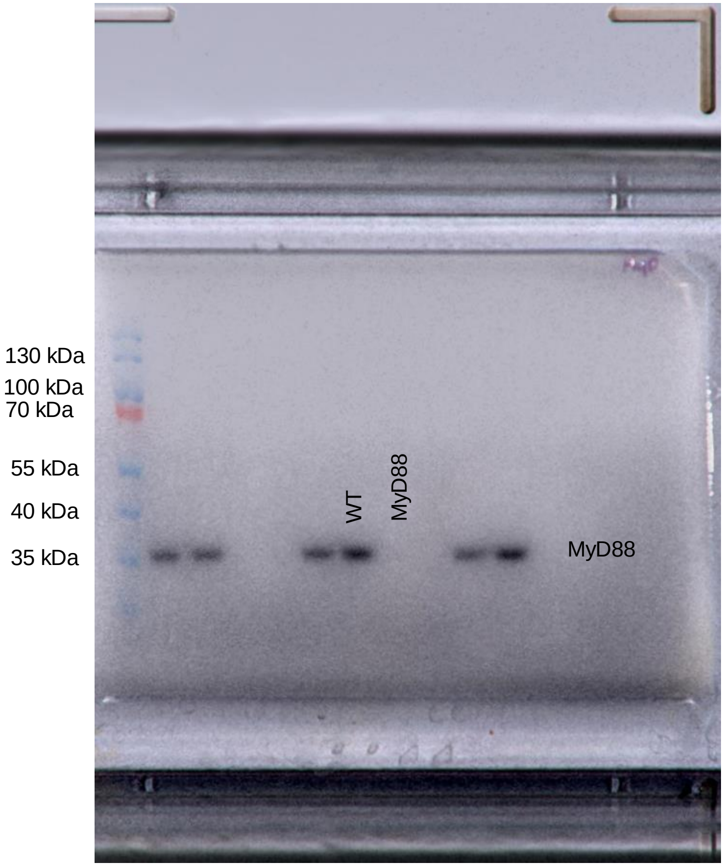
130 kDa
100 kDa
70 kDa
55 kDa
40 kDa
35 kDa
WT
MyD88
MyD88

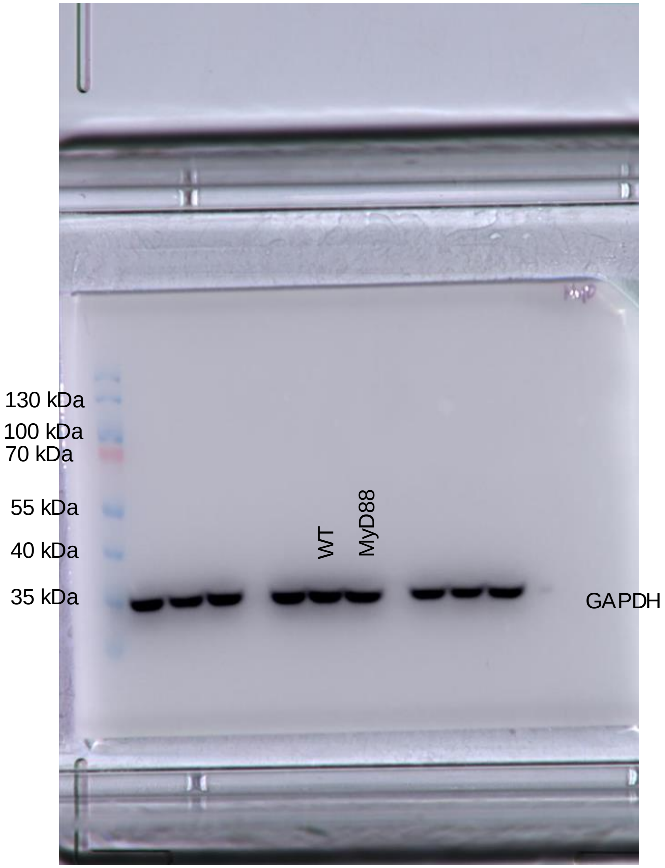
130 kDa
100 kDa
70 kDa
55 kDa
40 kDa
35 kDa
WT
MyD88
GAPDH

Fig S5B

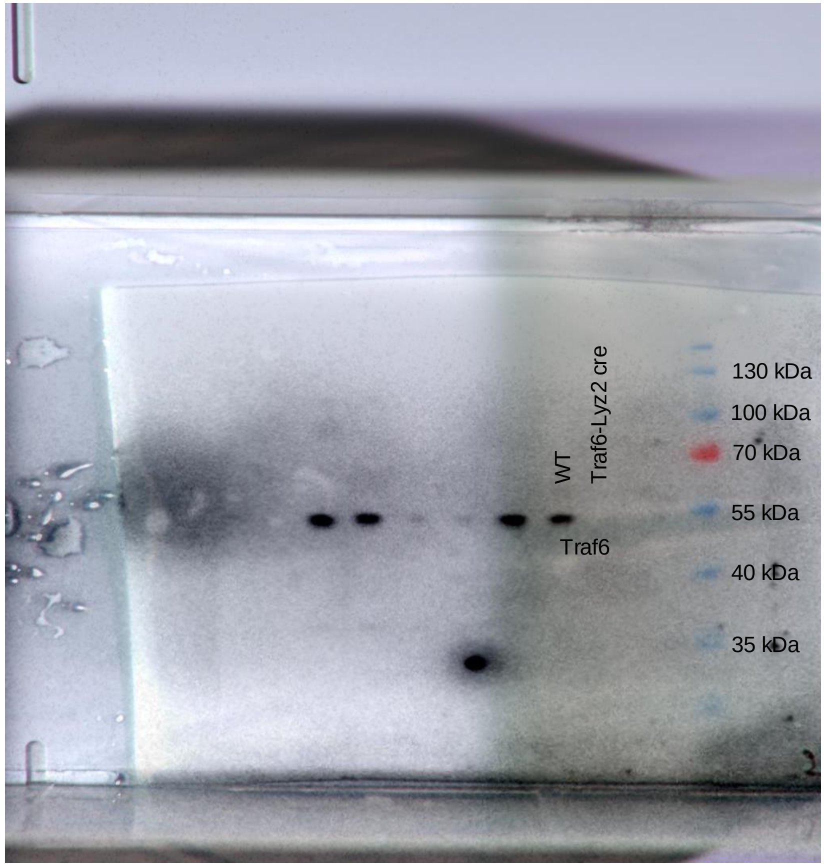
WT
Traf6-Lyz2 cre
130 kDa
100 kDa
70 kDa
55 kDa
Traf6
40 kDa
35 kDa

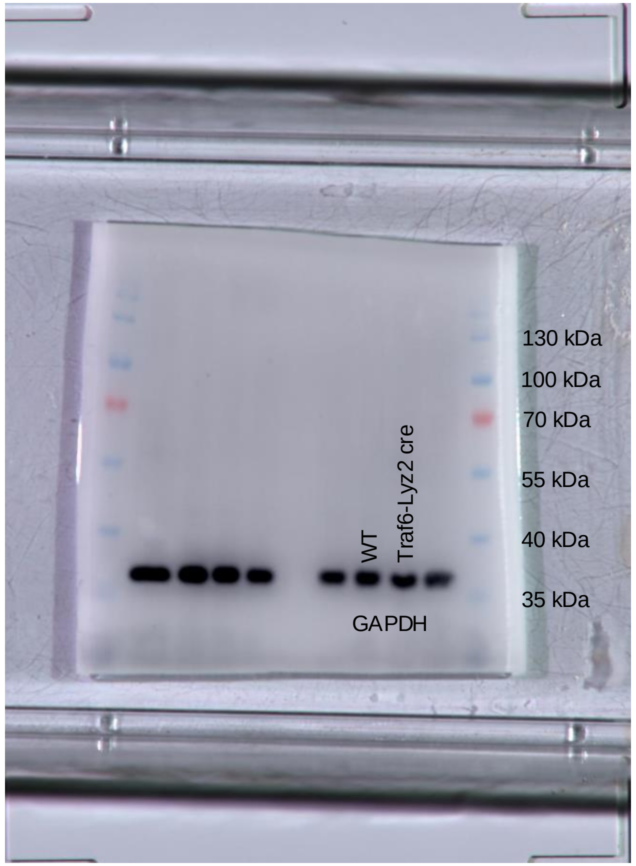
130 kDa
100 kDa
70 kDa
WT
Traf6-Lyz2 cre
55 kDa
40 kDa
35 kDa
GAPDH

Fig S5C

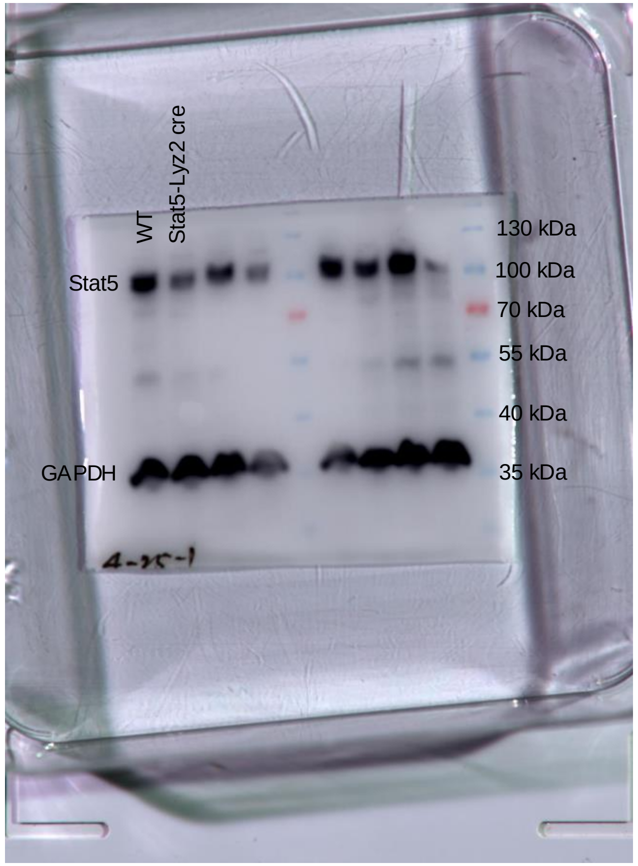
WT
Stat5-Lyz2 cre
Stat5
GAPDH
130 kDa
100 kDa
70 kDa
55 kDa
40 kDa
35 kDa

Fig S5D

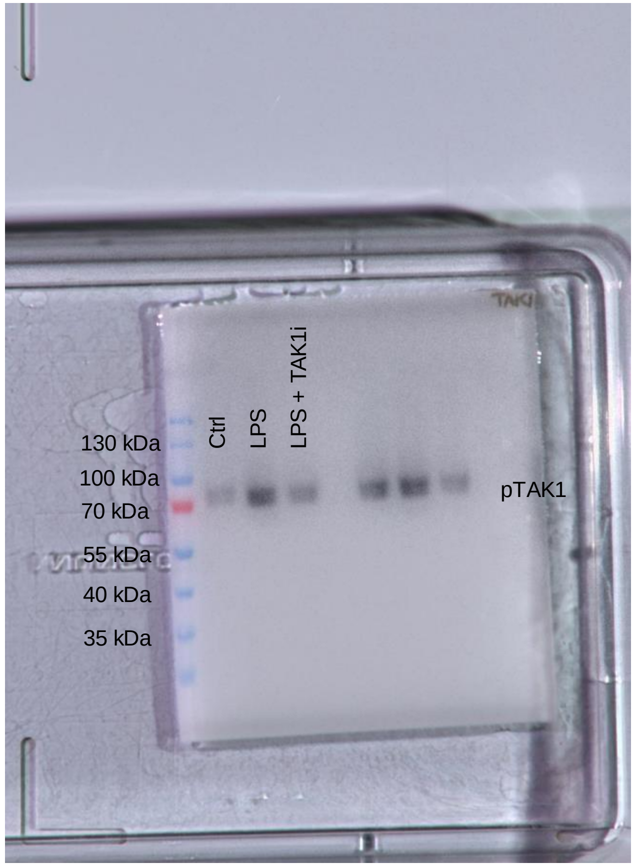
Ctrl
LPS
LPS + TAK1i
130 kDa
100 kDa
70 kDa
55 kDa
40 kDa
35 kDa
pTAK1

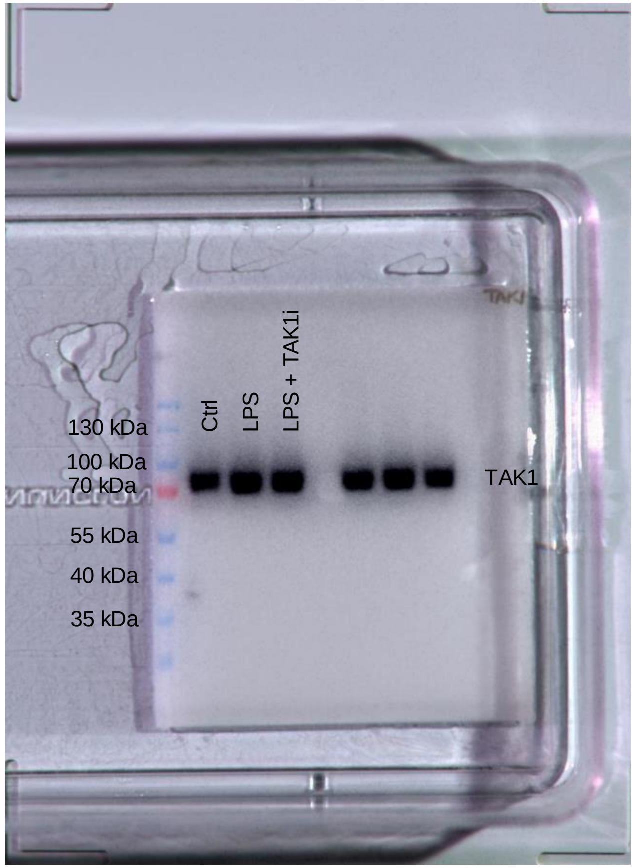
Ctrl
LPS
LPS + TAK1i
130 kDa
100 kDa
70 kDa
55 kDa
40 kDa
35 kDa
TAK1

Fig S5E

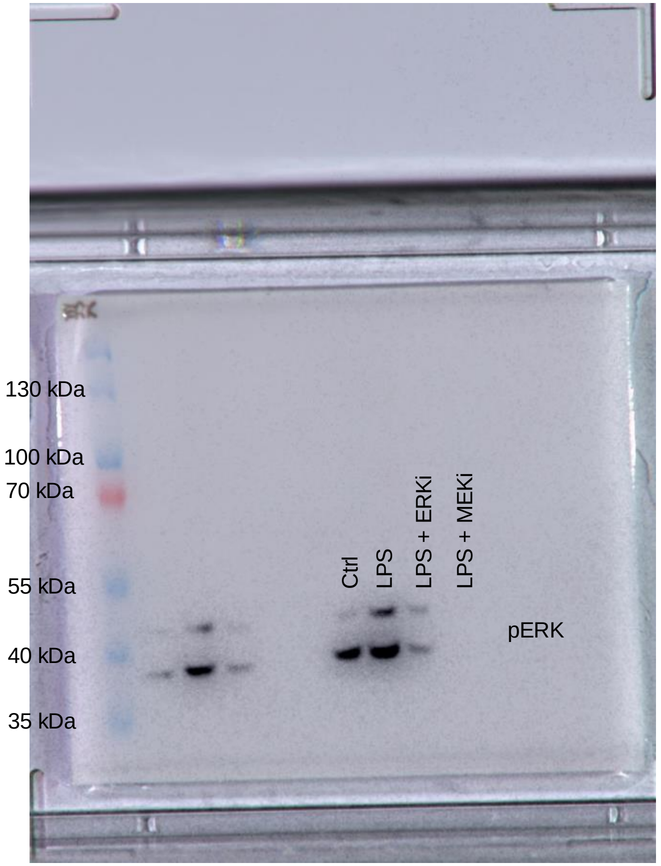

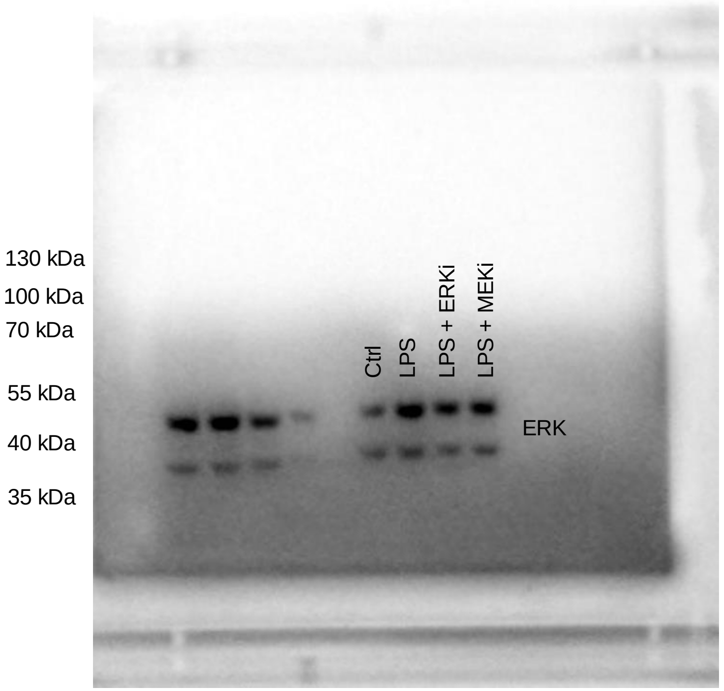

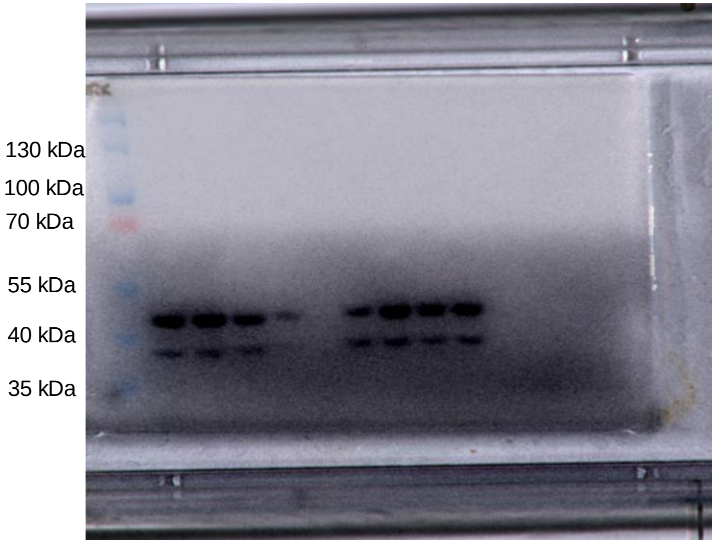

This is the same gel than above but re-exposed in order to see the marker. Due to high background, we used the above gel for publication.

Fig S5F

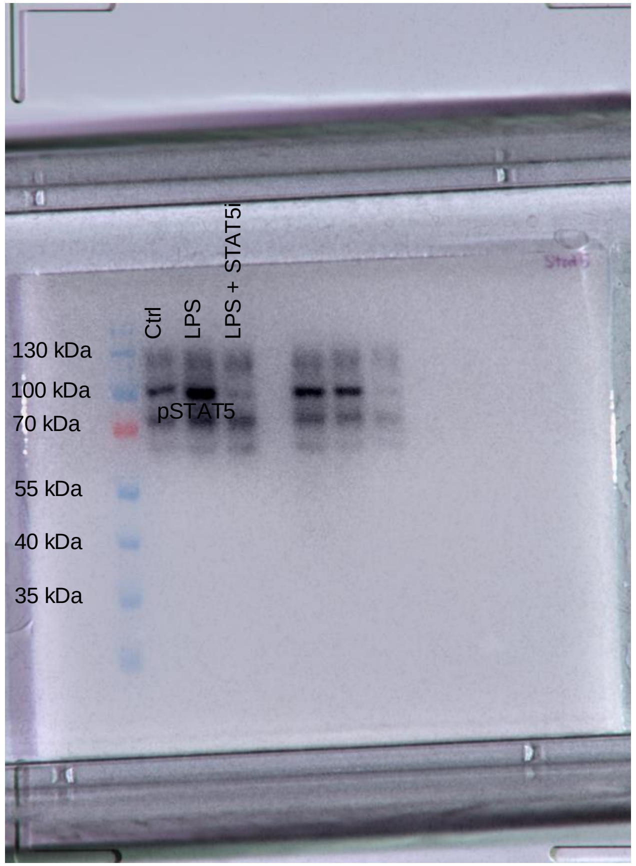
Ctrl
LPS
LPS + STAT5i
130 kDa
100 kDa
70 kDa
pSTAT5
55 kDa
40 kDa
35 kDa

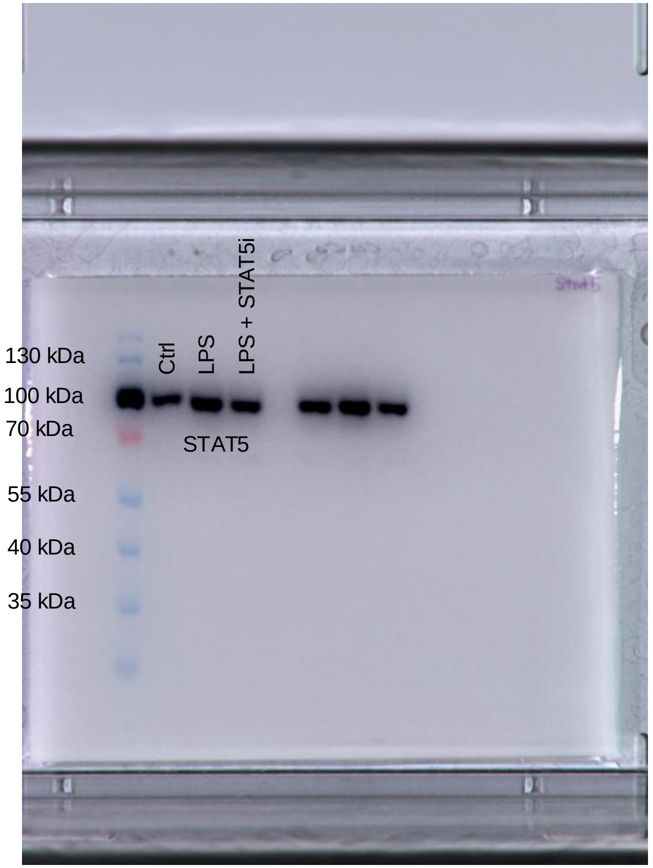
Ctrl
LPS
LPS + STAT5i
130 kDa
100 kDa
70 kDa
STAT5
55 kDa
40 kDa
35 kDa

Fig S10B

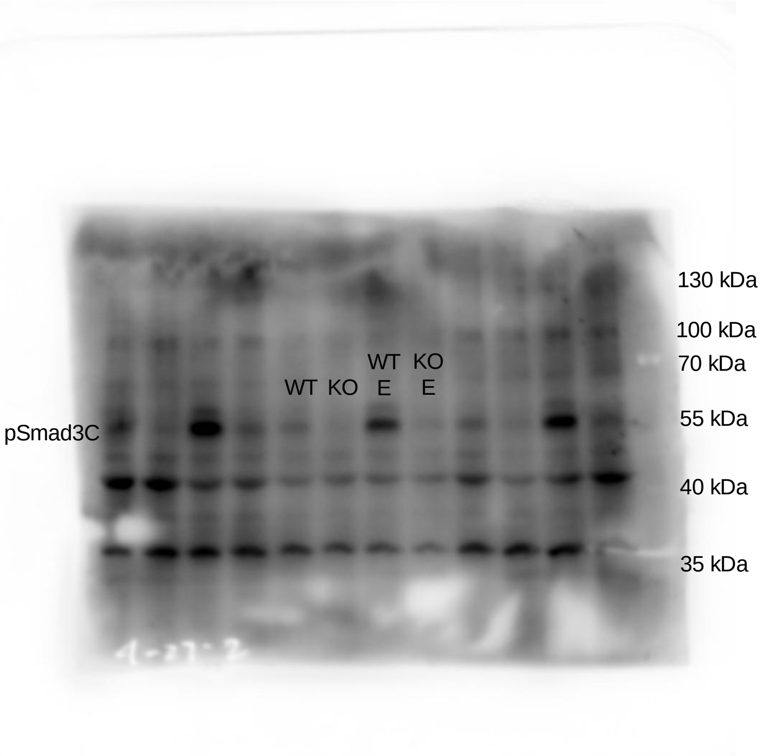

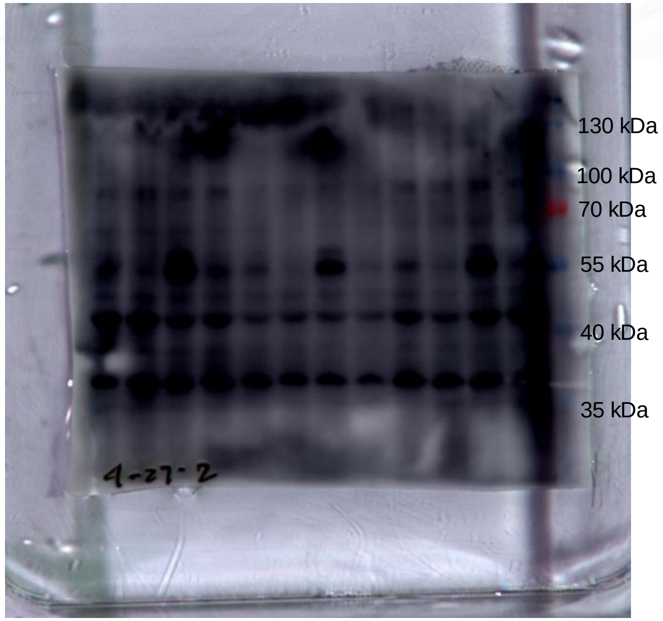

This is the same gel than above but re-exposed in order to see the marker. Due to high background, we used the above gel for publication.

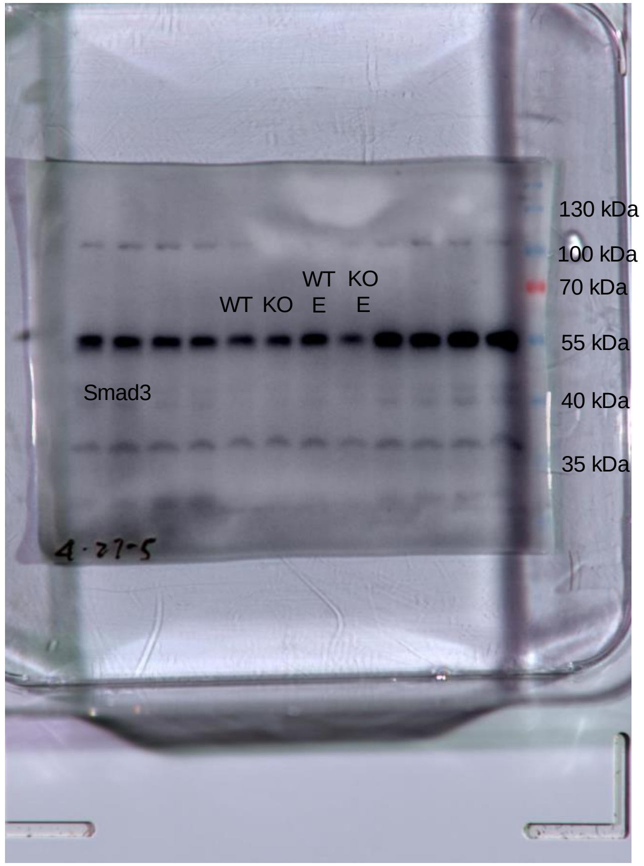

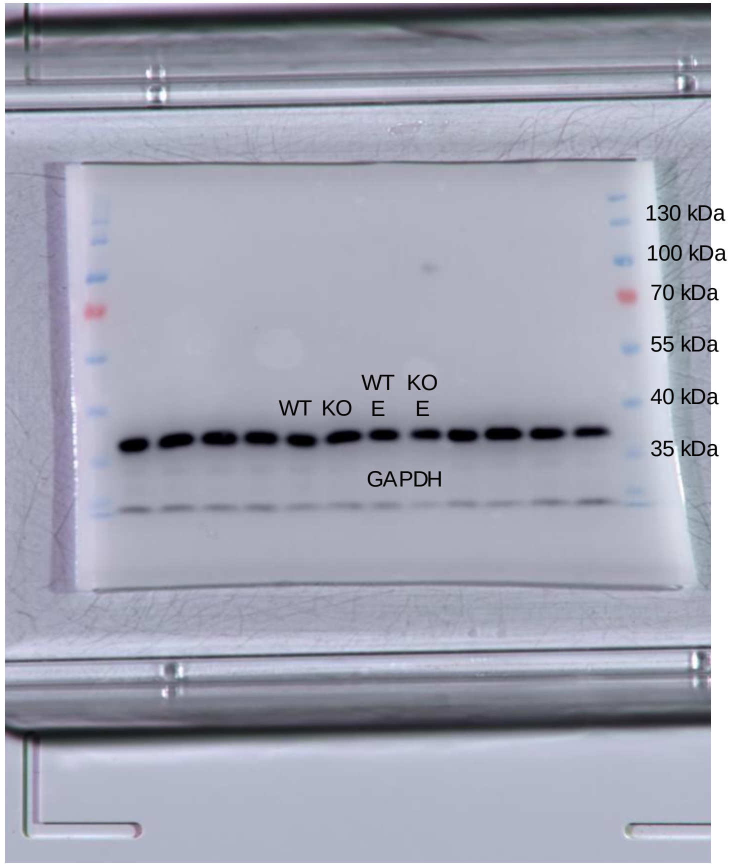

Fig S10D

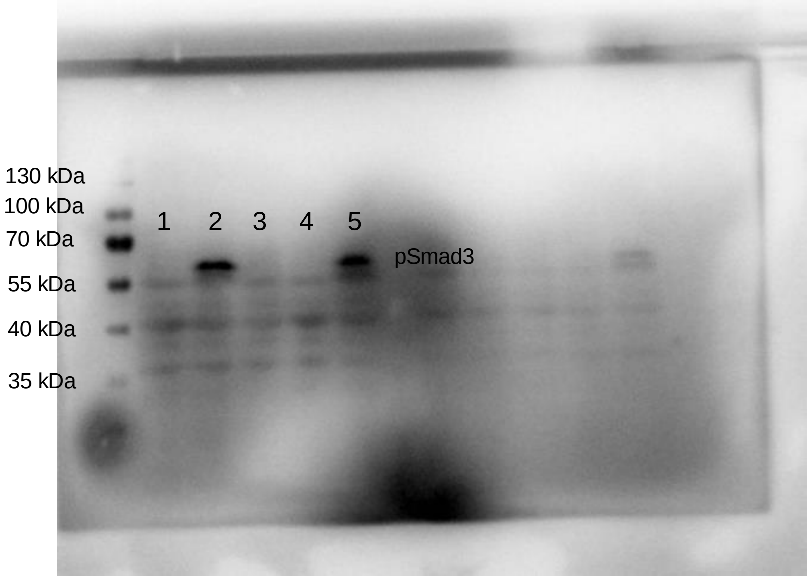

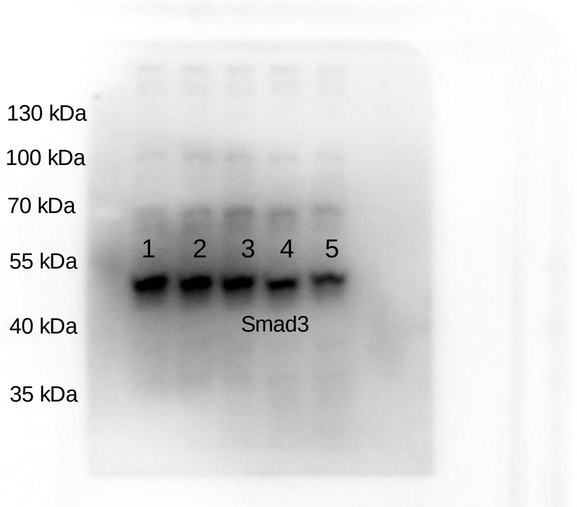

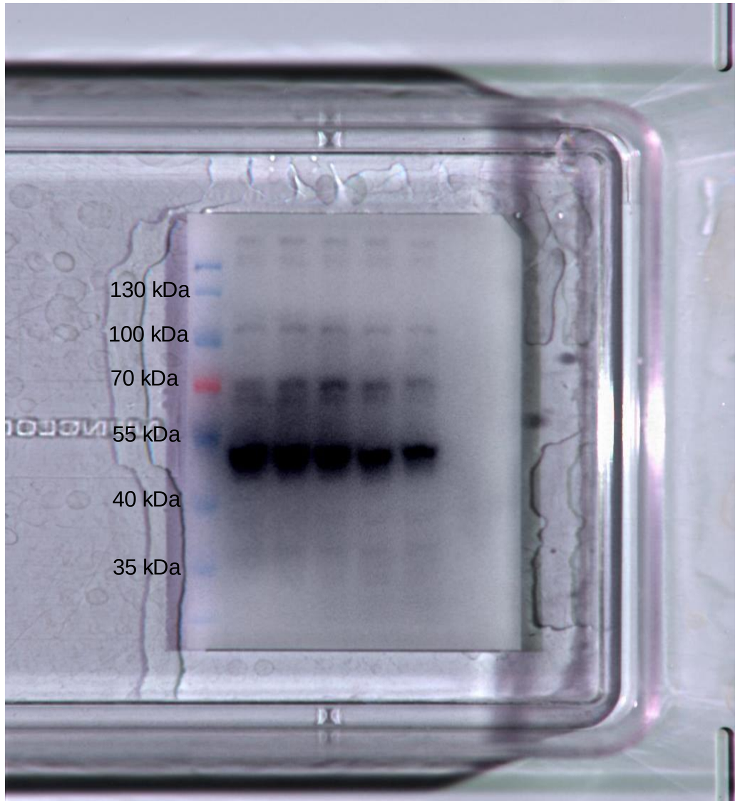

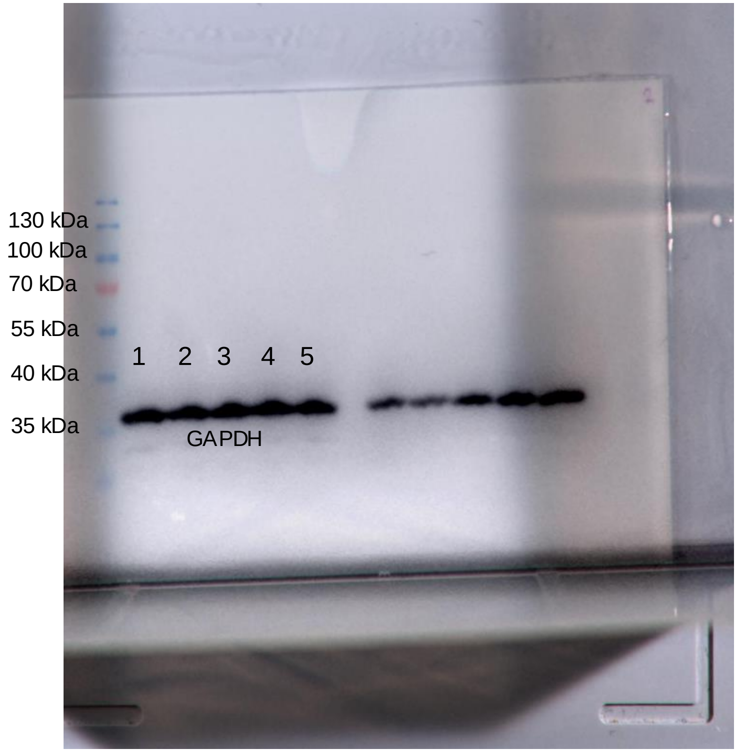

1: Ctrl
2: E protein
3: Follistatin + E protein
4: Anti Activin A + E protein
5: Anti TGF-β + E protein

This is the same gel than above but re-exposed in order to see the marker. Due to high background, we used the above gel for publication.

Fig 6B

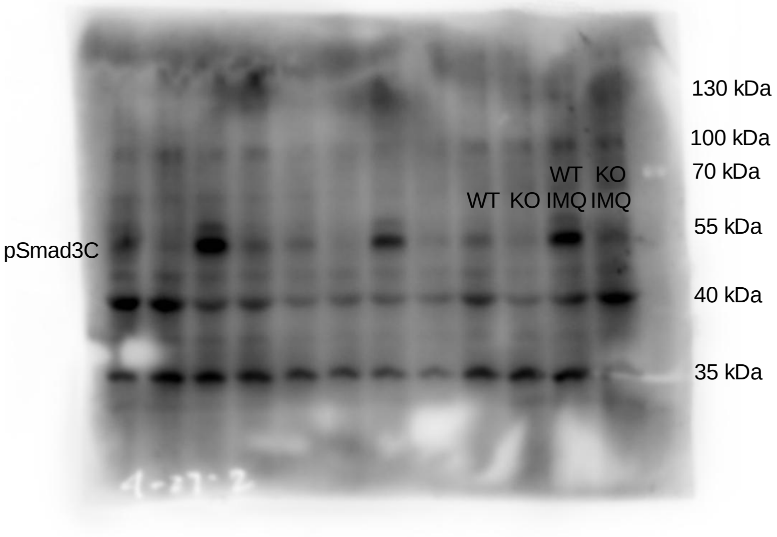
130 kDa
100 kDa
70 kDa
WT KO
WT KO IMQ IMQ
55 kDa
pSmad3C
40 kDa
35 kDa

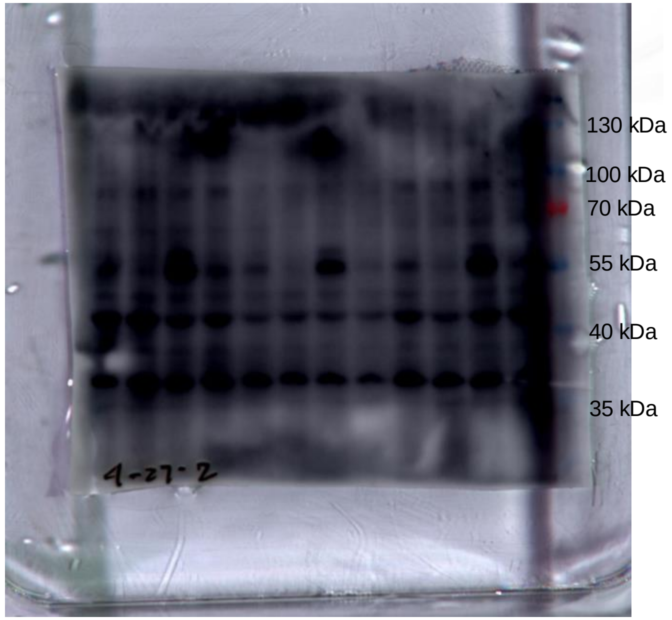
130 kDa
100 kDa
70 kDa
55 kDa
40 kDa
35 kDa

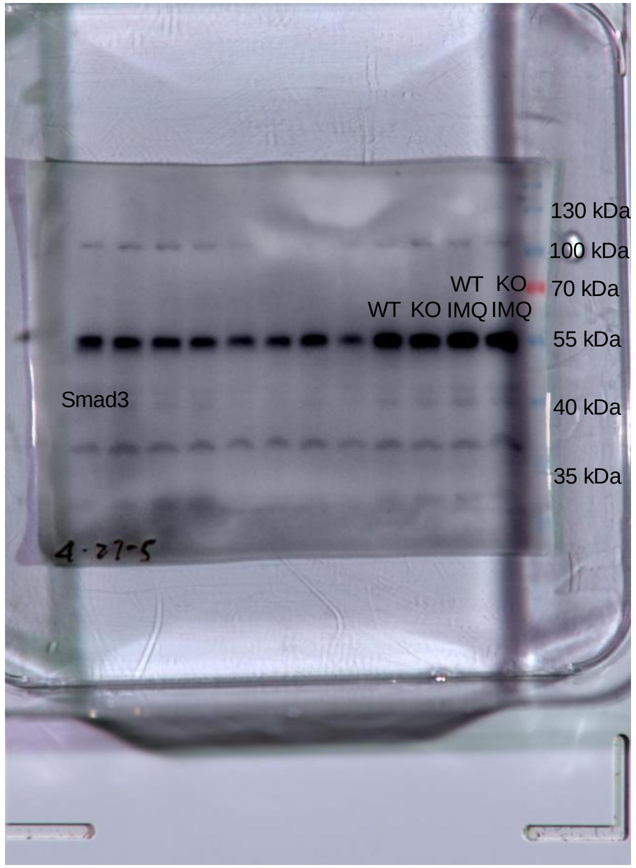
130 kDa
100 kDa
WT KO
70 kDa
WT KO IMQ IMQ
55 kDa
Smad3
40 kDa
35 kDa

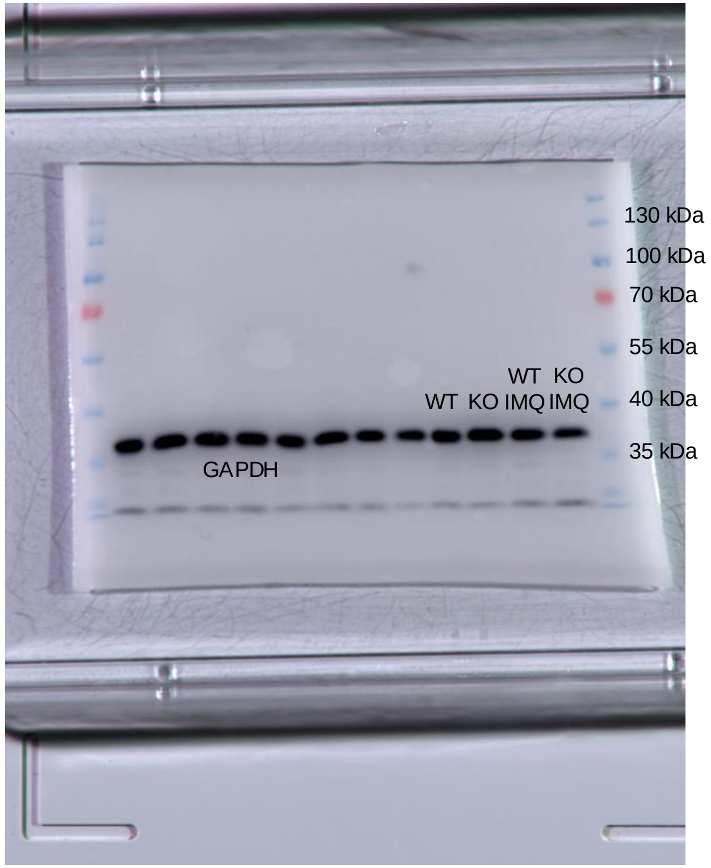
130 kDa
100 kDa
70 kDa
55 kDa
WT KO
WT KO IMQ IMQ
40 kDa
35 kDa
GAPDH

Fig 6D

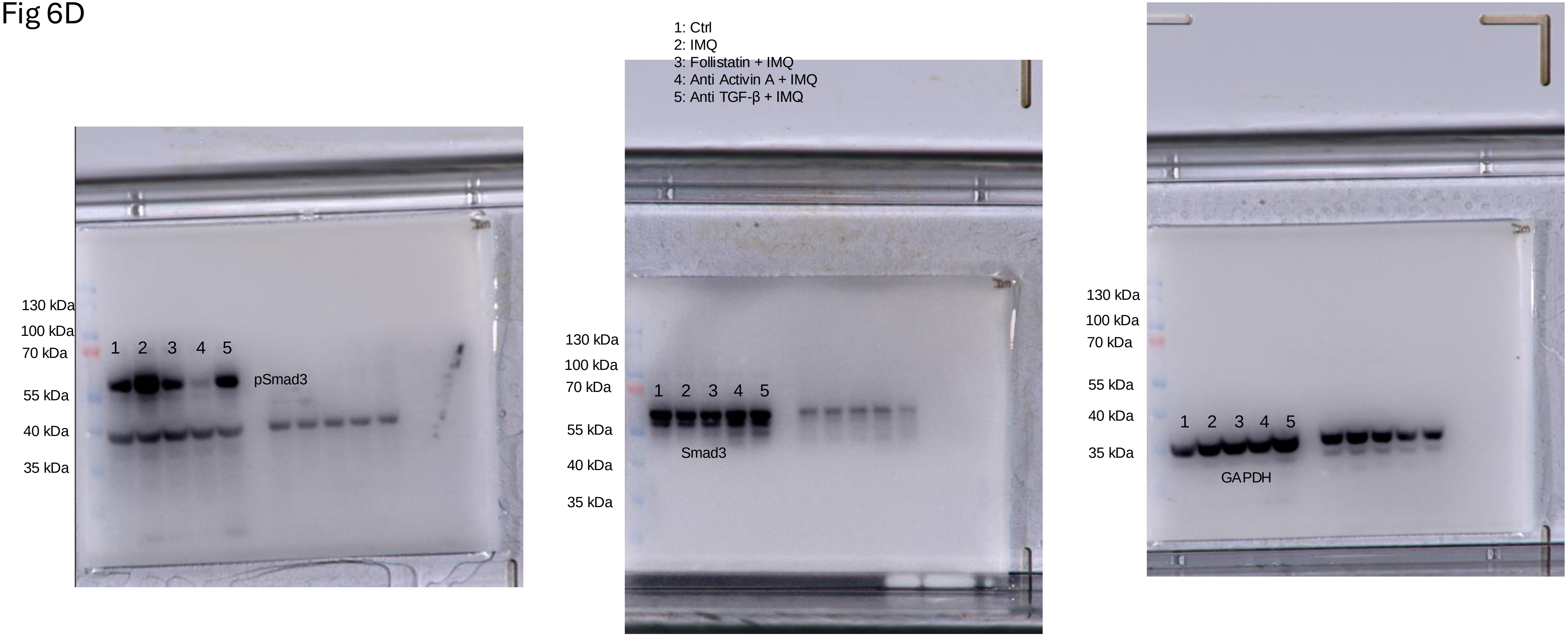
1: Ctrl
2: IMQ
3: Follistatin + IMQ
4: Anti Activin A + IMQ
5: Anti TGF-β + IMQ
130 kDa
100 kDa
70 kDa
55 kDa
40 kDa
35 kDa
1 2 3 4 5
pSmad3
130 kDa
100 kDa
70 kDa
55 kDa
40 kDa
35 kDa
1 2 3 4 5
Smad3
130 kDa
100 kDa
70 kDa
55 kDa
40 kDa
35 kDa
1 2 3 4 5
GAPDH

Fig 6H

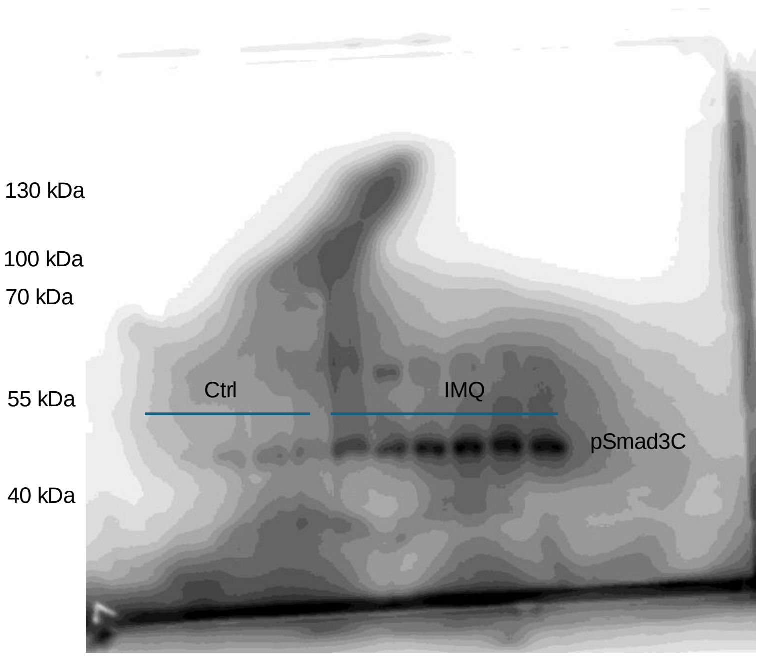

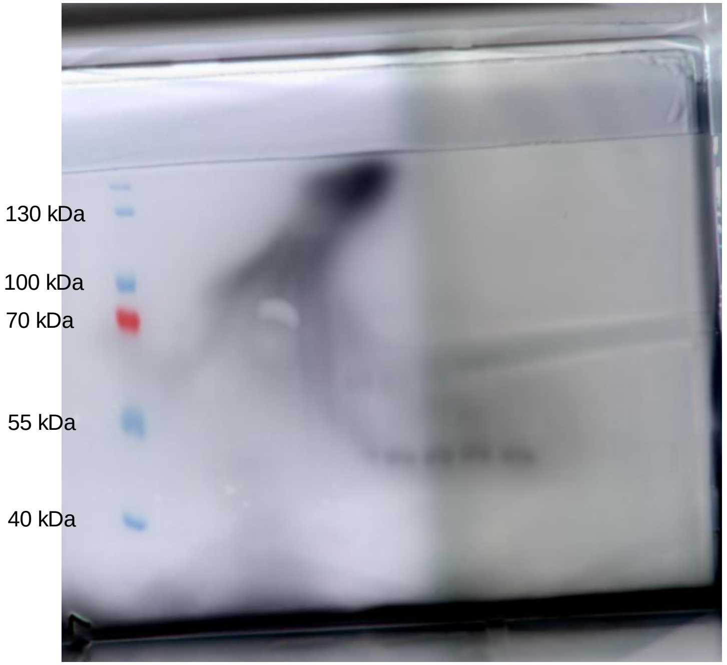

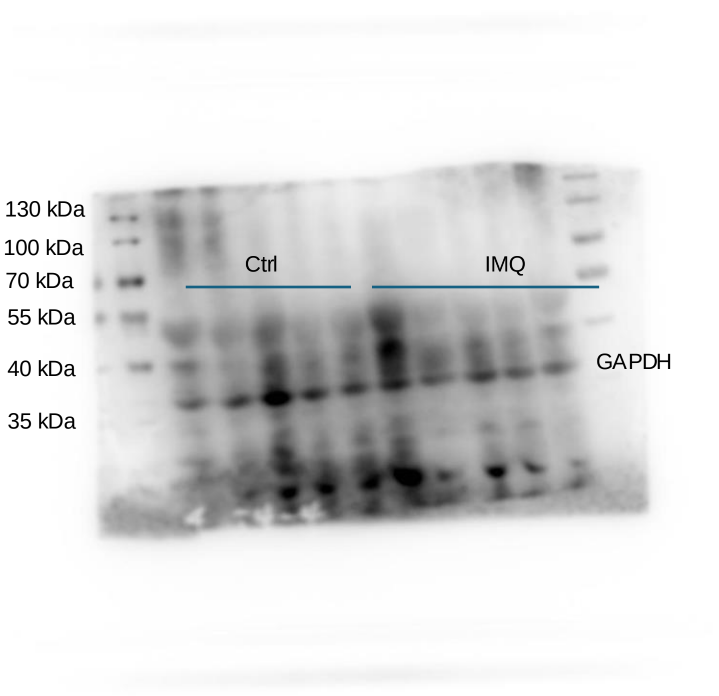

This is the same gel than above but re-exposed in order to see the marker.
Due to overexposure of the substrate some bands started to fade so we used the upper gel for publication.

Fig S11K

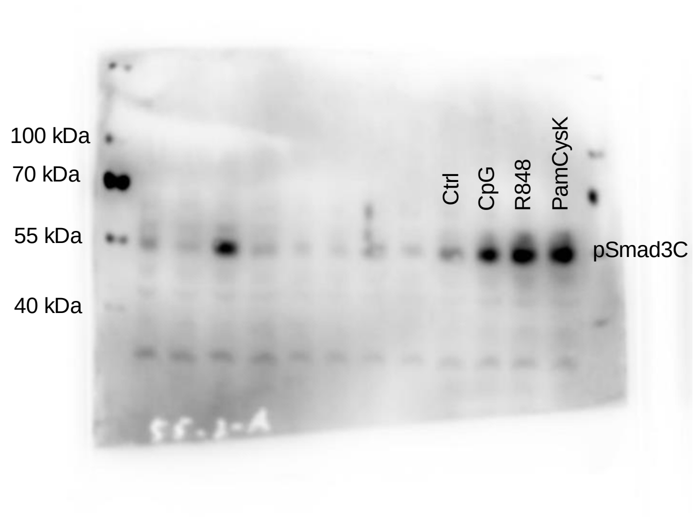
100 kDa
70 kDa
55 kDa
40 kDa
Ctrl
CpG
R848
PamCysK
pSmad3C

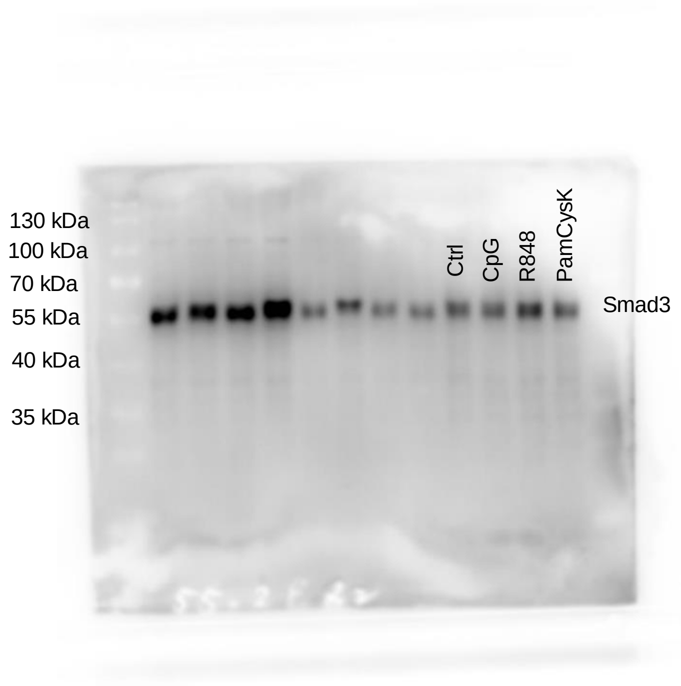
130 kDa
100 kDa
70 kDa
55 kDa
40 kDa
35 kDa
Ctrl
CpG
R848
PamCysK
Smad3

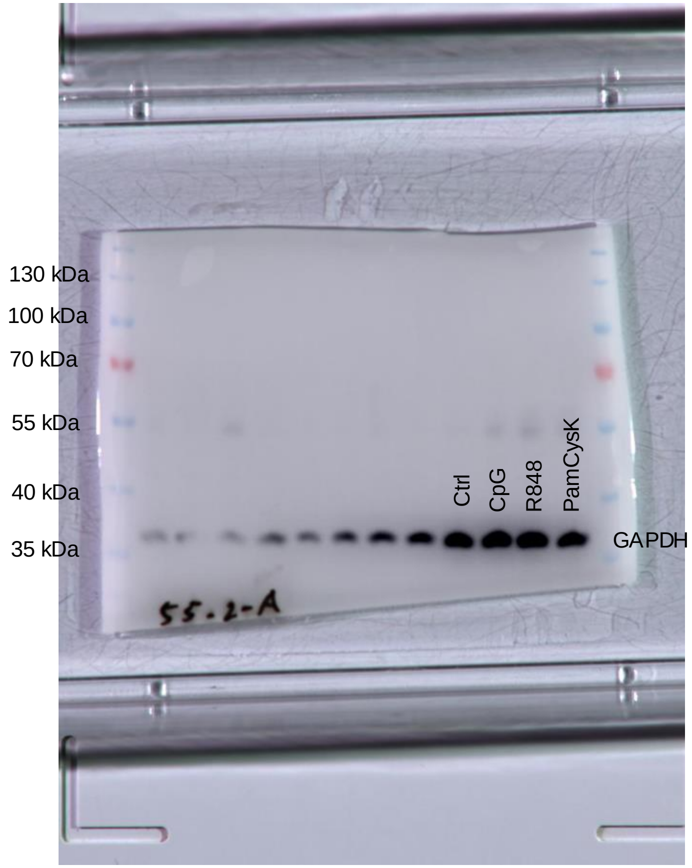
130 kDa
100 kDa
70 kDa
55 kDa
40 kDa
35 kDa
Ctrl
CpG
R848
PamCysK
GAPDH
55.2-A
